# The pinene scaffold: its occurrence, chemistry, synthetic utility, and pharmacological importance

**DOI:** 10.1039/d2ra00423b

**Published:** 2022-04-12

**Authors:** Rogers J. Nyamwihura, Ifedayo Victor Ogungbe

**Affiliations:** Department of Chemistry, Jackson State University 1400 John R. Lynch Street Jackson MS 39217 USA Ifedayo.v.ogungbe@jsums.edu +1-601-979-3719

## Abstract

Plant-based secondary metabolites have been a major source of drug discovery and inspiration for new generations of drugs. Plants offer a wide variety of compound classes, including alkaloids, terpenes, flavonoids, and glycosides, with different molecular architectures (fused bridgehead, bi- and polycyclic, spirocyclic, polycyclic, and acyclic). The diversity, abundance, and accessibility of plant metabolites make plants an attractive source of human and animal medicine. Even though the pinene scaffold is abundant in nature and has historical use in traditional medicine, pinene and pinene-derived compounds have not been comprehensively studied for medicinal applications. This review provides insight into the utility of the pinene scaffold as a crucial building block of important natural and synthetic products and as a chiral reagent in the asymmetric synthesis of important compounds.

## Abstract 2

This review provides insight into the utility of pinene in the synthetic building block and as reagent in asymmetric synthesis.

## Graphical abstract

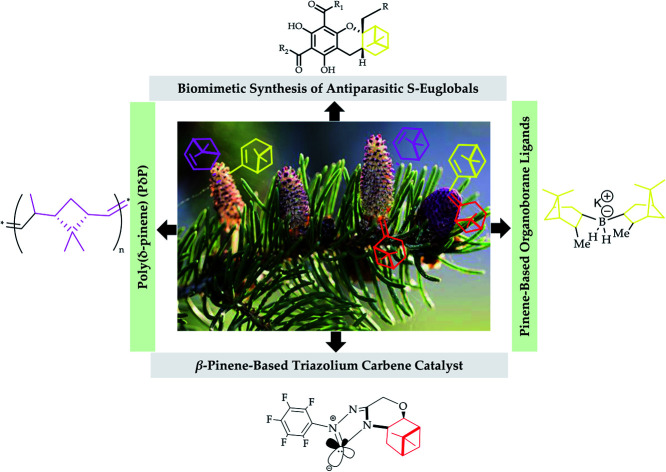

## 1 Introduction

### 1.1 Pinene: nomenclature, structure, and natural occurrence

**Fig. 1:**
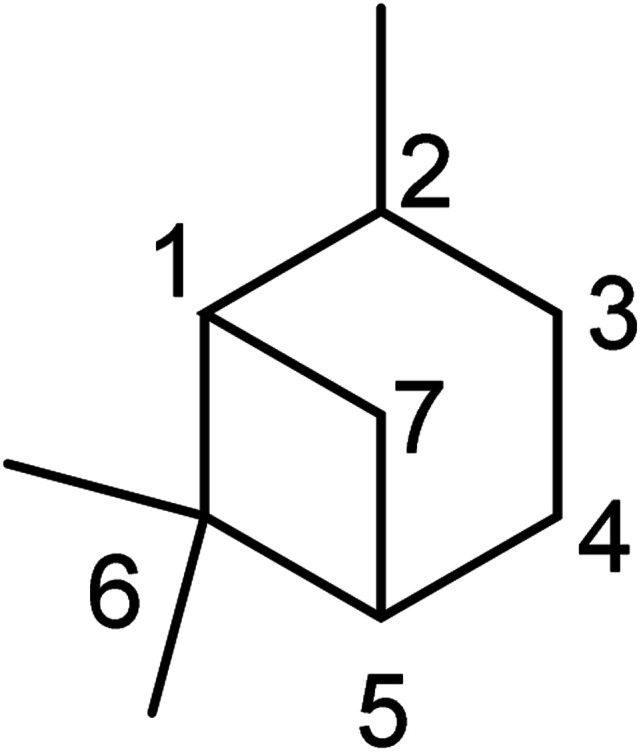
IUPAC numbering of the pinane scaffold.

Pinene is a monoterpenoid hydrocarbon (C_10_H_16_) from isoprene molecules. It has a four-membered ring bridgehead connecting the C-1 and C-5 of the cyclohexyl ring. Thus pinene belongs to the bicyclo [3.1.1] hept-2-ene ring system. The IUPAC name of the pinene skeleton is 2,6,6-trimethylbicyclo [3.1.1] hept-2-ene (shown in Fig. 1 below).

**Fig. 2:**
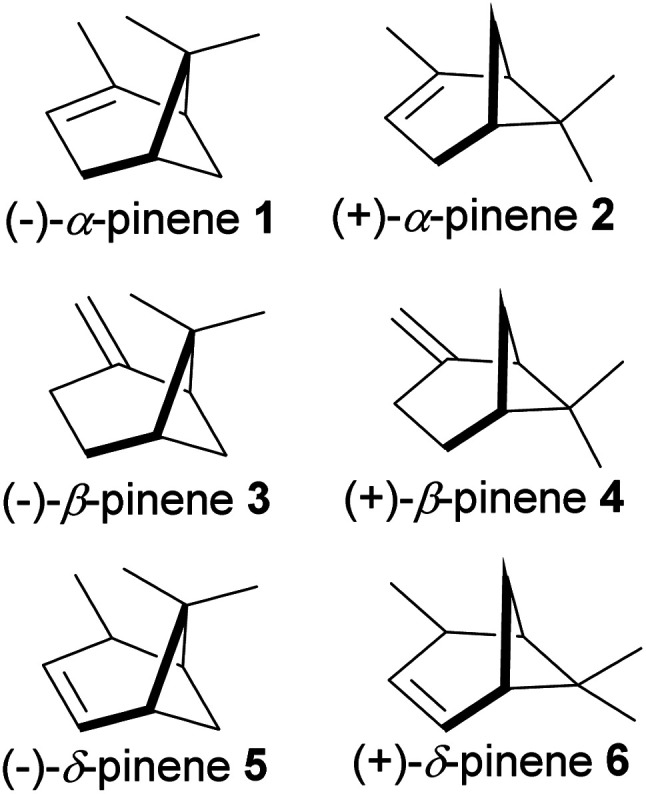
Enantiomers of 3 major isomers of pinene.

The bicyclic ring in pinene comprises cyclohexyl and cyclobutyl hexyl rings fused at C-1 and C-5, forming a bridgehead at the cyclobutyl ring. The bridgehead carbon is demethylated, giving an iconic and distinctive structural appearance. The notable features around pinene's skeletal structure are two chiral centers at positions C-1 and C-5. There are three major pinene constitutional isomers of pinene produced in nature (α-pinene and β-pinene and δ-pinene); because of two chiral centers, each isomer has two major enantiomers; (−)-α-pinene (1), (+)-α-pinene (2), (−)-β-pinene (3), (+)-β-pinene (4), (−)-δ-pinene (5) and (+)-δ-pinene (6) shown in Fig. 2 below.

The relative abundance of each stereoisomer varies within species. It is also common to have racemic mixtures produced by single species. Steuer and co-workers have demonstrated the value of chiral analysis in authenticating essential oils from different pine species and have recently shown that the dominant pinene enantiomer in *Pinus sylvestris* is (+)-α-pinene whereas (−)-α-pinene predominates in *Pinus nigra* and *Pinus mugo*.^1^ α-Pinene is a major component of essential oils from conifers, *Cannabis sativa*, and *Piper*. α-Pinene is a significant component of turpentine, and it is responsible for the strong smell of pine trees. Sharifi-Rad and co-workers have reviewed some of the reported pharmacological actions of α-pinene and β-pinene, including antimicrobial activities (antiprotozoal, antifungal, antibacterial) and antitumor treatment of pancreatitis, gastrointestinal disorders, and hypothermia, as well as anti-convulsant, antioxidants, and anticoagulant activities.^2^ In addition, a recent review on α-pinene by Allenspach and Steuer focused on its pharmacological and synthetic utility for accessing α-turpineol, limonene, and borneol.^3^ Both isomers of α-pinene have been studied for biological activities, which include anti-inflammatory,^4,5^ insecticidal,^6^ nematocidal,^7^ antioxidative,^8^ neuroprotective,^9,10^ gastroprotective,^11^ antimetastatic and apoptotic,^12^ antiapoptotic,^13,14^ antitumor activities. Other studies on the potential use of pinene include for its antimicrobial activities against bacteria,^15,16^ fungi,^17,18^ plasmodium,^19,20^ and viruses.^21^

### 1.2 Pinene: biosynthesis in plants

**Scheme 1:**
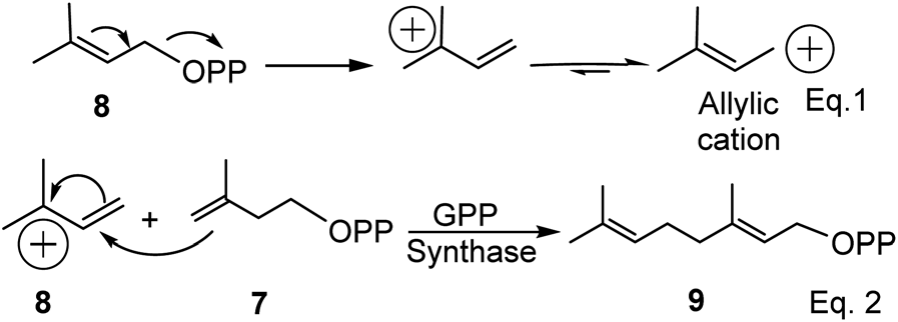
Synthesis of geranyl pyrophosphate (GPP) in plants.

As the name suggests, pinenes are found in pine trees, but other plant species and microbes can produce pinene and related compounds such as carvone. Pinene biosynthesis in plants begins with the activation of isoprene units (C_5_). Activated isoprene exists in equilibrium between two isomers: disubstituted *exo*-olefinic dimethylallylpyrophosphate (DMAPP) 7 and trisubstituted *endo*-olefinic isopentenyl pyrophosphate (IPP) (8), as shown in eqn (1) in Scheme 1 below. DMAPP (7) and IPP (8) are produced *via* mevalonate and deoxyxylulose pathways.^22^ The equilibrium favors 8 because of olefinic stability in the trisubstituted position. In the presence of geranyl pyrophosphate synthase, activated DMAPP reacts with IPP to form geranyl pyrophosphate (GPP) (9), as shown in eqn (2) below.

**Scheme 2:**
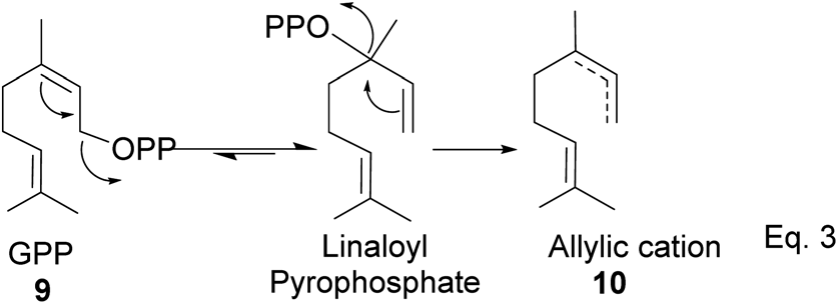
Cationic allylic rearrangement of GPP to allylic cation 10.

GPP isomerizes to linaloyl pyrophosphate, resulting in an allylic cation 10 (Scheme 2 below). The allylic cation 10 can then cyclize depending on the enzyme that acts on it to produce 11–16.

**Fig. 3:**
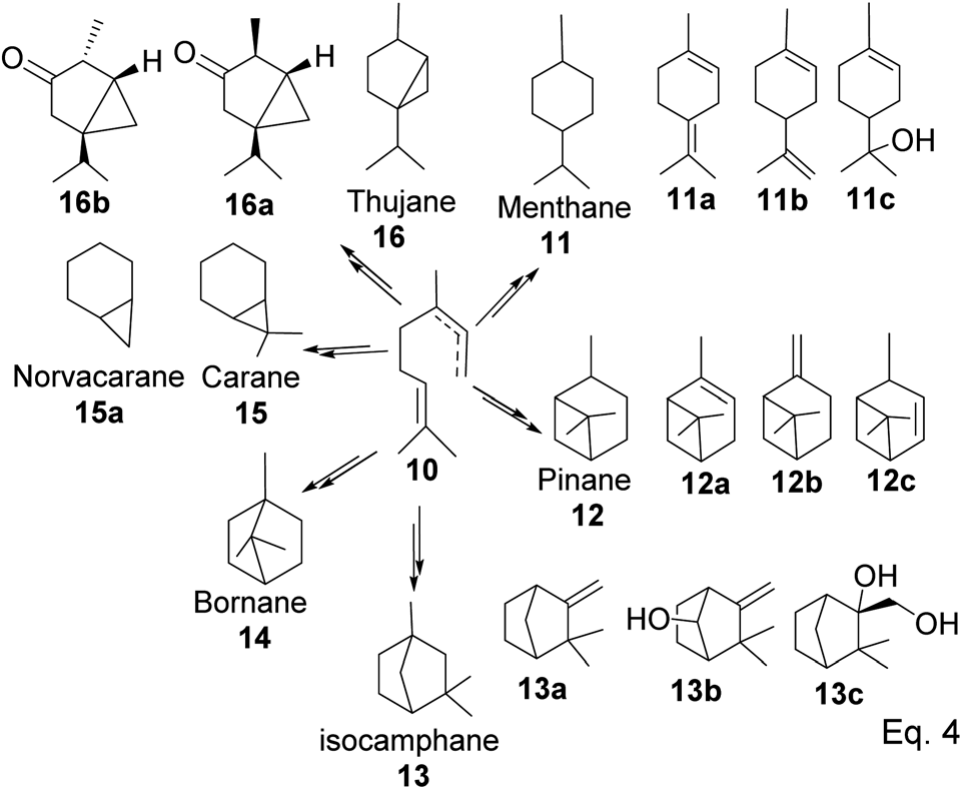
Products derived from cyclization of allylic cation 10.

**Scheme 3:**
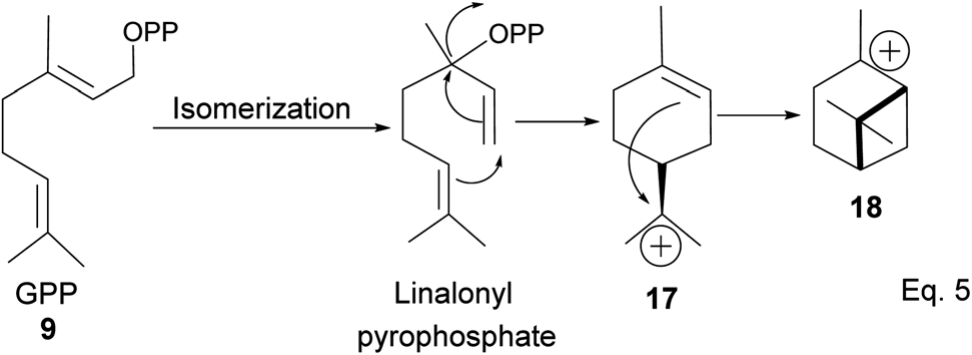
Cyclization of linalonyl pyrophosphate to pinane cation intermediate 18.

After cyclization, the carbocation formed can be terminated through elimination and hydration to afford 11a, 11b, 12a–c, and 13a–c (Fig. 3). Compounds 16a and 16b are produced from the oxidation of alcohols formed from hydration. In pinene synthesis, the cyclization of linaloyl pyrophosphate happens through a six-membered ring transition state and a stable *exo*-3° carbocation (17) to form the isopropyl group. This is followed by a nucleophilic attack of the 3° carbocation to form a bridgehead *endo* 3° cation (18) in the cyclohexyl ring, as shown in Scheme 3 below.

**Scheme 4:**
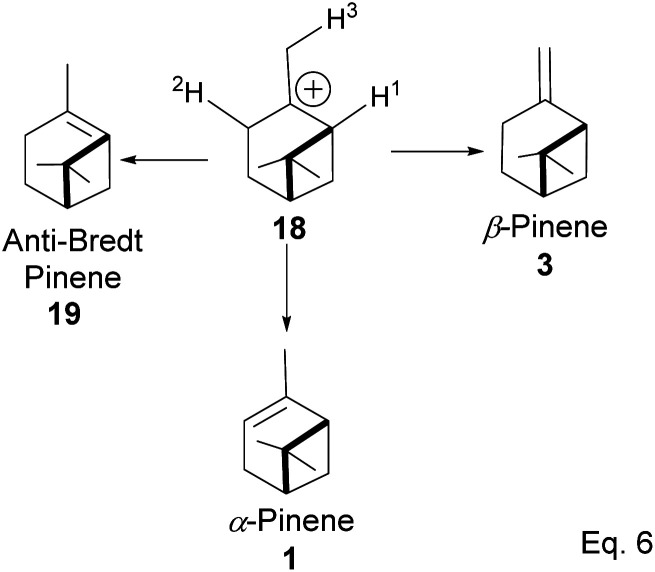
Formation of 3 pinene isomers from olefination of pinane cation.

The α-pinenes (1 and 3) are produced by eliminating methylene protons (H^2^ and H^3^) vicinal to the carbocation. This is the favored product because the olefin is trisubstituted. It is important to note that eliminating methine proton (H^1^) at the bridgehead would result in a highly strained anti-Bredt compound (19). The loss of methyl proton produces β-pinene. *Exo*-olefins are stable but less favored than *endo* olefins because the former is less substituted than the latter (Scheme 4).

**Scheme 5:**
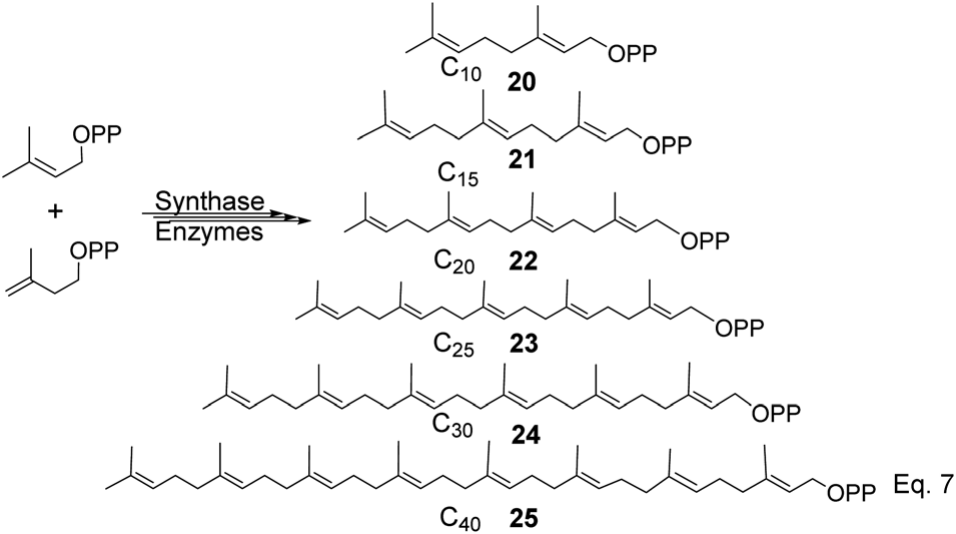
Precursors for synthesis of cyclic terpenes (pinene, steroids, and triterpenoids).

To produce terpenes and terpenoids of higher molecular weight, chain elongation increases by the addition of each isoprene unit (IPP); therefore, monoterpene consists of C_10_(20) carbon atoms di-, tri-, tetra-consists of C_20_(22), C_30_(24), and C_40_(25) carbon units. Triterpenes or squalene 24 (C_30_) are precursors in the biosynthesis of steroids and triterpenoids, as shown in Scheme 5 below.

**Scheme 6:**
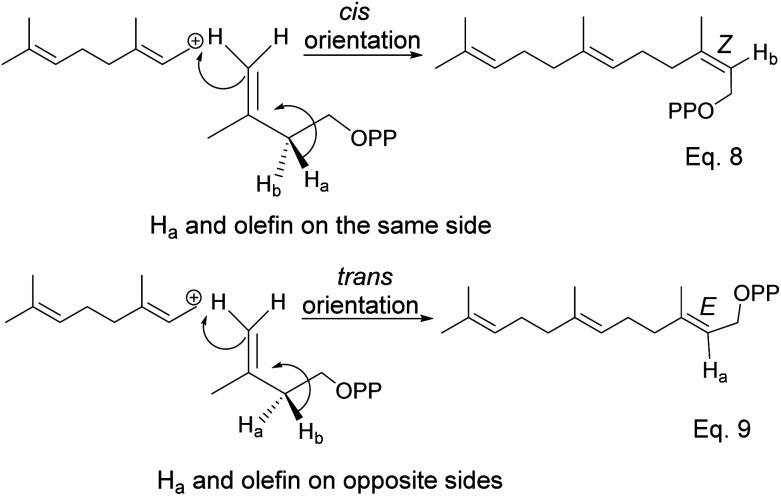
Stereospecific dehydrogenation of farsenyl pyrophosphate (FPP) to form *E* and *Z* isomers.

The addition of IPP to allylic cation and loss of proton in IPP occur concertedly. Moreover, proton loss in IPP is *stereospecific*; therefore, stereochemistry from olefination is dictated by which of vicinal protons is removed, as shown in Scheme 6 below.

Furthermore, the complexity of cyclic compounds formed dramatically increases with each addition of isoprene units. This includes steroids of different scaffolds such as lanosterol, cycloartenol, cucurbitane, and triterpenes^23^ of various types, including hopane, lupane, oleanane, and ursane.^24^

### 1.3 Pinene: roles and effects in plants

Secondary metabolites present in essential oils help plants communicate, whereas compounds such as bitter and toxic alkaloids discourage herbivores and pests and act as antimicrobial agents against parasites such as fungi and bacteria.^25^ Isomers of pinene appear to have deleterious effects on plant pests^26,27^ and influence plant communication.^28^ Unlike animals, plants lack mobility, which presents a challenge against pests and herbivores that prey upon them. A few investigators have pursued studies on the effect of α-pinene and β-pinene in various plant species. Kohli and co-workers have reported that α-pinene inhibits radicle growth in *Cicer arietinum* by increasing solute leakage from roots and increasing the levels of malondialdehyde (MDA), proline, and H_2_O_2_,^29^ and β-pinene inhibited the shoot and root growth of *Phalaris minor*, *Echinochloa crusgalli*, *Cassia occidentalis*.^30^ β-Pinene has also been shown to reduce Cr(vi)-induced accumulation of reactive oxygen species in maize.^31^ In addition, α-pinene was found to inhibit seed germination in *P. sativum and Zea mays*.^32,33^

## 2 Pinene chemistry: functionalization and reactions

The olefin functional is the primary target in pinene functionalization because it is the most reactive part of the molecule. Pinene functionalization in organic synthesis poses some serious challenges despite its relatively high reactivity. The proximity of the strained cyclobutyl bridgehead (at C-1 and C-5) to the olefin functional group is a significant challenge. The 1,5 carbon–carbon (C–C) bond connection of bridgehead carbon to the cyclohexyl ring creates angular strain due to the cyclobutyl ring. The cyclobutyl C–C–C bond angle is 88° instead of 90° is due to the ring's attempt to relieve torsional strain caused by eclipsing hydrogen atoms adjacent to each other. Therefore, the pinene scaffold is prone to isomerization and 1,2-Wagner–Meerwein rearrangement.^34^

### 2.1 Pinene epoxidation

**Scheme 7:**
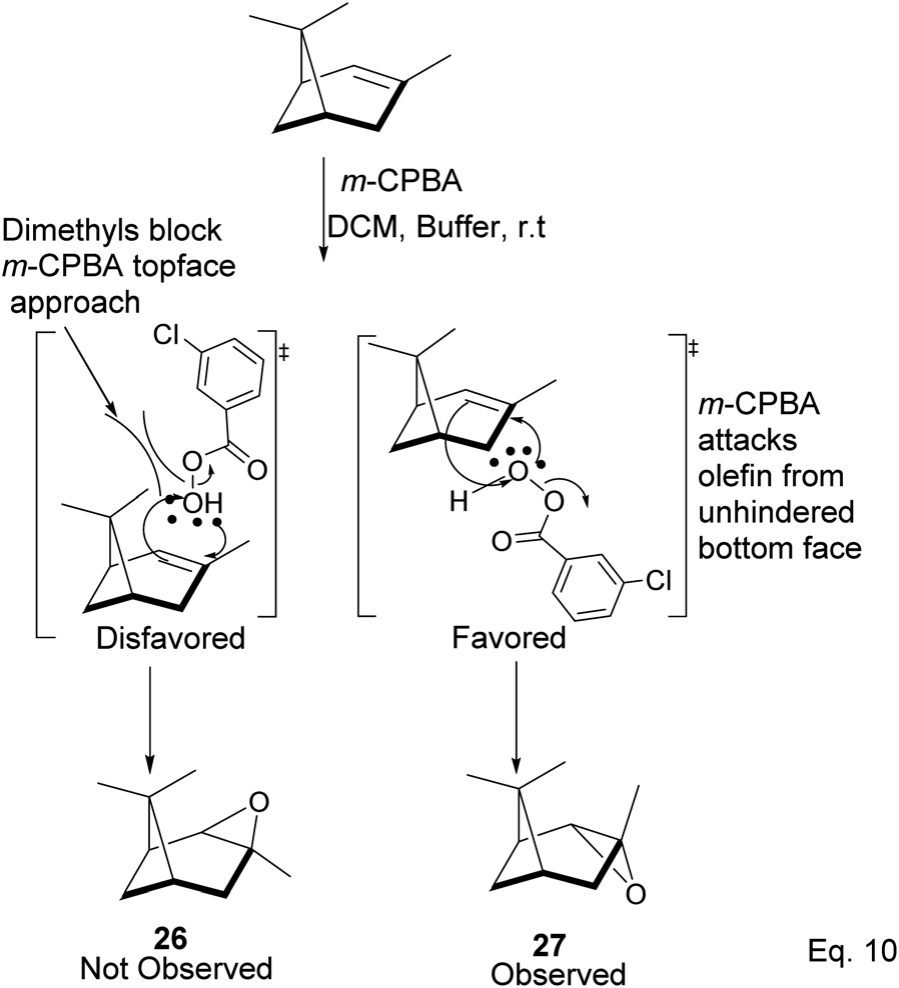
Stereospecific epoxidation of α-pinene by *m*-CPBA.

**Scheme 8:**
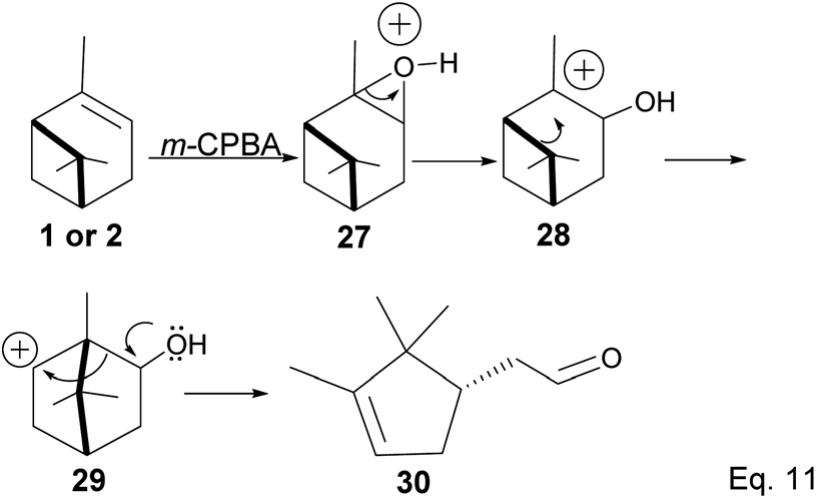
Favorskii type rearrangement of norborane cation (29) to acetaldehyde (30).

Pinene epoxidation is highly stereospecific. Epoxidation happens on the opposite side of the bridgehead to produce a single pinene oxide isomer (27). Epoxide 26 shown in Scheme 7 is not produced because the dimethyl on the cyclobutyl bridgehead impedes oxidizing reagents such as *m*-CPBA from attacking double bonds from the top face. The dipole moment in C–O and angle strain in epoxide makes pinene oxide even more strained and susceptible to ring-opening through the epoxide. Spontaneous ring-opening has a significant impact on pinene oxide yield. A higher yield of pinene oxide is desired because it serves as a precursor in synthesizing campholenic aldehyde (30) and *trans*-carveol (32)^35^ found in fragrances and used as food flavor. Pinene oxide, like pinene, is vulnerable to cationic rearrangement. Epoxide ring-opening in the presence of Lewis acid leads to pinene 3° cation (28). Despite being a 3° cation (28), the pinene cyclobutyl ring opens *via* 1,2-alkyl shift or fracture to relieve angle strain. The 1,2-alkyl shift causes a cyclobutyl ring expansion to produce a norborenyl 2° carbocation (29). Note that in the norborenyl cation, the carbocation and hydroxyl group have an allylic relationship (Scheme 8). Therefore, carbonylation of the hydroxyl group to an aldehyde and olefination of the norborenyl 2° cation are the driving forces for forming a relatively more stable 2-(2,2,3-trimethylcyclopent-3-en-1-yl) acetaldehyde (30).

**Scheme 9:**
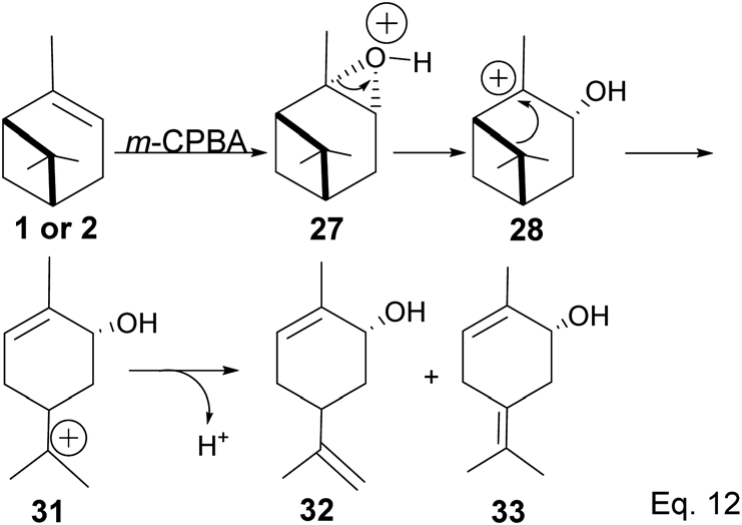
Epoxide rearrangement *via* 1,2-σ-alkyl shift of pinene bridgehead to form terpineol derivatives.

Fracturing the C–C bond in the dimethylated cyclobutyl bridgehead is an alternative path to relieve angle strain. This alternative path leads to the formation of a terpenyl 3° carbocation (31), which can undergo intramolecular olefination through *vicinal* proton elimination or intermolecular hydration. Intramolecular olefination is entropically favored over intermolecular hydration. Olefination of 31 can proceed to produce kinetic product 2-methyl-5-(prop-1-en-2-yl) cyclohex-2-en-1-ol (32) and thermodynamic product 2-methyl-5-(propan-2-ylidene) cyclohex-2-en-1-ol (33) (Scheme 9).

**Scheme 10:**
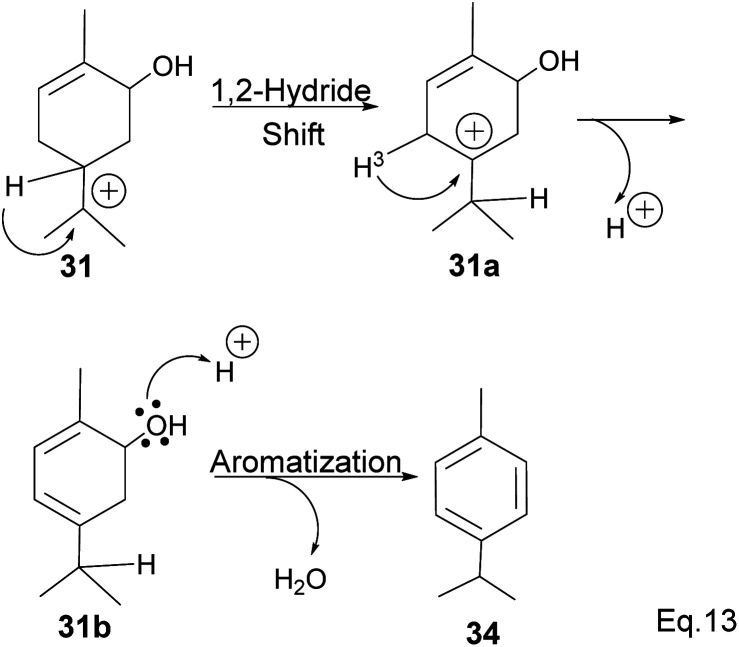
Aromatization of terpineol to *p*-cymene.

**Scheme 11:**
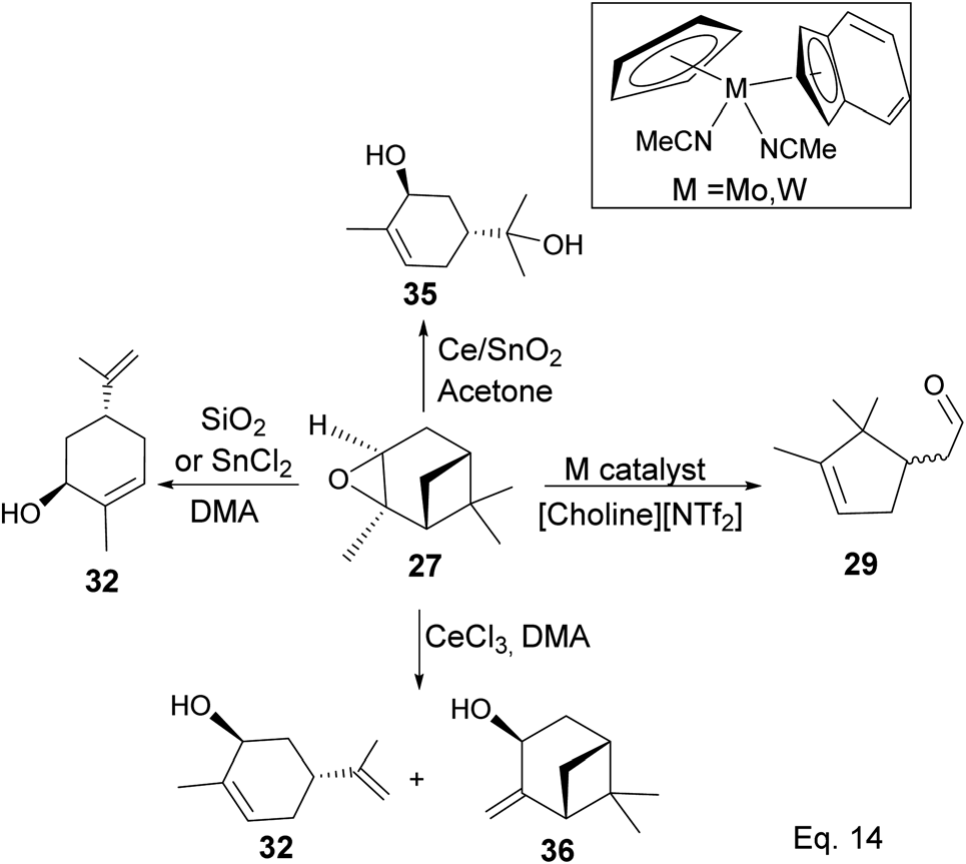
Metal-mediated transformation of pinene oxide.

In addition, a 1,2-hydride shift in 31 leads to an *endo* 3° carbocation, and the loss of H-3 leads to the formation of a conjugated olefin (31b), which aromatizes to cymene (34) through dehydration (Scheme 10). Pinene oxide can be transformed into several products depending on the solvent and catalyst used. For instance, in acetone Ce/SnO_2_ mixture, pinene oxide isomerizes to *trans*-sorbrerol (35) (Scheme 11), whereas in the presence of SnCl_2_ or SiO_2_ in dimethylacetamide (DMA), *trans*-carveol is produced. Replacing SnCl_2_ or SiO_2_ with CeCl_3_ in DMA, two *exo*-olefinic alcohols, *trans*-carveol (32) and *trans*-pinacarveol (36), are formed.^36^ Product selectivity is solvent-dependent. Both basicity and polarity are determinant factors in product distribution. For example, in DMA, with Sn/Ce or SiO_2_ as the catalyst, the major product was *trans*-carveol (32), while campholenicaldehyde (30) and *trans*-pinocarveol (36) were minor products. However, in acetone, with SiO_2_ as the catalyst, *trans*-sorbrerol (35) (70%) was the major product, while campholenicaldehyde (30) (16%) and *trans*-carveol (32) (13%) were minor products.

**Scheme 12:**
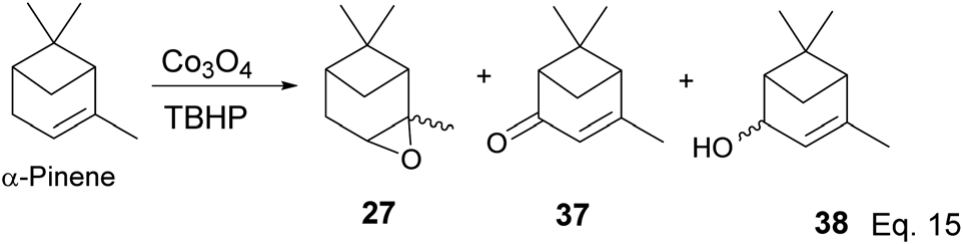
Cobalt oxide-promoted transformation of α-pinene.

Some interesting metal-catalyzed epoxidation of α-pinene has been observed and reported. For example, Lu and Tang's epoxidation of α-pinene using nanosized CoO_*x*_ such as Co_3_O_4_ in the presence of dry air and at 100 K yielded 87.68% pinene oxide (27) with a 70.75% conversion rate. The side products from the reaction include verbenone (37) and verbenol (38), and there was no monocyclic product observed (Scheme 12).^37^ Conversion rate steadily decreases, whereas selectivity increases with the incorporation of SnO_*x*_ surfactants.

**Fig. 4:**
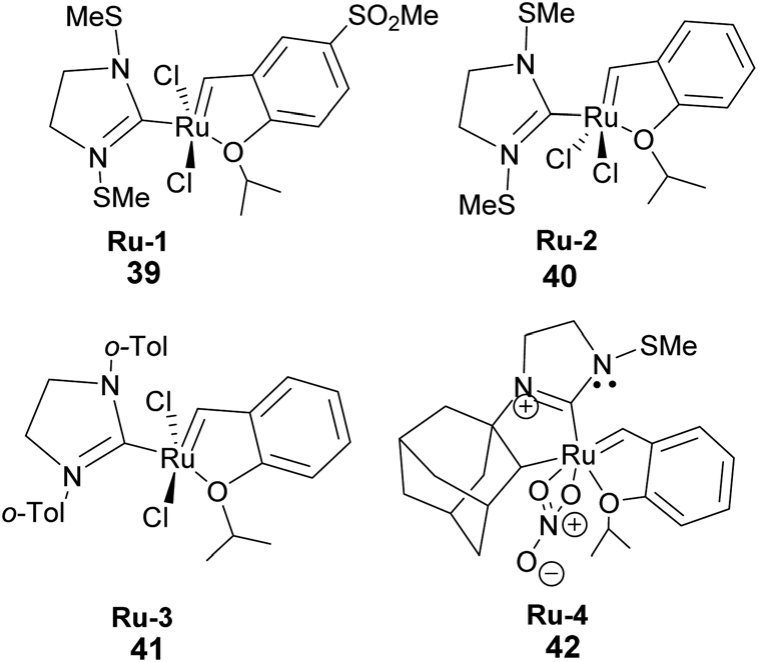
Grubb's ruthenium-based catalyst used in metathesis.

To date, few synthetic methods can be used in pinene transformation without compromising the integrity of the bicyclic ring structure. Metal-catalyzed metathesis by late transition metals such as ruthenium is one of the effective ways to transform the pinene scaffold without unwanted side products. However, the use of ruthenium containing catalysts such as Grubb's 1^st^, 2^nd^, and 3^rd^ generation catalyst (Fig. 4) have one major drawback the bulky cyclic ligands surrounding the ruthenium center, which influences the catalyst's stereo-electronic properties is also an Achilles heel and cannot be used in transforming trisubstituted olefinic α-pinene, the most abundant pinene^38^ isomer in higher plants.

The catalyst's bulky ligands and the methyl group at C-1 in pinene impede ruthenium access to the olefin during metal insertion. Therefore, as we shall see later in this review, isomerization of α-pinene to δ-pinene is required to harness the synthetic utility of pinene and catalytic potential of Grubb's catalyst.

## 3 Pinene in the synthesis of key intermediates and other small molecules

Pinene's structure, chemistry, abundance in nature, and intrinsic antimicrobial activities make it an affordable and versatile renewable and non-petrochemical molecule for human use. As a result, pinene isomers and their derivatives are used as bulk chemicals in pharmaceutical and material science (polymer and cosmetics) industries. This section provides an overview of the synthesis of some small molecules and synthetic intermediates derived from pinene isomers.

### 3.1 Pinene in the synthesis of carvone

Carvone (*p*-mentha-6,8-dien-2-one) isomers are important constituents of essential oils. Due to its flavor, carvone is used in the food industry and for aromatherapy. Carvone has also been well studied for potential effects on animal physiology and health. For example, Alsanea and co-workers have shown that *S*- (+)-carvone blocked weight gain, fat accumulation in the liver, and insulin resistance in mice fed with a high-fat diet. Furthermore, it improves the expression of macrophage gene markers (*F4/80*, *Cd11b*, *Cd11c*, *Cd206*, and *Tnf*-α) in white adipose tissue, at the same time suppresses the expression of genes (*Pparγ2*, *Scd1*, *Cd36*) responsible for lipid synthesis and transportation in the liver.^39^ (*S*)-carvone induces the expression of detoxifying enzymes such as glutathione *S*-transferases (GSTs)^40^ and inhibits nitrosamine-induced carcinogenesis.^41,42^ The α,β-unsaturated ketone in carvone acts as a Michael acceptor, thus explaining carvone's ability to induce the expression of GSTs.^43^ The antioxidant activities of *S*-carvone have been studied and reported by Eine and co-workers. *S*-Carvone was shown to have a remarkable ability to scavenge free radicals, and its antioxidant activity exceeded that of butylated hydroxyanisole (BHA), α-tocopherol, and butylated hydroxytoluene (BHT).^44^*S*-carvone can be prepared by fractional distillation of caraway oil. However, the synthesis of carvone from cheap and abundant natural sources such as pinene has attracted the attention of chemists due to carvone's versatility in synthesizing other important terpene congeners such as carvomethanol, carvomenthone, limonene, as well as more complex natural products.

**Scheme 13:**
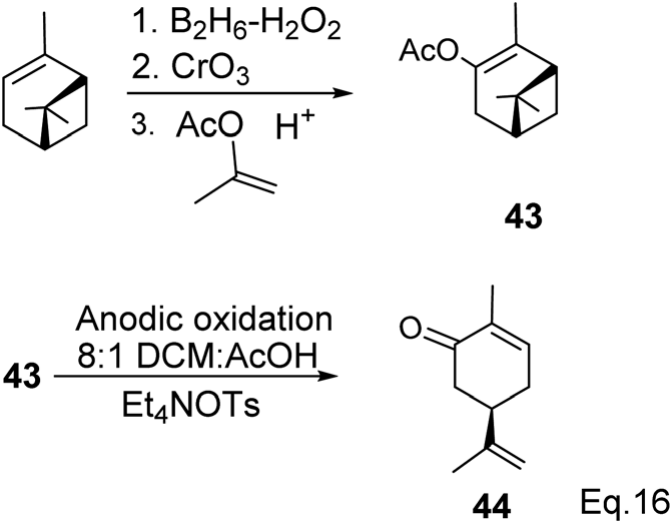
Anodic oxidation of α-pinene enol acetate.

**Scheme 14:**
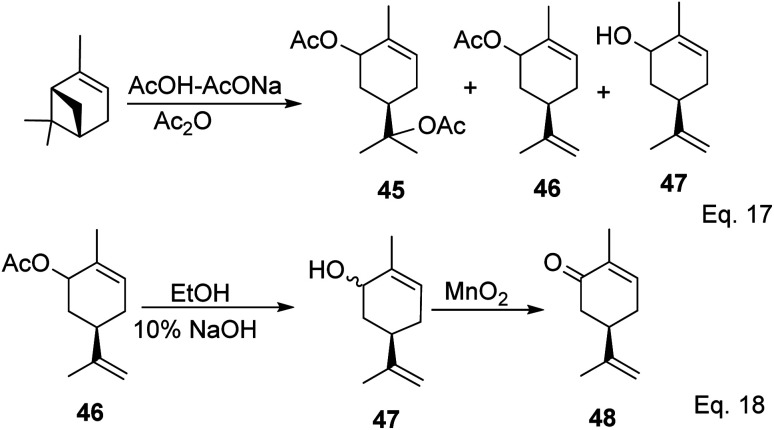
Synthesis of carvone from anodic oxidation of α-pinene.

Carvone can be prepared from pinene through anodic oxidation of pinene enol acetate (43) derived from oxidative hydroboration of α-pinene with H_2_O_2_ and CrO_3_ in the presence of enol acetate (Scheme 13) as demonstrated by Shono^45^ and his group. In Shono's anodic oxidation of pinene enol acetate (43), carvone (44) was obtained in 64% yield when 8 : 1 DCM-AcOH and tetraethylammonium *p*-toluenesulfonate (Et_4_NOTs) were used. Macaev and co-workers also reported the synthesis of carvone and cryptomerlone *via* anodic oxidation of pinene in AcOH–AcONa solution using RuO_2_, Pt, or TiO_2_ anode and carbon electrode.^46^ Unlike Shono's method, which produced several side products, Macaev's method yielded sobrerol diacetate (45) as a side product and allylic alcohol (47) in 12% yield. Hydrolysis of the allylic acetate (46) resulted in the corresponding allylic alcohol (47) in high yield, and oxidation of 47 with MnO_2_ afforded 65% of carvone (Scheme 14).

**Scheme 15:**
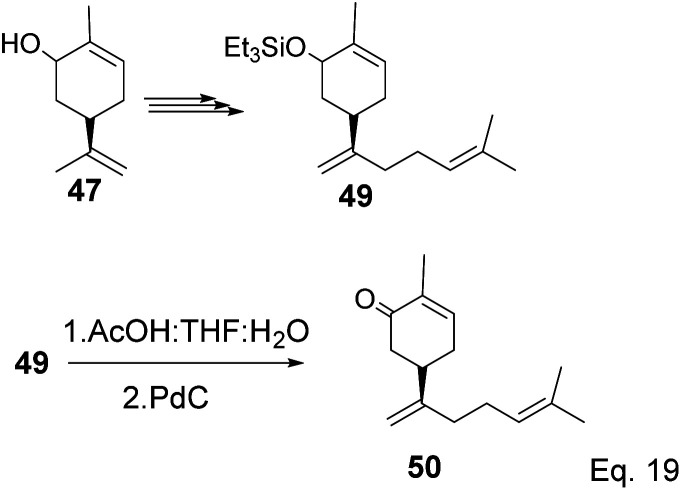
Synthesis of cryptomerlone 50 from α-pinene allylic alcohol.

**Scheme 16:**
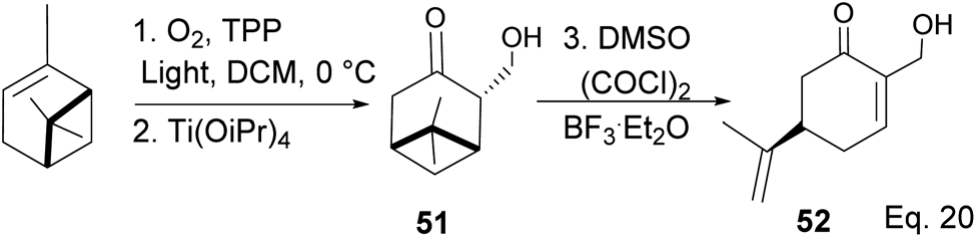
Synthesis of (R)-7-hydroxycarvone (52) from α-pinene.

The key intermediate in cryptomerlone synthesis, 49, was obtained in 3 steps by protecting the allylic alcohol in 47 with Et_3_SiCl followed by bromination and prenylation reactions. Subsequent removal of the Et_3_Si protecting group from 49 (Scheme 15) followed by Pd*/*C dehydrogenation led to cryptomerlone (50) in 20% yield. 52 was prepared in 8% yield from perillyl acetate using CrO_3_,^47^ and the methyl ester derivative was prepared in 8 steps from carvone.^48^ McIntosh and co-workers reported significant improvements in synthetic steps and an overall yield to produce 52. Using photooxygenation and fragmentation reactions, 52 was prepared from (*S*)-α-pinene in 3 steps (Scheme 16) *via* an alcoholic ketone 51. The fragmentation-induced oxidation of 51 afforded (*R*)-7-hydroxycarvone 52.

**Scheme 17:**
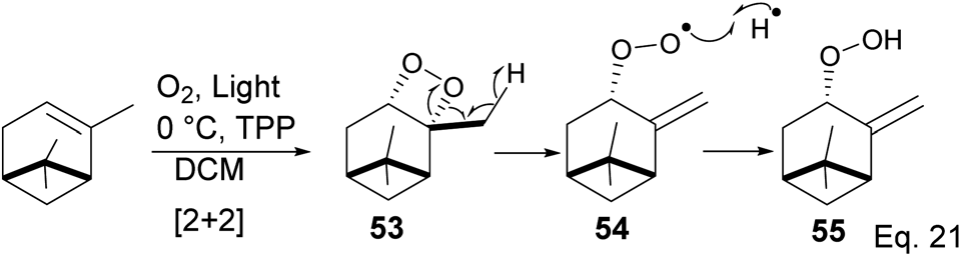
Light catalyzed peroxidation of α-pinene by oxygen.

In the photooxygenation of pinene carried out in the presence of tetraphenylporphine (TPP) sensitizer, the singlet oxygen attacked the olefin from the less hindered bottom face (Scheme 17). The reaction proceeds through a [2 + 2] cycloaddition involving a diradical oxygen intermediate and an unstable 1,2-dioxetane 5, which undergoes homolytic C–O cleavage to 54 is quenched by hydrogen radical to peroxide 55.

**Scheme 18:**
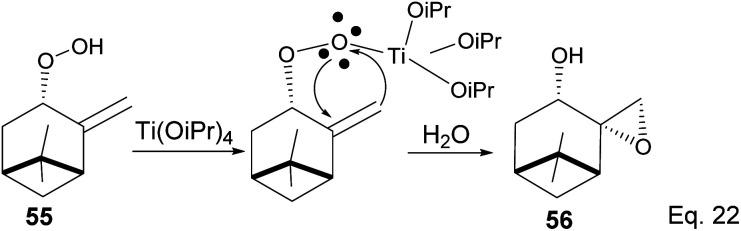
Ti(OiPr)_4_-mediated intramolecular epoxidation of allylic peroxide.

The presence of Ti(OiPr)_4_ in the reaction mixture created a Ti-peroxide complex in the transition state, thus facilitating a stereoselective epoxidation of *exo*-olefin by the peroxide to form epoxide 56 in 92% yield (Scheme 18).

**Scheme 19:**
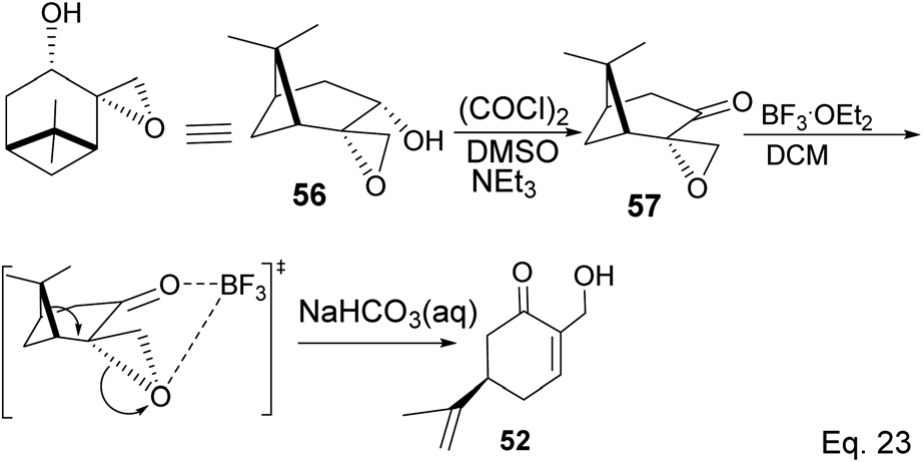
BF_3_·Et_2_O-promoted intramolecular ring opening of pinene oxide to (*R*)-7-hydroxycarvone.

Swern oxidation of epoxy alcohol 55 produced a ketone epoxy 57, and (*R*)-7-hydroxycarvone was produced through BF_3_OEt_2_-assisted cleavage of the epoxy group in 57 (Scheme 19).

### 3.2 Pinene in the synthesis of pinene-containing conjugated acid derivatives

**Scheme 20:**
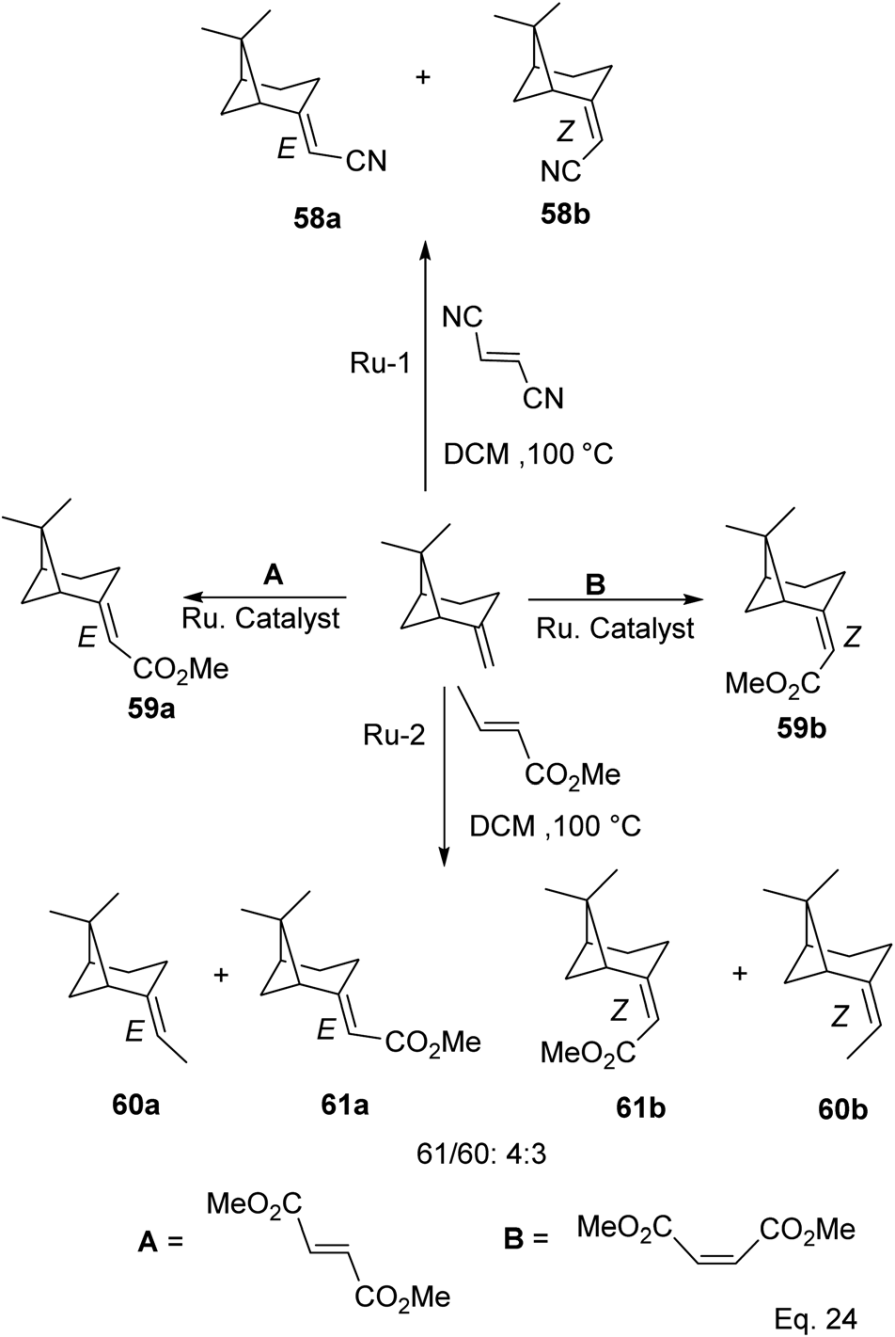
Products derived from ruthenium-assisted metathesis of δ-pinene.

Cross metathesis of β-pinene allows the addition of carbon atoms and introduction of polar functional groups such as ester, nitrile, and acetate. Thus important synthetic compounds such as nopol and amino derivatives can be cheaply and easily produced from pinene. For example, ruthenium-mediated cross-metathesis of β-pinene with maleonitriles and acrylic olefins to produce their corresponding *E* and *Z* stereoisomers of acetonitrile (58a–b) and esters (61a–b) was reported by Bruneau and co-workers (Scheme 20).^49^

### 3.3 Pinene in the synthesis of nopol, myrtanal, and myrtenol

**Scheme 21:**
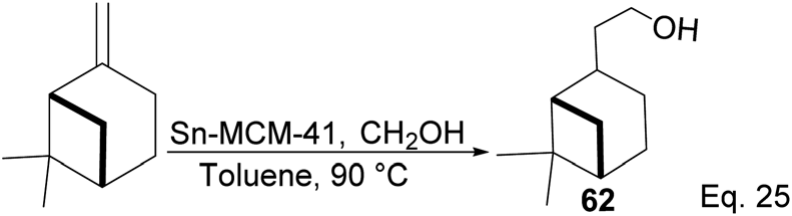
Synthesis of nopol from β-pinene.

Nopol can be obtained from pinene through the Prins reaction using formaldehyde. Bain^50^ pioneered Prins reaction in nopol synthesis using ZnCl_2_ as a catalyst at a relatively high temperature (115–120 °C) for several hours. Then, Correa and co-workers improved the synthesis using a Tin-grafted catalyst (Sn-MCM-41)^51^ (Scheme 21). The reaction showed a 61.3% conversion rate and 98.7% selectivity.

**Scheme 22:**
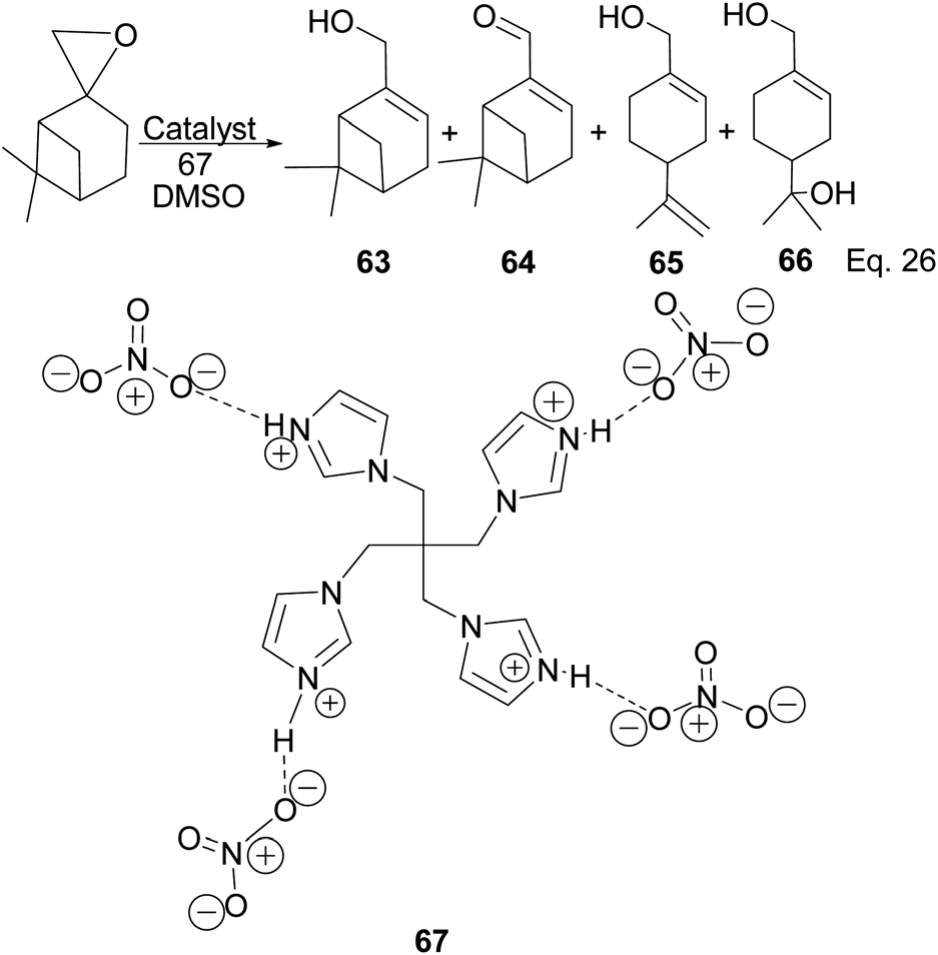
Myrternol, myternal, and terpineol derivatives of β-pinene oxide.

Myrtenol and its aldehyde derivative are key intermediates in the synthesis of bioactive aminol such as 2-phenyliminooxazolidines and spiro-fused oxazolidin-2-one.^52^ The two compounds and perillyl alcohols can be easily prepared from pinene oxide. Yin and his group reported the synthesis of myternol 63 (18.6%), myternal 64 (7.1%), perillyl alcohols 65 (47%), and diol 66 (15.8%) using tetraimidazolium nitrate 67 catalyzed isomerization of pinene oxide (Scheme 22).

### 3.4 Pinene in the synthesis of (+)-nopinone

**Scheme 23:**
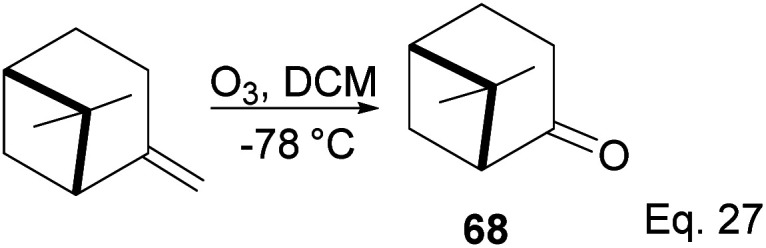
(+)-Nopinone synthesis from ozonolysis of δ-pinene.

(+)-Nopinone is an important intermediate in synthesizing complex natural products and as a ligand in metal complex catalyst used in asymmetrical synthesis.^53^ (+)-nopinone 68 can be prepared from β-pinene by oxidation with ozone at low temperature (Scheme 23). The reaction involves [2 + 3] cycloaddition of ozone. It is concerted without any cationic intermediate. Thus it proceeds without any isomerization of pinene through either 1,2-cyclobutyl ring expansion or disintegration of pinene bridgehead.

### 3.5 Synthesis of terpineol from α-pinene by liquid–liquid model catalysis

**Scheme 24:**
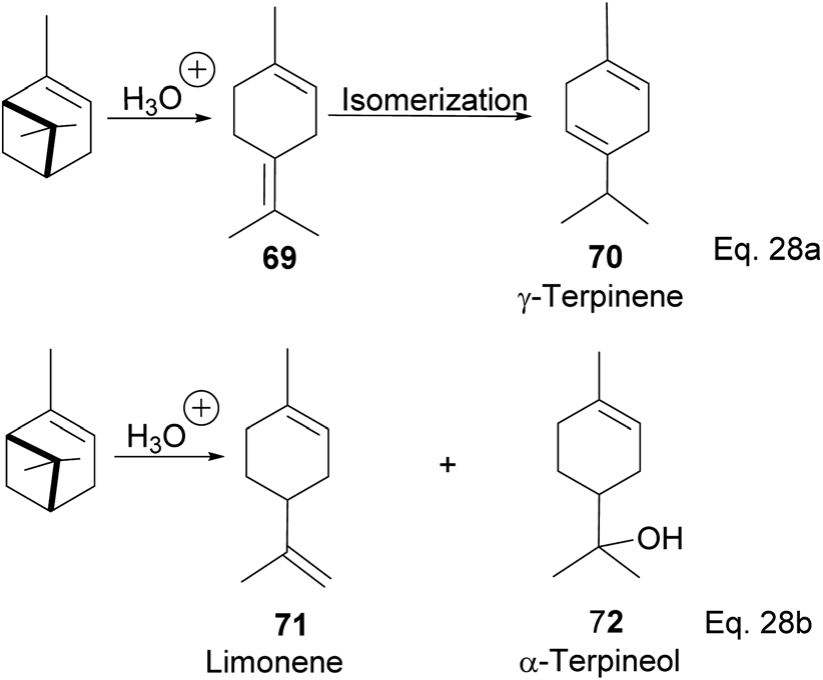
Acid-catalyzed hydration of α-pinene to monocyclic terpenoid derivatives.

Terpineol is a terpene of high economic importance in the pharmaceutical industry and cosmetics. It is used in perfumes, as an insect repellant, as an antimicrobial, and for the production of copolymers. It is produced by the hydration of α-pinene with aqueous sulfuric acid industrially. However, the relatively low conversion rate of pinene to terpineol and poor selectivity over side products remains a problem in the hydration reaction. Thus, several homogenous acid–base catalysts have been studied to solve the problem. For example, in the synthesis of terpineol (72) shown in Scheme 24 below,^54^ Aguilar and co-workers used aqueous chloroacetic acid and oxalic acids to catalyze the hydration of pinene through cation rearrangement of the α-pinene ring. Rearrangement of the pinene ring produced unconjugated diene(1-methyl-4-(propan-2-ylidene) cyclohex-1-ene) (69), which isomerized to γ-terpinene (70). In addition, chloroacetic acid precipitated out of the reaction mixture at 5–7 °C, allowing for easier purification. Chloroacetic acid provided better selectivity and a higher conversion rate than acetic acid, oxalic acid, and hydrochloric acid.

Terpineol (72) can also be prepared from α-pinene in a single cationic rearrangement step using mixtures of inexpensive and common acids. Prakoso^55^ and his group reported the synthesis of terpineol from α-pinene in 53.5% yield using a mixture of phosphoric acid and acetic acid. Binary mixtures of phosphoric acid with formic acid or *p*-toluene sulfonic acids produced lower yields of 72. Interestingly, the addition of phosphoric acid was found to enhance the acidity of acetic acid, which is contrary to the common ion effect. Acidic ionic liquid has been seen as an attractive alternative to traditional organic acids because of higher performance through their cations and better selectivity through their anions. Furthermore, ionic liquids eliminate the need for reaction solvents^56^ and typically offer easier product purification. In addition, the ionic liquids are recyclable and are environmentally friendly. Liu and co-workers reported the use of 1-methyl-3-(3-sulfopropyl)-imidazolium dihydrogen phosphate ([HSO_3_-pmim]H_2_PO_4_) in the synthesis of α-terpineol (72) and its acetate derivative from α-pinene in moderate to high yield.^57^

#### 3.5.1 Synthesis of terpineol from α-pinene by solid-liquid model catalysis

The use of acid impregnated zeolite in terpineol synthesis has been reported by Vital and co-workers in their approach to the synthesis of terpineol from the hydration of α-pinene.^58^ Perhaps, one of the robust syntheses of terpineol from α-pinene using zeolite-acid mixture has been demonstrated by Wijayata and co-workers.^59^ In their approach, trichloroacetic acid was impregnated in zeolite to form TCA/Y-zeolite. This led to a 66% conversion of α-pinene with 55% selectivity to terpineol in 10 min. The conversion rate of pinene increases over time while selectivity for terpineol declines. Kamfene, limonene, and terpinolene were also produced as minor products. The solid–liquid model is superior to the liquid–liquid model, and the latter method is expensive to separate products from reagents. Zeolite is inexpensive and economically viable in industrial-scale synthesis.

#### 3.5.2 Synthesis of terpineol from α-pinene by biological approach

Microorganisms such as fungi and bacteria are excellent sources of enzymes and biologically active compounds. They are relatively inexpensive to culture, and their use of enzymes in the synthesis of biomolecules offers a significant advantage because of the inherent specificity of biosynthetic enzymes. For example, Lee and co-workers reported the biosynthesis of α-terpineol from α-pinene by white-rot fungus *Polyporus brumalis*.^60^

## 4 Pinene in the synthesis of pinene-based ligands

Pinene rigidity, chirality, and unique bicyclic bridgehead provide an excellent pool for chiral ligands. In addition, the presence of olefin and methyl groups allows the design and construction of an endless moiety of ligands tailored for general and specific functions depending on the need.

### 4.1 α-Pinene-based organoborane chiral reagents

**Fig. 5:**
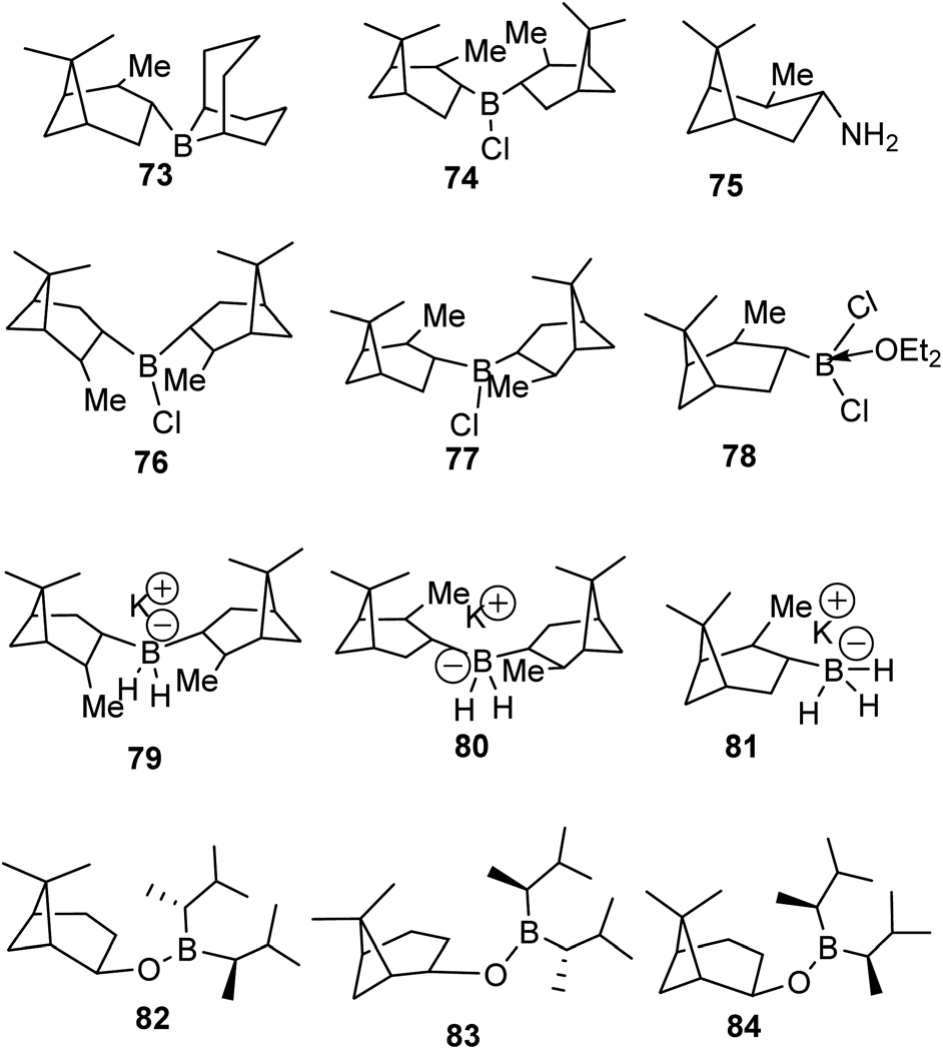
Pinene-based organoborane ligands.

The discovery of the hydroboration reaction in 1956 and subsequent utility in organic transformations has stimulated the design and synthesis of a wide variety of organoborane chiral ligands such as (Alpine-Borane® (73), DIP-Chloride™ (74), amino pinene (75), and pinene based organoboranes (Ipc_2_BR 76–84)) as depicted in Fig. 5 below.^61^

Hydroboration of olefins proceeds in a concerted *syn* 1,2-addition fashion. Therefore, there is no cation or anion intermediate involved, and the addition of two groups occurs on the same face of the olefinic bond. The unique feature of hydroboration is the retention of configuration at the carbon center by the migratory group from boron during the substitution of the leaving group. The last stage in hydroboration follows the S_N^2^_ pathway. Therefore, no intermediate is observed. Unlike a typical S_N^2^_, which results in inversion of configuration, stereochemistry is faithfully preserved in hydroboration. The use of α-pinene-based organoborane chiral reagents accentuates the retention of desired stereochemistry by blocking alternative paths of the migratory group to reach carbon carrying a leaving group.

#### 4.1.1 α-Pinene-based organoborane in the synthesis of homoallylic amine

**Fig. 6:**
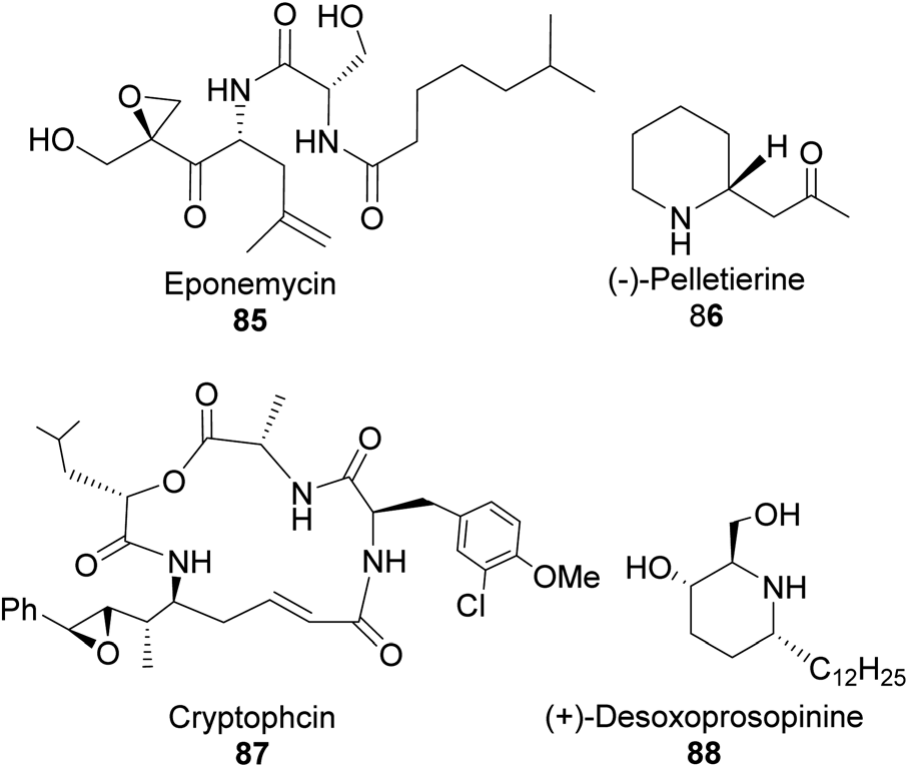
Bioactive natural products-derived from homoallylic amine.

Chiral nitrogen-containing natural products such as linear acyclic and cyclic alkaloid peptides can be synthesized with fewer synthetic steps and high efficiency using homoallylic amines. Though homoallylic amines are not commonly found in natural products, they provide a versatile building block for important pharmacologically valuable molecules such as eponemycin 85,^62,63^ pelletierine 86, cryptophycin 87, and (+)-desoxoprosopinine 89 (Fig. 6).

**Fig. 7:**
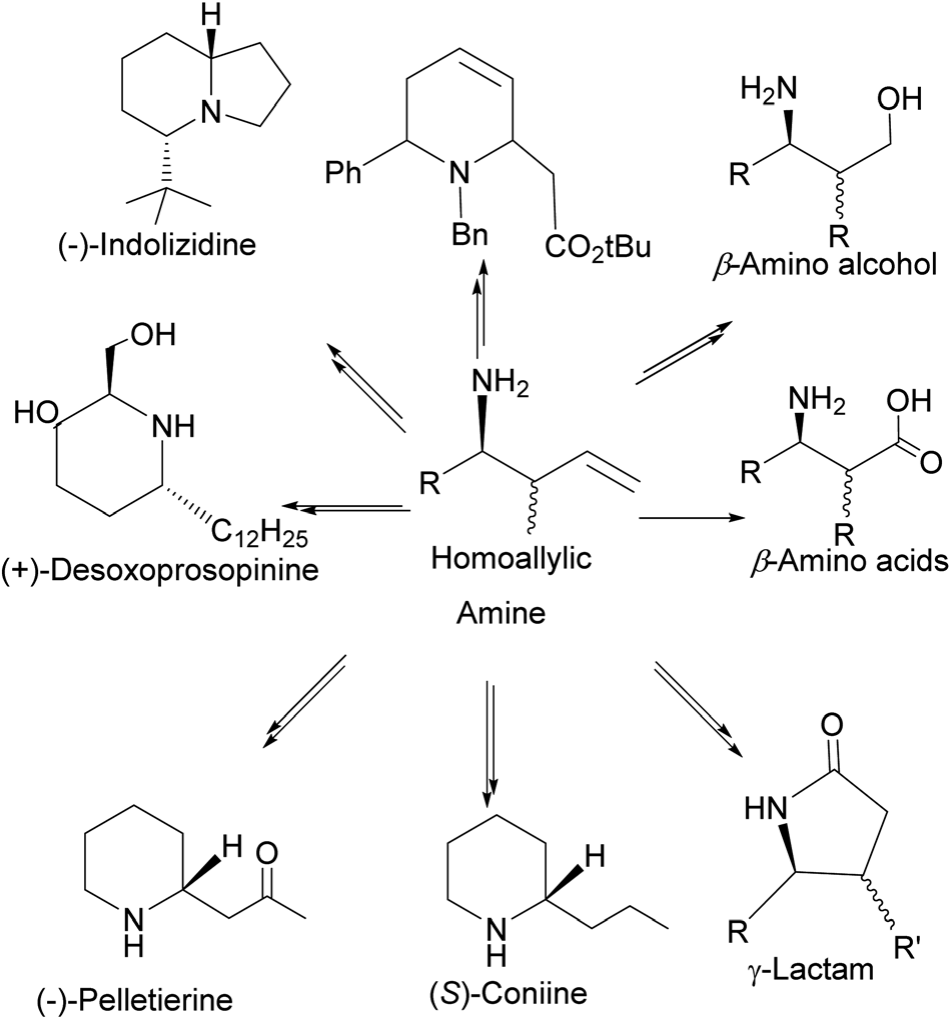
Compounds derived from homoallylic amine.

In addition, homoallylic amines can be useful in preparing important synthetic intermediates such as β-amino acids (essential for the synthesis of β-lactams antibiotics), γ-amino acids, and γ-amino alcohols (Fig. 7).

Therefore, efficient synthetic methods for homoallylic amines and similar molecules are needed. One such method is the allylation of *N*-trimethylsilylbenzaldimine using chiral borane reagents.

##### 4.1.1.1 (−)-β-Pinene-based π-allylpalladium catalyst in allylation of imines

**Scheme 25:**
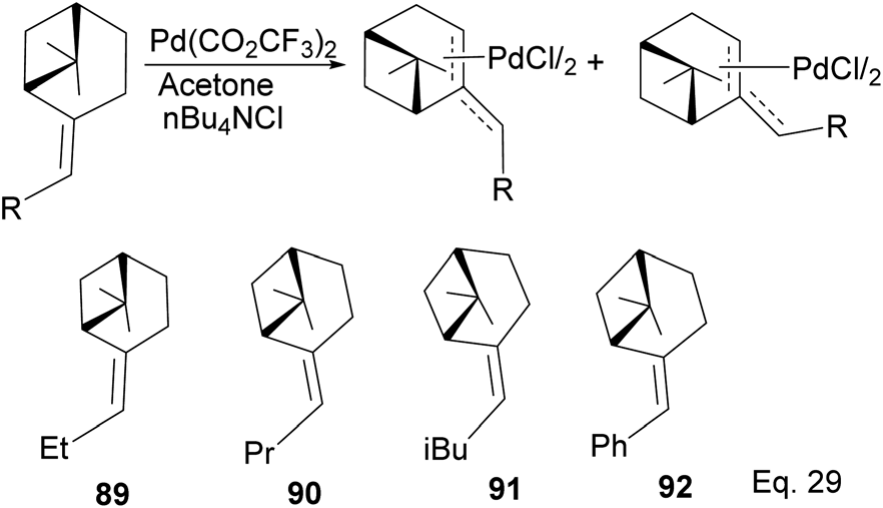
(−)-β-Pinene-based π-allylpalladium catalyst used in allylation of imines.

Homoallylic amines are essential substrates in synthesizing chiral cyclic amine-containing compounds with β-pinene π-allyl palladium framework as catalyst (Scheme 25).

**Scheme 26:**
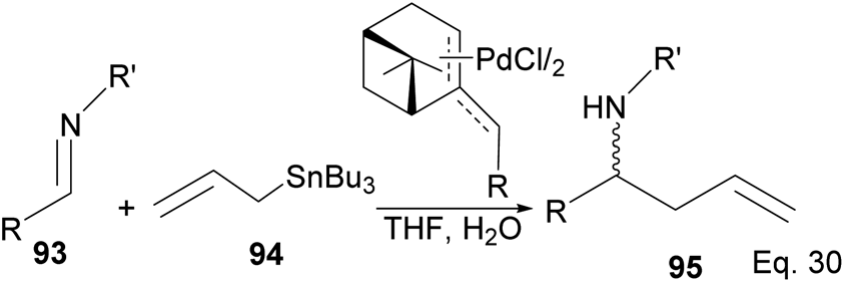
(−)-β-Pinene-based π-allylpalladium assisted enantioselective allylation of imine.

Fernandes and Nallasivam^64^ prepared key homoallylic amine 95 intermediates from enantioselective allylation of imine 93 with organotin 94. Further transformation of amines yielded (*R*)-α-propylpiperonylamine important in the synthesis of human leukocyte elastase inhibitor and (*R*)-pipecolic acid. Enantioselectivity in allylation was slightly enhanced by adding an electron-donating group (EDG) in the imine substrate. *Para*-substituted phenyl groups performed better than *ortho*-substituted phenyl groups. For instance, *para*-substituted phenyl in *R* and *R*′ produced homoallylic amine in 90–98% ee compared with 90% ee (Scheme 26). Adding an electron-withdrawing group (EWG) to the phenyl ring has no impact on the yield but drastically reduced enantioselectivity.

**Fig. 8:**
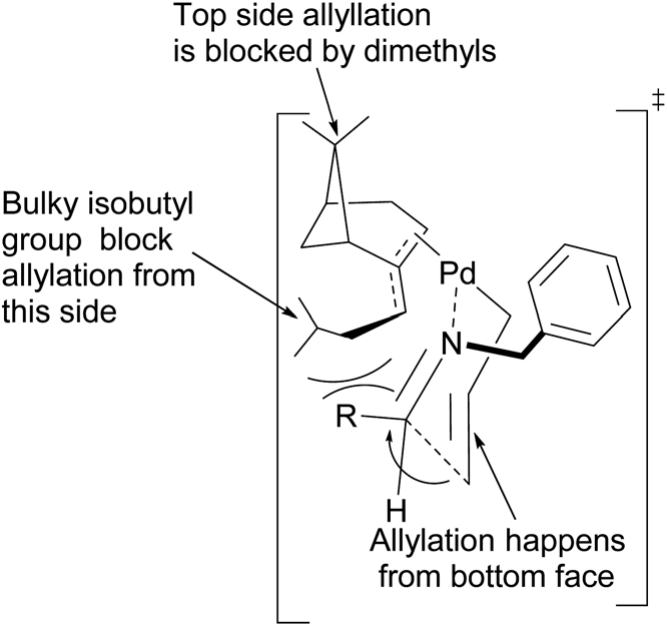
Proposed transition state of (−)-β-pinene-based π-allylpalladium mediated allylation of imine.

Fernandes proposed that the presence of a dimethyl moiety on the cyclobutyl ring of pinene prevents top face allylic transfer. Hence, allylation occurs from the bottom face (Fig. 8).

##### 4.1.1.2 B-Allyldiisopinocampheylborane (Ipc^2^B(allyl)borane) in allyboration

**Scheme 27:**
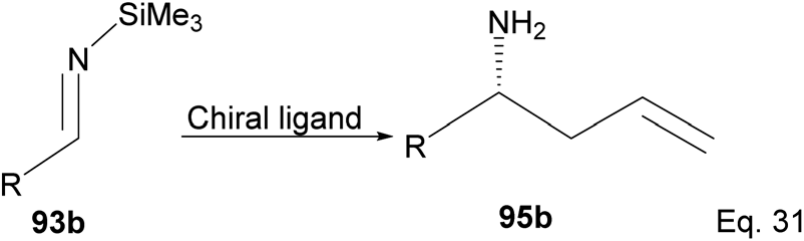
Ipc_2_B(allyl)borane promoted allylation of imine to amine.

Itsuno and co-workers have reported^65^ the allylation of *N*-trimethylsilylbenzaldimine (93b) using different chiral organoborane ligands derived from diols, α-amino alcohols, tartrate ester, and α-pinene to produce homoallylic amine (95b) (Scheme 27).

**Fig. 9:**
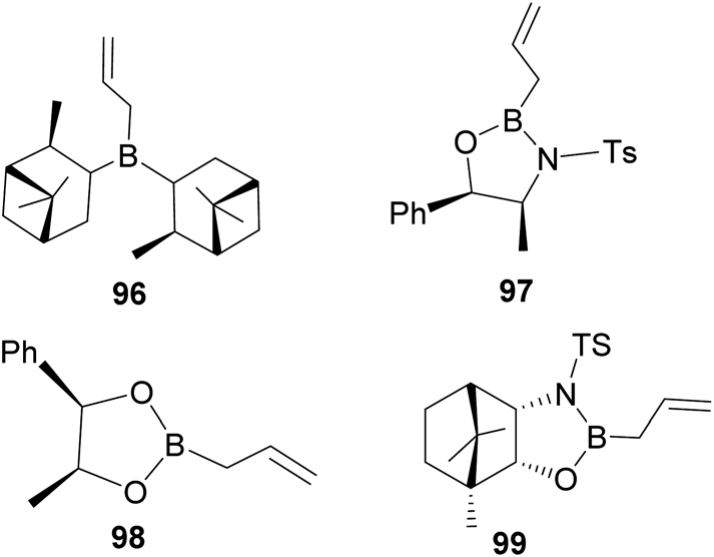
Pinene-derived organoborane used in enantioselective allylation.

In an overall assessment of the work, pinene-based organoborane ligand, *B*-allyldiisopino campheylborane (96, Fig. 9), produced higher enantiomeric excess (ee) than Roush tartrate (98) and camphor-based sulfonyl amino alcohol ligand (99). However, it was inferior to the amino alcohol-derived ligand, norephedrine-derived allyloxazaborilidine (97), which afforded better yield and ee.^66^

**Scheme 28:**
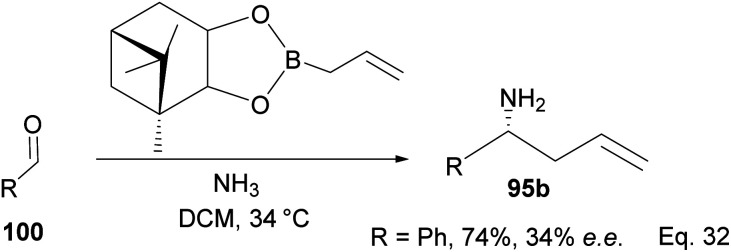
Enantioselective allylation of aldehyde by (−)-*B*-allyldiisopinocampheylborane.

One major drawback of (−)-*B*-allyldiisopinocampheylborane (96) is that it requires a polar environment to achieve high yields. For example, in the three-component allylation of benzaldehyde (100) with Hoffmann's chiral allyboronate^67^ and ammonia by Kobayashi and co-workers,^68^ lower yield (74%) and low enantioselectivity (34% ee) of 1-phenylbut-3-en-1-amine (95b) was obtained in the absence of alcohol (methanol or ethanol) as shown in Scheme 28 below.

**Scheme 29:**
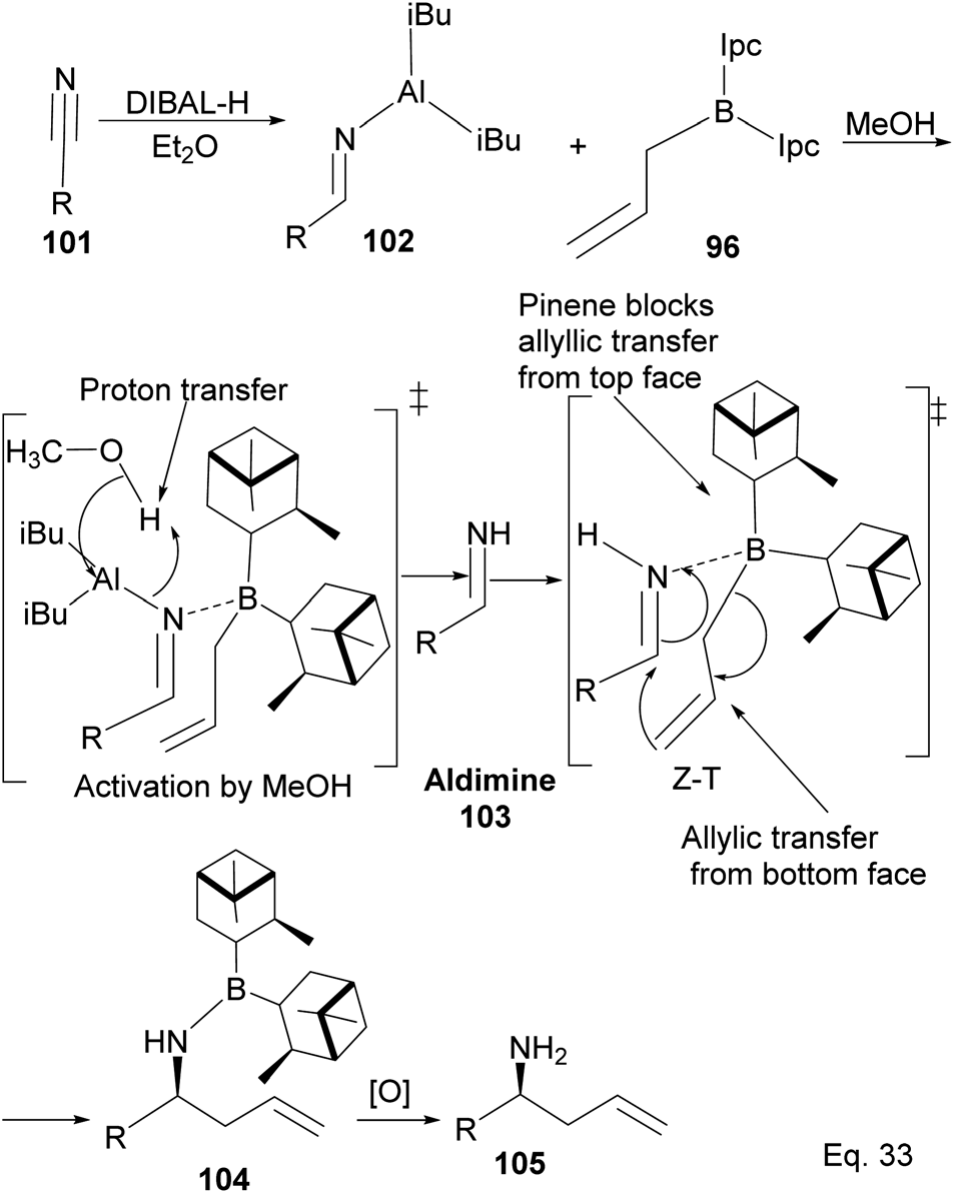
Mechanism of (−)-*B*-allyldiisopinocampheylborane-mediated enantioselective allylation of aldimine to homoallylic amine.

**Fig. 10:**
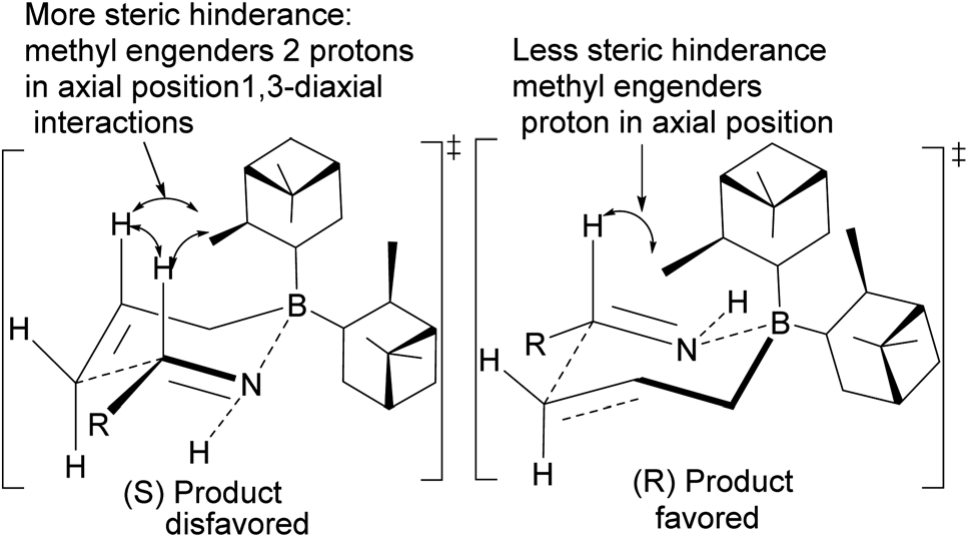
Proposed transition states in the allylation of aldimine by (−)-*B*-allyldiisopinocampheylborane.

Allylation by substrate activation with polar reagents such as MeOH or H_2_O is not limited to carbonyl-containing groups. Nitriles can be used and are excellent substrates because of the nitrogen atom. For example, reducing nitrile^69^101 to *N*-aluminoimine 102 can serve as a precursor for proton activation. Spectroscopic evidence from ^11^B-NMR spectroscopy supported the mechanistic role of protic solvents in the reaction. It was found that in the presence of water or alcohol, aldimine 103 is formed through the protonation of the nitrogen atom. Once formed, the aldimine coordinates with the organoborane forming a Zimmermann–Traxler (Z–T) six-membered ring transition state (Scheme 29), and the homoallylic amine 105 is produced from the oxidation of amine-Ipc adduct 104. Enantioselectivity is determined by steric interaction between proton at prochiral aldimine center and α-pinene ring. In the favored (*S*) configuration (Fig. 10), only one axial proton from prochiral aldimine carbon encounters steric interaction with α-pinene ring protons. In the disfavored (*R*) configuration, the axial imine and protons on the prochiral carbon encounter pinene protons.

**Scheme 30:**
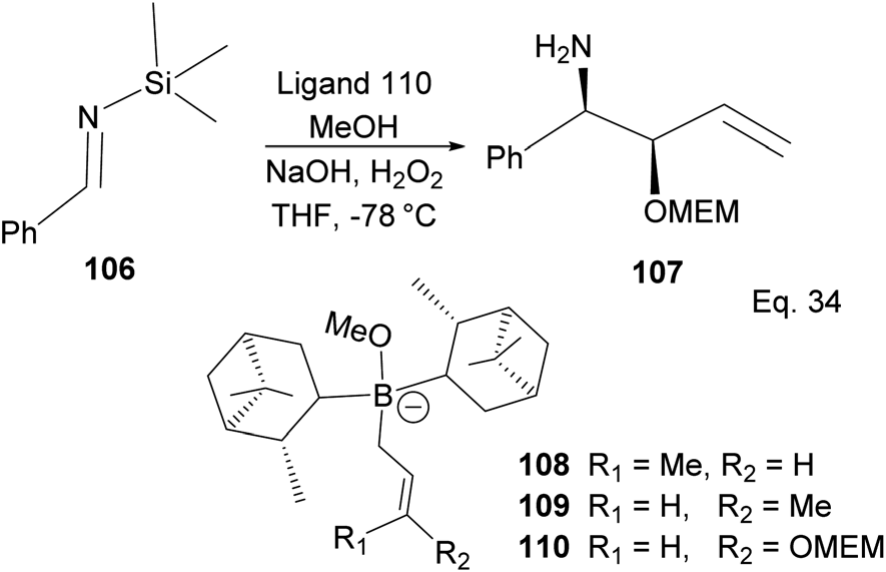
Allyboration and crotylallylaboration of silylaldimine.

A similar mechanism happens in the allyboration of *N*-aluminoimine and in the crotylboration and alkoxyallylboration of imines using α-pinene derivatives, as shown in Scheme 30. For example, the α-pinene-containing boronate complex 110 was used in the crotylallylboration and alkoxyallylboration of aldimines 106 (Scheme 30) to produce ether-protected amino alcohol 107 with 65% yield, 9.5% ee, and 98% de.

**Scheme 31:**
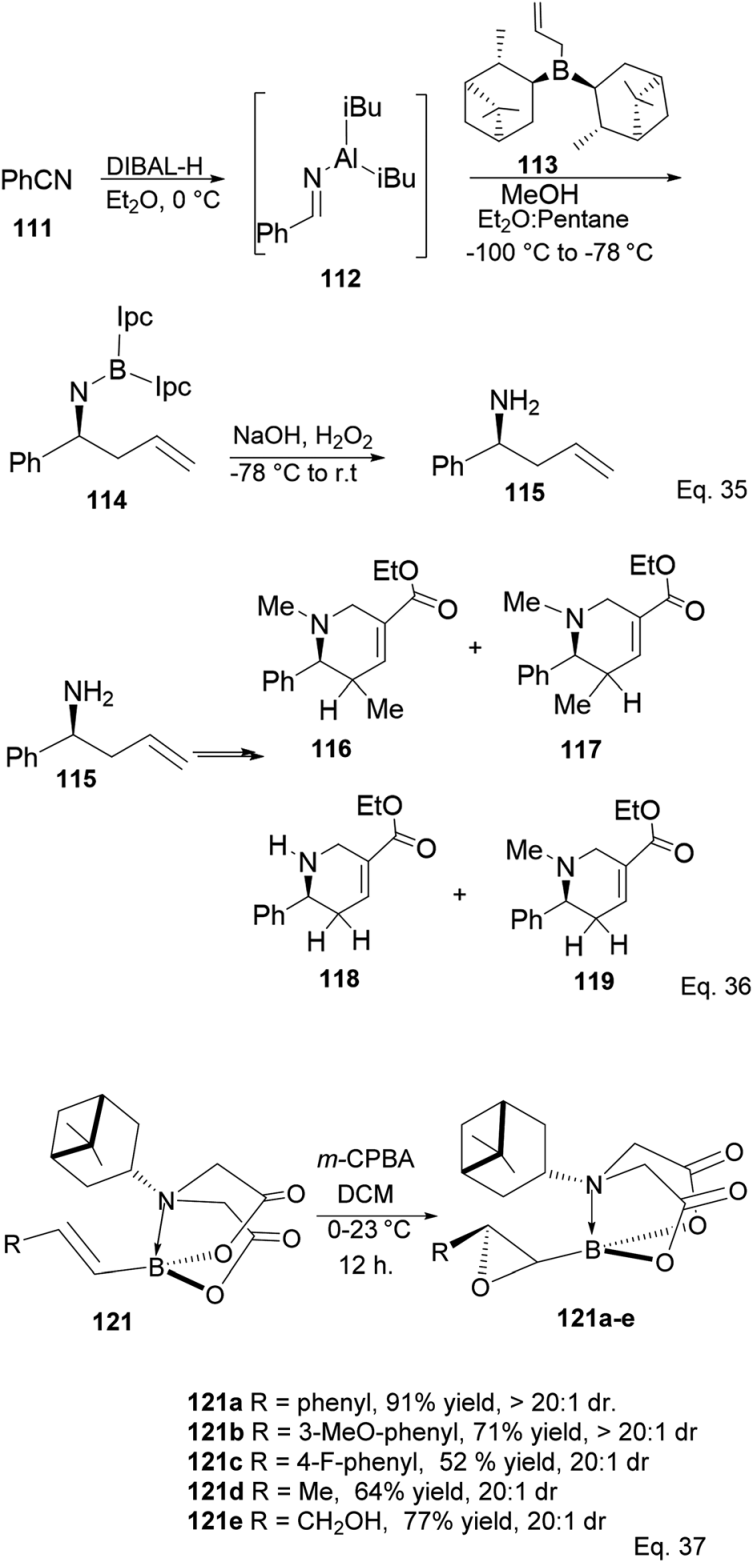
Derivatives of GABA uptake inhibitors containing the tetrahydropyridine motif (Eq. 36) and PIDA-promoted enantioselective epoxidation (Eq. 37).

Ramachandran and colleagues reported successfully prepared ester derivatives (116 in 75%, 117 in 70%, 118 in 72%, and 119 in 85% yields, Scheme 31a) of GABA uptake inhibitors (nipeocotic acid and guvacine)^70^ containing tetrahydropyridine scaffold from allylboration of *N*-aluminoimines using α-pinene-based organoborane.

**Fig. 11:**
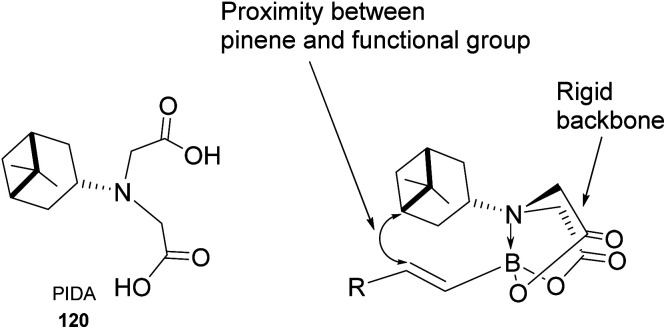
Pinene-derived iminodiacetic acid (PIDA).

Pinene-derived iminodiacetic acid (PIDA)^71^ ligand 120, shown in Fig. 11 prepared by Burke and co-workers, provides a robust and versatile C(sp^3^) boronate. Its ability to induce chirality derives from the proximity of the functional group to the pinene, conjugated to the rigid iminodiacetic boronate backbone, during transformation.

The ligand is tolerant to various functional groups in the substrate. Therefore, once the desired stereogenic center has been introduced, the boronate functional group can be removed through a metal-catalyzed coupling reaction.

**Scheme 32:**
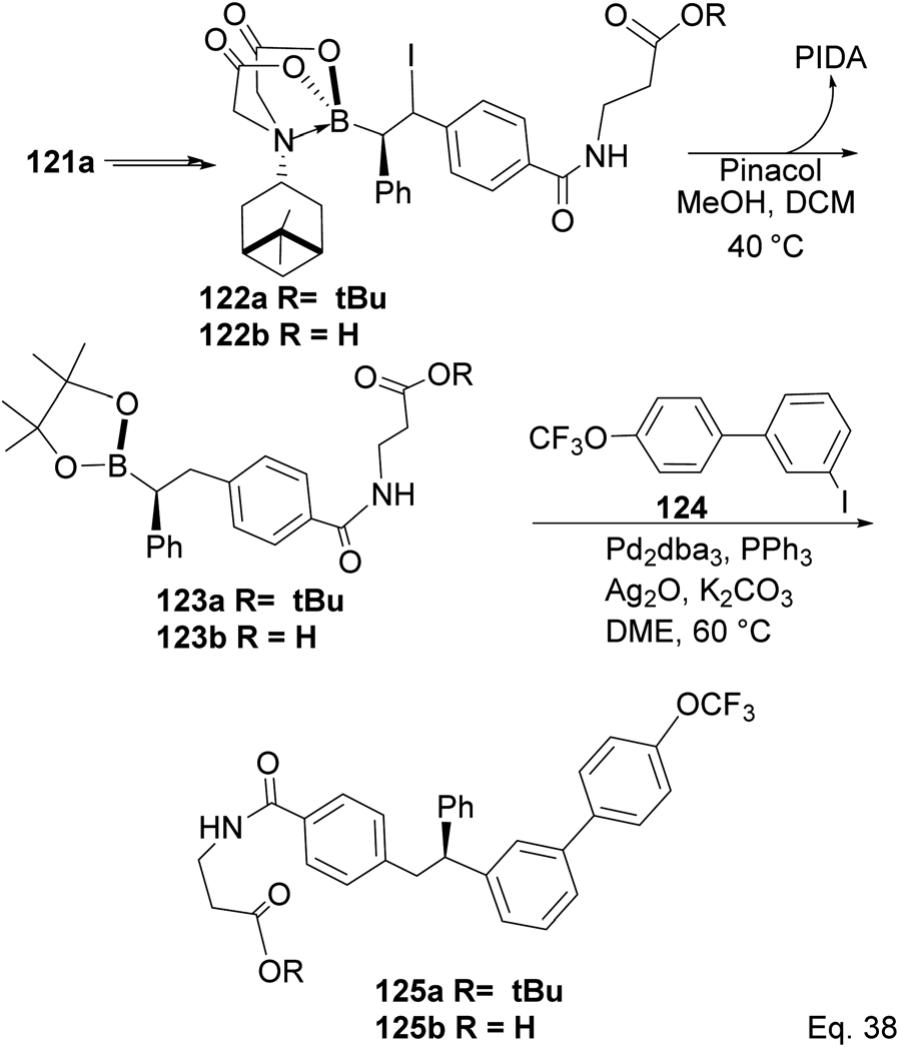
PIDA in the synthesis of glucagon receptor antagonist.

Compound 121a obtained from the epoxidation of boronate adduct 121 (Scheme 32) was used to synthesize a glucagon receptor antagonist 125b, with potential use in treating type II diabetes.

### 4.2 α-Pinene chiral auxiliary in enantioselective synthesis

**Fig. 12:**
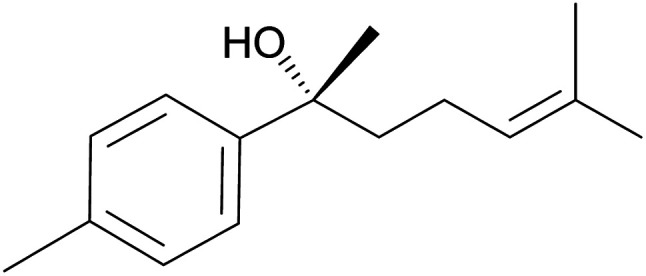
(+)-(*R*)-Gossonorol.

Pinene-based amino alcohol chiral auxiliaries were used to synthesize secondary alcohols from aldehydes and diethyl zinc in enantioselective synthesis. Secondary alcohols are key intermediates in the synthesis of natural products such as (+)-(*R*)-gossonorol (Fig. 12)^72,73^ which was studied for its antifungal and antitumor activities.

**Scheme 33:**
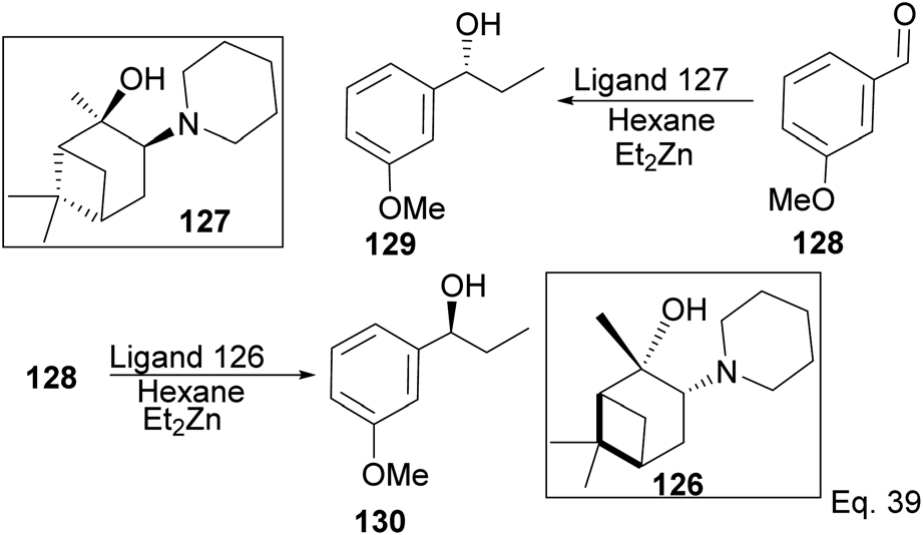
Pinene-based chiral auxiliaries in enantioselective alkylation of aldehyde.

Traditionally, secondary alcohols are made from Grignard reagents or organolithium compounds, but the high reactivity and fastidious conditions required come at a cost in stereoselectivity control. Diethyl zinc and α-pinene chiral auxiliaries offer a good alternative in the enantioselective synthesis of secondary alcohols, as shown by Marques and co-workers. Marques used ligand 126 to synthesize (1*R*)-1-(3-methoxyphenyl)-1-propanol alcohol 130 in 96% yield and 92% ee. Ligand (127) prepared from (+)-pinene produced (1*S*)-1-(3-methoxyphenyl)-1-propanol 129 in 85% yield with 91% ee (Scheme 33).

**Scheme 34:**
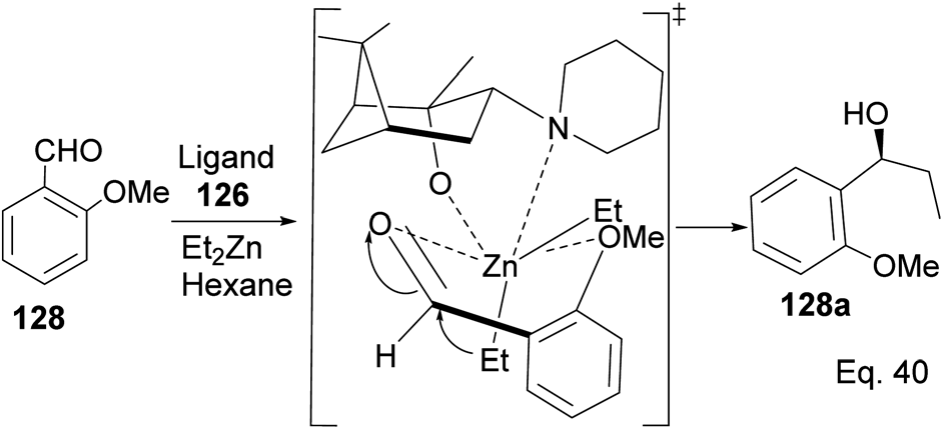
The transition state in the alkylation of aldehyde 128 mediated by pinene amino alcohol.

Marques and co-workers employed four synthetic steps to prepare the chiral amine auxiliaries containing an α-pinene moiety.^74^ Ligand 126 showed higher enantioselectivity because of its effectiveness in inducing steric hindrance to the incoming nucleophile (Et_2_Zn). The presence of cyclic amine and bridgehead methyls increases hindrance to alkylation of aldehyde. The approaching diethyl zinc delivered ethyl group from the bottom where it is less hindered, as shown in Scheme 34 below.

## 5 Pinene in the synthesis of polymers

**Fig. 13:**
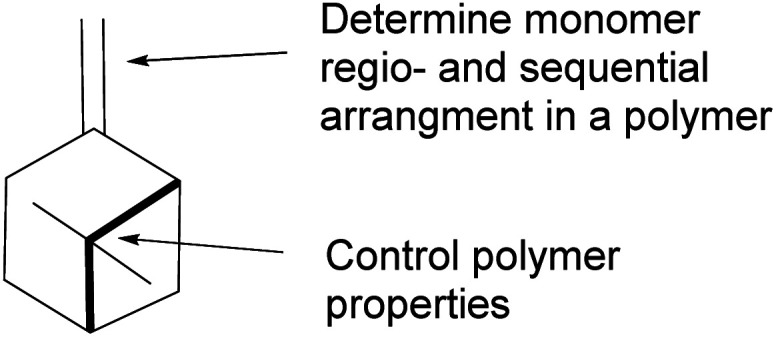
δ-*Pinene* structure.

Pinene olefinic functional group and its chiral bicyclic bridgehead offer an attractive choice as a monomer in polymer synthesis. Olefin functionalization in polymer synthesis can be carried out using a variety of mechanistic pathways such as free radical, cationic polymerization, and metal-mediated metathesis. Furthermore, polymer properties, including thermal and optical activities, can be controlled by manipulating the bridgehead of the stereogenic centers (Fig. 13) and the two prochiral olefinic carbon centers depending on the mechanism of polymerization.

**Scheme 35:**
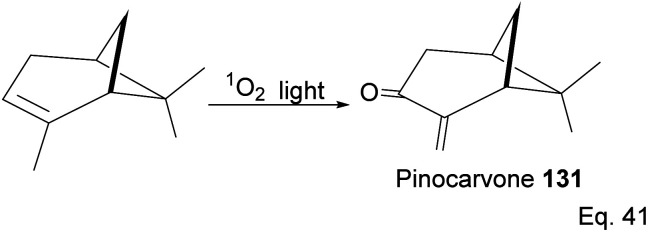
Light-catalyzed oxygenation of pinene to pinocarvone.

Pinene isomers provide an excellent substrate for fashioning and tailoring the desired monomer to synthesize specific polymers depending on the intended use. For instance, (+)-pinocarvone (Scheme 35) is the monomer for synthesizing polyketone polymers, as discussed in radical polymerization in the next section.

Polymers derived from pinene scaffold are of economic importance. β-Pinene-derived polymers have been used as resins for many materials, including adhesives and rubber additives. In addition, because of the inert nature of pinene polymers, it is used in food packaging. Improving synthetic methods through better catalysts and activators to increase yield and desirable polymer properties has been an ongoing research endeavor. Lewis acids such as AlCl_3_, SbCl_3_, and transition metal salts have been used in polymer and copolymer synthesis from different monomers.

### 5.1 Synthesis of polyketone polymer from α-pinene-derived pinocarvone

**Scheme 36:**
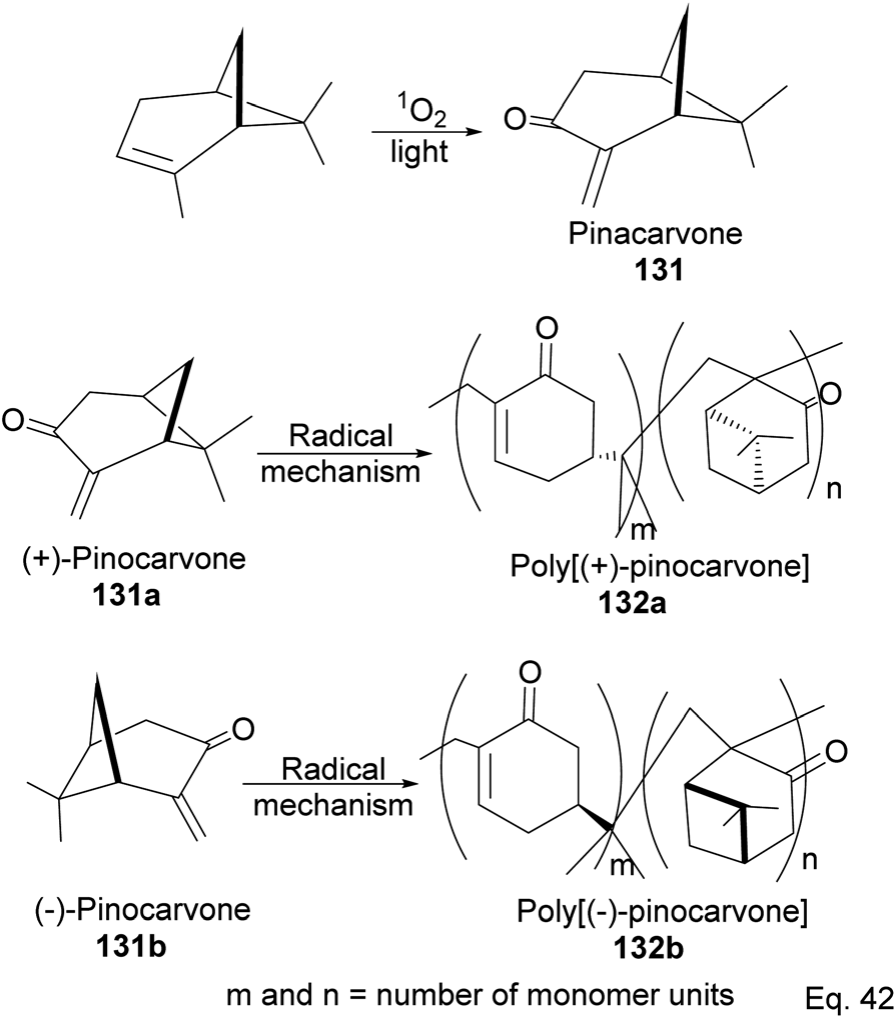
Radical polymerization of pinacarvone.

α-Pinene can be transformed into the conjugated *exo*-olefinic α,β-unsaturated ketone, (+)-pinocarvone, through photooxidation using singlet oxygen (^1^O_2_) in the presence of tetraphenyl porphyrin as photosensitizer (Scheme 36). Polymerization through pinocarvone offers an advantage over α-pinene because its *exo*-olefinic group is more accessible. Pinocarvone also has an advantage over β-pinene because of its enhanced high reactivity resulting from the conjugation of its *exo*-olefinic group with the ketone functional group. Polyketones 132a and 132b were prepared by photooxidation of (+)-pinocarvone under bulk conditions at 60 °C with azobisisobutyronitrile (AIBN) initiator in fluorinated solvents. Both polyketones are optically active due to chirality transfer from the cyclohexyl ring of (+)-pinocarvone.

**Scheme 37:**
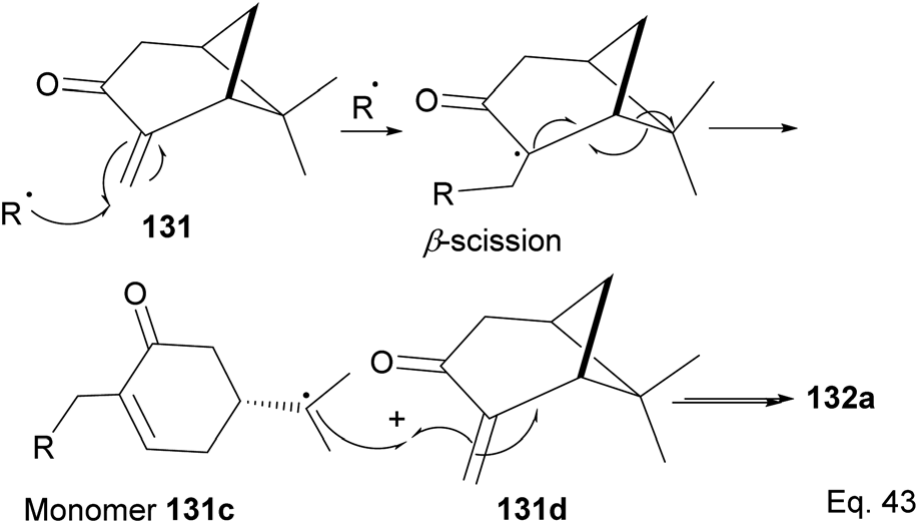
Mechanism steps in the radical polymerization of pinacarvone.

The polymer is composed of an α,β-unsaturated cyclohexanone moiety 131c (>90%) and a pinanone moiety 132d formed during the initial stages of the *exo*-olefin-enabled diradical formation (Scheme 37). This is consistent with the radical mechanism, which favors the most stable 3° radical intermediate formed through β-scission of (+)-pinocarvone.

**Scheme 38:**
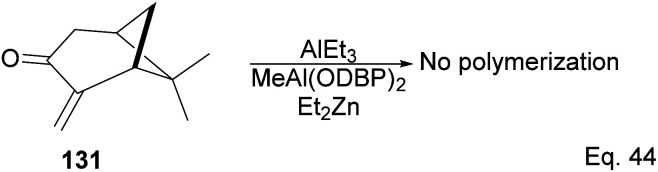
Lewis acid (AlEt_3_)-assisted polymerization of pinacarvone.

Interestingly, an attempt to polymerize (+)-pinocarvone *via* anionic ring-opening failed even in the presence of *tert*-butyllithium, or the addition of aluminum additives such as triethylaluminium (AlEt_3_) and *bis*(2,6-di-*t*-butylphenoxy)methylaluminum (MeAl(ODBP)_2_) with *tert*-butyllithium or with Et_2_Zn (Scheme 38).

### 5.2 Synthesis of ε-lactams derived from β-pinene

**Scheme 39:**
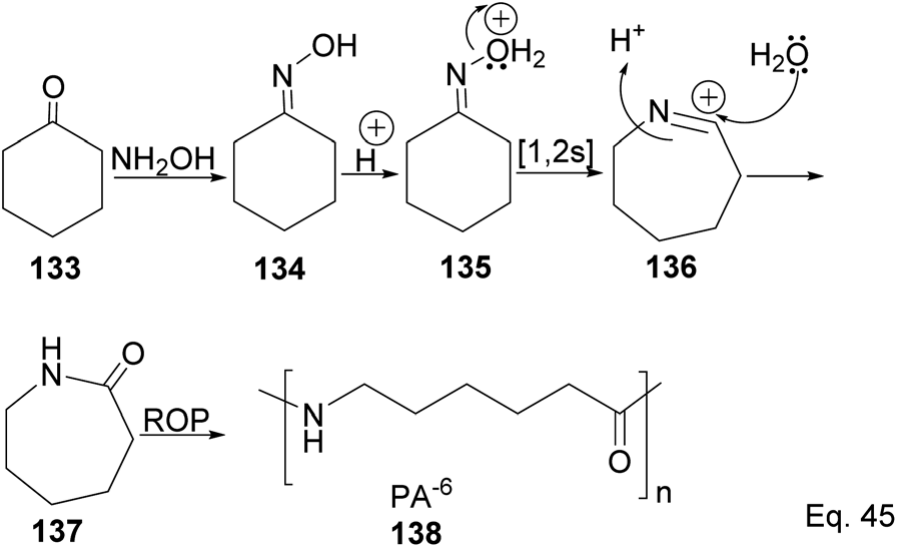
Polymerization of caprolactam derived from the rearrangement of cyclohexanone oxime.

Caprolactam is an important industrial chemical with a wide range of uses, including in the production of nylon-6 filaments, fibers, and plastics. ε-Lactams are prepared from acid-catalyzed Beckmann rearrangement of the oxime (134) derived from the condensation of cyclohexanone (133) and hydroxylamine (Scheme 39). The ring-opening polymerization of ε-lactams 137 produces nylon-6 polymers (138).

**Scheme 40:**
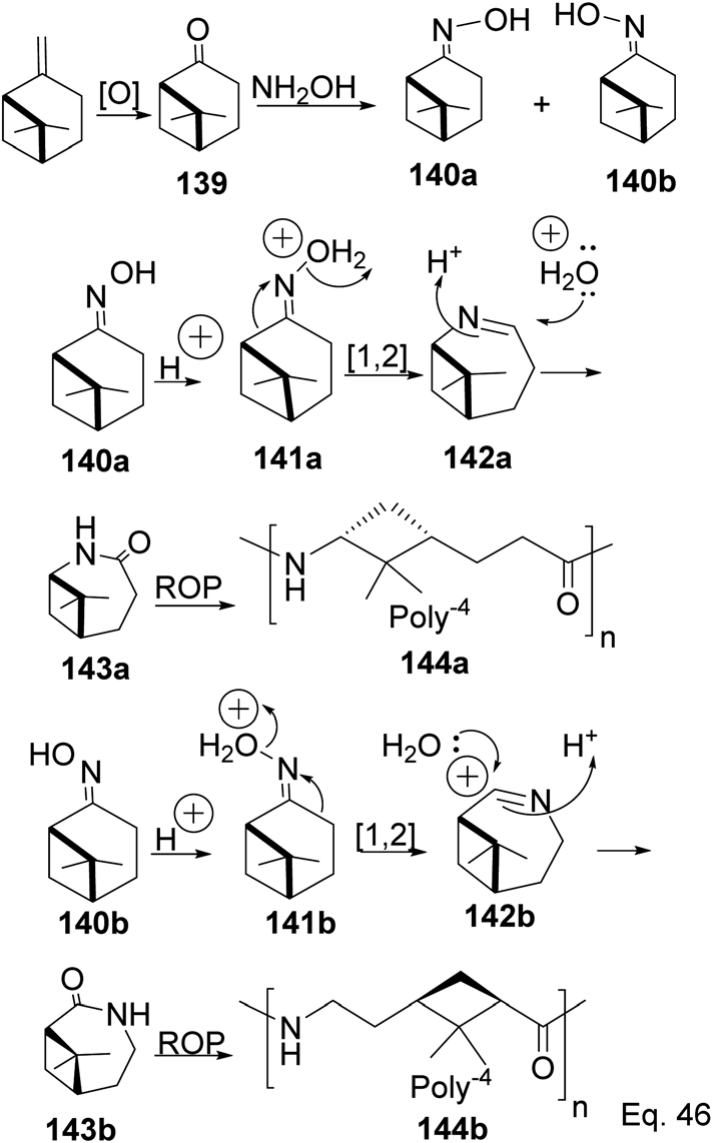
Polymerization of poly(ε-lactams) *via* cationic Beckmann rearrangement.

The major drawback of using cyclohexanone as the precursor for ε-lactams is the monomer's lack of a chiral center. The absence of a chiral center affects the polymer's photo-and thermostability, durability, and rigidity, which are important for most polymer applications. Therefore, replacing cyclohexanone with β-pinene as the starting material provides a cheap and sustainable alternative. Winnacker and co-workers reported the synthesis of poly (ε-lactams) 144a–b from β-pinene (Scheme 40) in ring-opening polymerization of ε-lactams (143a and 143b).^75^

**Scheme 41:**
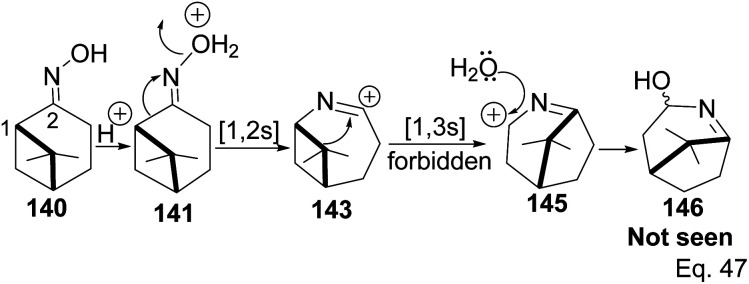
Forbidden [1.3s] cyclobutyl alkyl shift in 7,7-dimethyl-2-azabicyclo[4.1.1]oct-2-yn-2-ium.

Chirality in β-pinene is faithfully preserved in the oxime. Interestingly, Beckmann's ring expansion by 1,2-sigmatropic migration of alkyl at C-2 to nitrogen was not followed by bridgehead C-1 ring expansion to C-2 alkenyl cation 143 (Scheme 41). In Beckmann's rearrangement, the migratory and leaving groups must be anti-periplanar.

Furthermore, migratory aptitude depends on the stability of carbocation. In addition, the 1,2-migration is suprafacial *and allowed*. In this case, migratory alkyl C-1 in 140 must traverse nitrogen heteroatom to reach alkenyl carbocation 143. Thus, any bridgehead ring expansion would have to follow *antrafacial*-1,3-σ-migration (not allowed in cationic rearrangement) to produce 146.

#### 5.2.1 Cationic polymerization of pinene

**Scheme 42:**
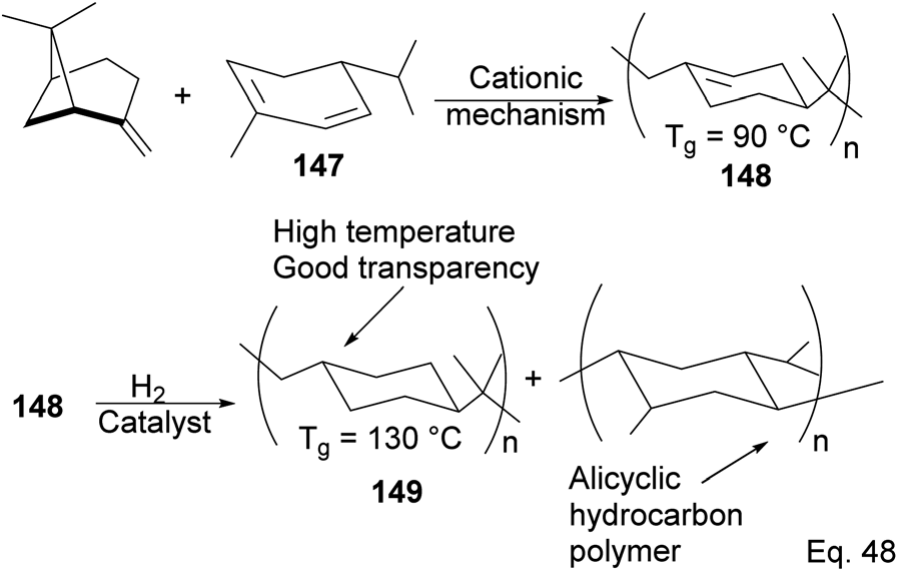
Polymerization of (−)-α-phellandrene and (−)-β-pinene.

As an example of the cationic synthesis of poly(β-pinene), Kamigato and co-workers reported the synthesis of a polymer with a high molecular weight and high glass temperature *T*_g_ = 130 °C (after hydrogenation) from a mixture of (−)-β-pinene and (−)-α-phellandrene 147.^76^ Hydrogenation of the polymer's cyclohexenyl backbone dramatically improved its thermoresistance (*T*_g_ = 90 °C in unsaturated polymer 148 and *T*_g_ = 130 °C in hydrogenated polymer 149). In addition, hydrogenated polymer 145 is durable and resistant to thermal destruction, with only a 10% degradation at a temperature > 400 °C (Scheme 42).

#### 5.2.2 Binary Lewis acids (SbCl_3_/AlCl_3_)-mediated polymerization of α-pinene

**Scheme 43:**
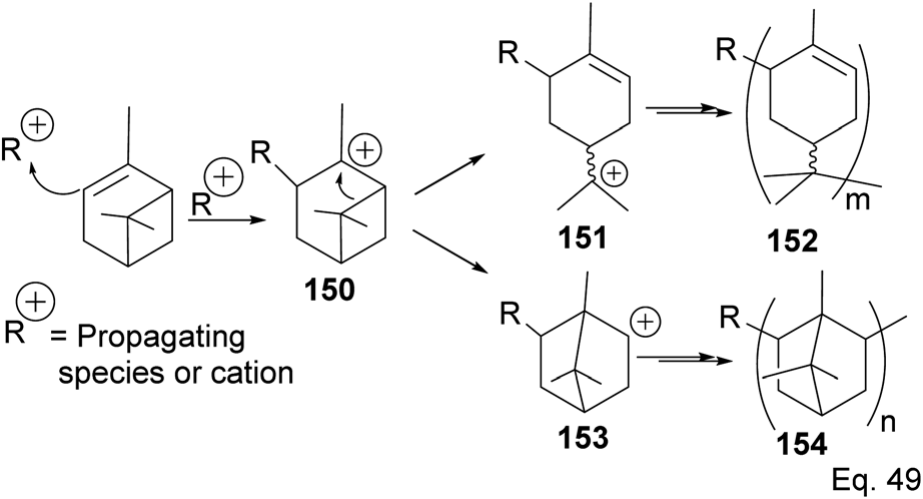
Cationic polymerization of δ-pinene mediated by (SbCl_3_/AlCl_3_).

Cationic polymerization of α-pinene catalyzed by AlCl_3_ occurs *via* rearrangement isomerization of its cyclohexenyl ring. There are two paths for the rearrangement, starting with carbocation 150 (Scheme 43). First, *via* the disintegration of cyclobutyl ring to relieve angle strain in 3° carbocation 151, and second, *via* a 1,2-Meir-Wiegner alkyl shift ring expansion of norbornane-type 2° cation 153 from cyclobutyl to cyclopentyl ring skeleton to relieve angle strain. In both cases, the formation of 152 and 154 proceeds from the nucleophilic attack of another pinene molecule on carbocation 151 or 153.

**Scheme 44:**
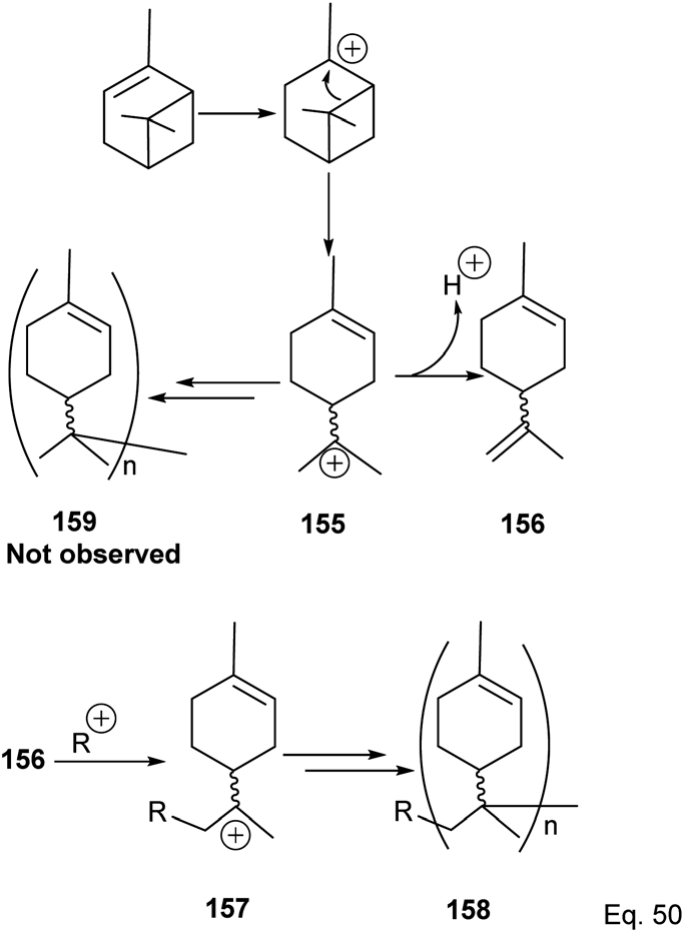
Metal-based Lewis acid promoted cationic polymerization of α-pinene.

Deng and co-workers have shown that the polymerization of α-pinene in the presence of AlBr_3_, EtAlCl_2_, or AlCl_3_ alone tends to favor the norbornane cation pathway. In contrast, the addition of SbCl_3_ to AlCl_3_ favored the terpenyl carbocation 151.^77^ The SbCl_3_/AlCl_3_ (in 0.50 ratio and at −15 °C) cationic polymerization of α-pinene produced oligomer 152 (>90% yield) in relatively high molecular weight (*M*_n_ = 1140 and MW = 2590).^78^ Using only AlCl_3_ for polymerization produced dimers and low MW oligomers. However, the addition of SbCl_3_ rapidly increased the yield and enabled the production of oligomers with higher MW, whereas SbCl_3_ alone did not have any catalytic activities. At Sb/Al ratio > 0.50, the MW, *M*_n,_ and numbers of dimers formed were not dependent on the catalytic activities of the mixture. ^1^H-NMR of polymers formed from Sb/Al mixtures revealed the presence of *endo*-olefinic and single germinal methyl protons at 5–6 ppm and 0.7–1.00 ppm, respectively from 158, and the absence of *exo*-olefinic proton signals. The spectroscopic evidence strongly suggests that the cationic polymerization occurs through the terpeniol cation 157 instead of limonene carbocation 155 (Scheme 44).

**Scheme 45:**
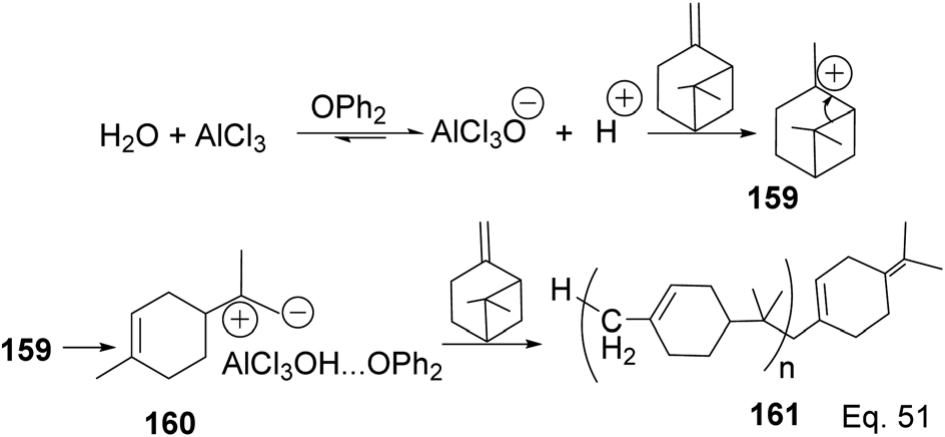
AlCl_3_-catalyzed polymerization of β-pinene.

In contrast to the polymerization of α-pinene, the polymerization of β-pinene using AlCl_3_ as the only catalyst happened very rapidly and produced polymers of relatively high molecular weight (*M*_n_ approximately 2000), but the addition of SbCl_3_ poisoned the catalyst and led to poor catalytic activity. An attempt to produce copolymers of both pinene isomers with SbCl_3_/AlCl_3_ catalyst and 2,6-di-*tert*-butyl-4-methylpyridine (DTBMP) as initiator failed and resulted in homopolymerization of individual monomers.^79^ In fact, no small MW oligomers were observed in the polymerization of β-pinene, which implies that the two species have remarkable reactivity, but β-pinene is more reactive than α-pinene. The utility of AlCl_3_ as a catalyst in cationic polymerization has been well studied for practical industrial application. For instance, Kennedy and co-workers^80^ reported the synthesis of poly(β-pinene) 161 with *M*_n_ = 40 000 and *T*_g_ of 65 °C, at relatively low temperatures (−23–100 °C) using EtAlCl_2_ as catalyst and H_2_O as co-initiator. Kostjuk and co-workers also reported the synthesis of thermally stable (*T*_g_ = 82–87 °C) and high molecular weight poly(β-pinene) in AlCl_3_ catalyzed reaction (Scheme 45).^81^ Kostjuk's synthetic approach is economically feasible for industrial production since it requires low temperature, low concentration of both H_2_O/AlCl_3_OPh_2_ initiator and AlCl_3_ (2.5–5.5 mM), and dilute monomer solution. High β-pinene monomer concentration (0.55 M) is tolerable without sacrificing polymer molecular weight *M*_n_.

### 5.3 Transition metal-mediated polymerization of β-pinene

**Scheme 46:**
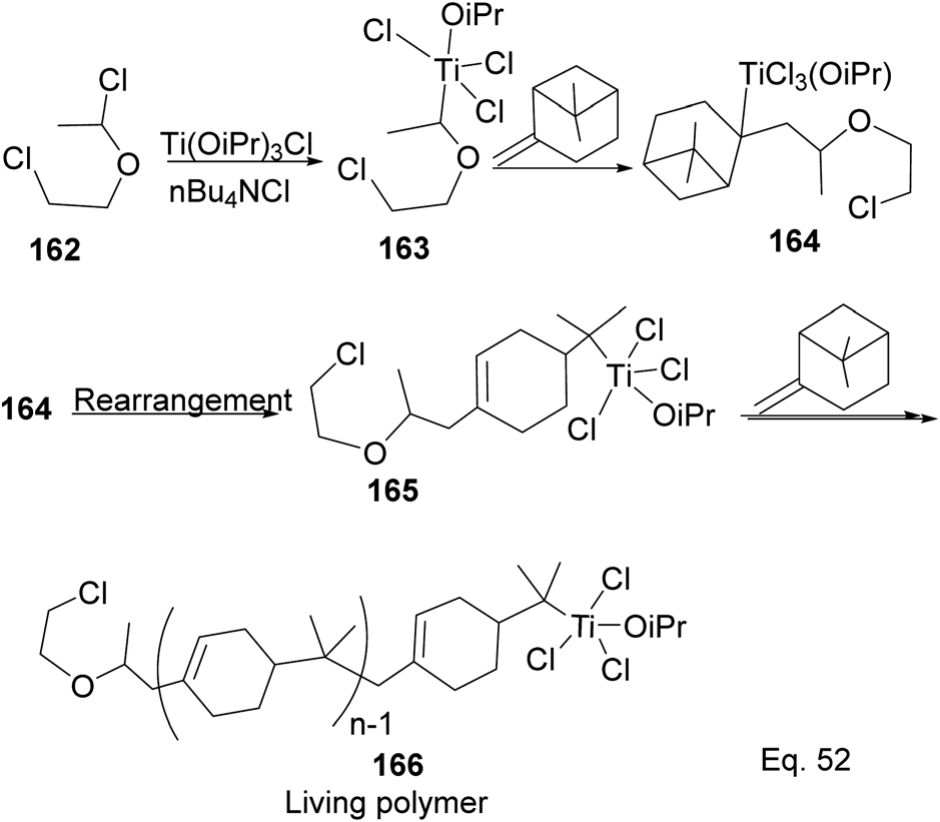
Ti(iOPr)Cl_3_-promoted cationic polymerization of β-pinene.

**Scheme 47:**
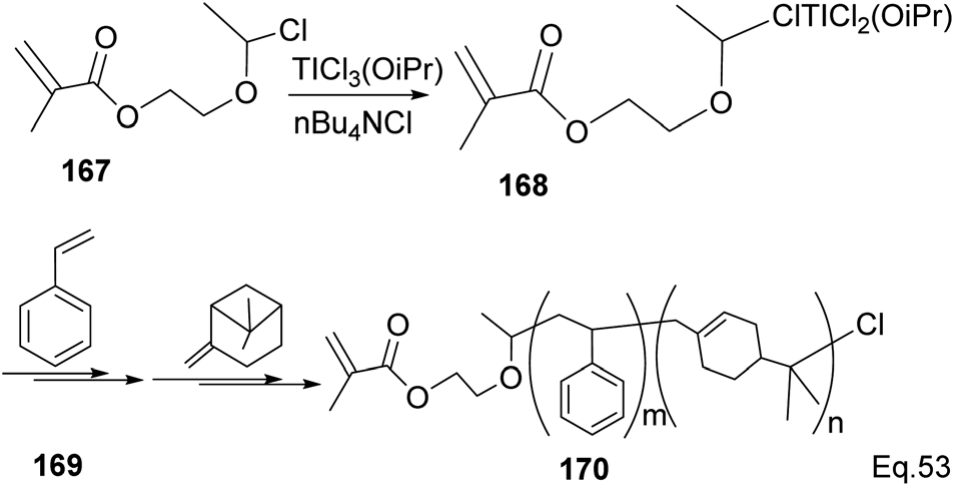
Ti (iOPr)Cl_3_-promoted cationic copolymerization of β-pinene and styrene.

Transition metal complexes-mediated polymer synthesis provides an efficient synthesis of desirable polymer properties. Transition metal catalysis is less prone to side reactions commonly seen in the cationic rearrangement of the strained ring because ligand around the metal center guides monomer during incorporation to the polymer chain while maintaining the desired configuration in the polymeric chain. Early transition metals such as Nb, Ti, Zr, and Hf have become workhorses in polymer and copolymer synthesis. The metals have higher oxidative states, higher oxygen affinity, lower electronegativity tendencies, and allow the introduction of polar functional groups. Those features make the custom design of polymers to improve desirable features such as polydispersity, rheology, density, and control of molecular architecture (*regio*-and stereochemistry) in polymeric chains possible.^82^ Lu and co-workers reported low temperature (40 °C) synthesis of α end-functionalized polymers and macromonomers from β-pinene using TiCl_3_(OiPr) as the catalyst and a mixture of HCl_(aq)_ and CH_3_CH(OCH_2_CH_2_Cl)Cl as the initiator in the cationic polymerization reaction (Scheme 46).^83^ Furthermore, Lu uses the same catalyst and initiator in radical copolymerization reaction to prepare graft copolymers from polar methyl methacrylate (MMA) and graft chain β-pinene (Scheme 47).

#### 5.3.1 Late transition metals in polymerization of β-pinene

**Fig. 14:**
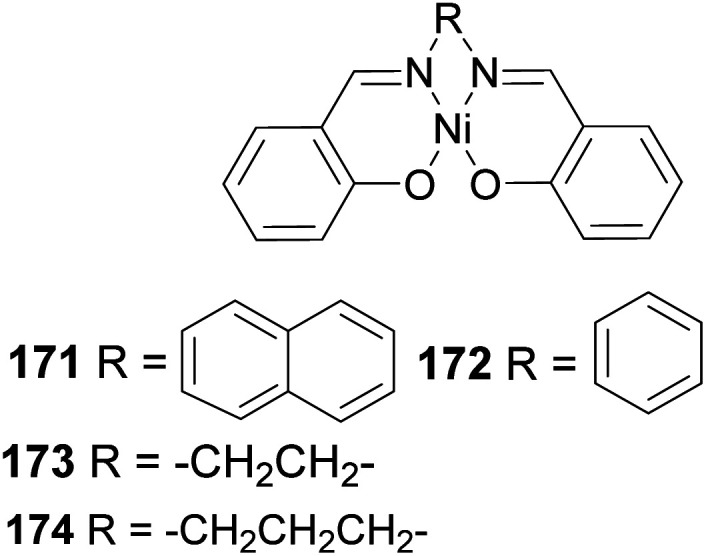
Diimine nickel complex used in polymer synthesis.

Polymerization mediated by late transition metals such as nickel and palladium^84^ has an inherent advantage of lower acidity, air and thermostability, functional group, and solvent tolerance because of less affinity to oxygen. For example, the diimine nickel complex in Fig. 14 has been used as a catalyst in polymer synthesis.

**Scheme 48:**
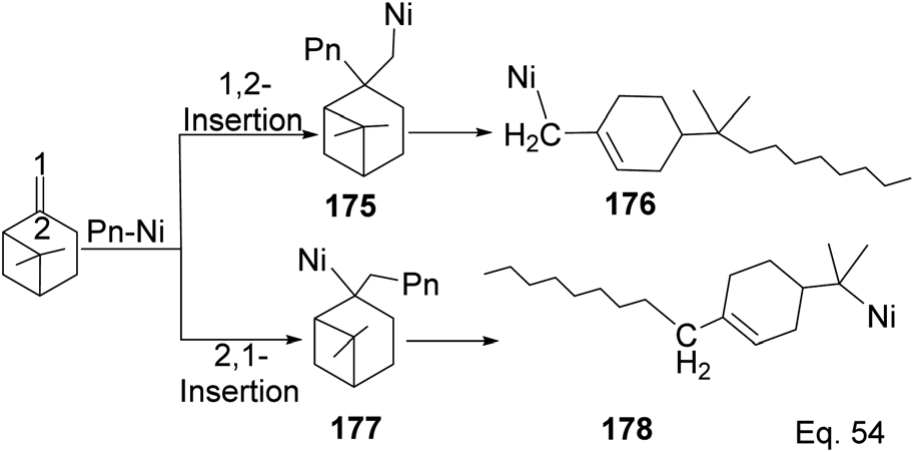
Nickel-mediated ring opening of β-pinene.

Yu and co-workers^85^ reported using Schiff-base nickel complex catalyst in synthesizing high molecular weight poly(β-pinene) (*M*_n_ = 10 900) with a high yield of 1.25 × 10^7^ g poly-β-pinene/mol of Ni at 40 °C. Methylaluminoxane (MAO) was used to activate the nickel complex catalyst at Al/Ni mole ratio = 500. Activated nickel inserts itself in the pinene double bond in 1,2-fashion, which allows a nickel chain transfer from catalyst to the tertiary carbon center (C-2) in the monomer (Scheme 48). Regeneration of nickel catalyst occurs *via* proteolysis of Ni–C bond at the polymer head.

**Scheme 49:**
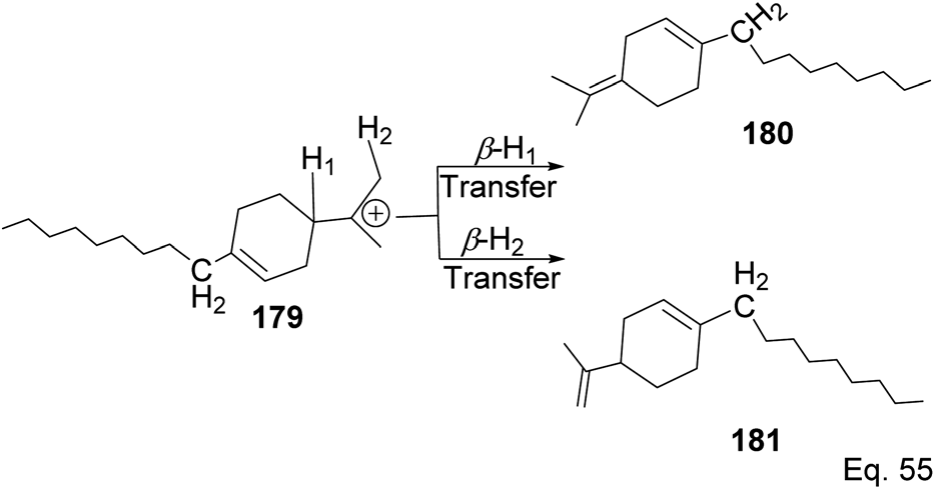
Chain termination pathways *via* β-hydride transfer from carbon to nickel complex.

Polymerization proceeded through cationic rearrangement of the bicyclic ring. ^1^H-NMR showed the presence of a methoxy group at 3.5 ppm from the addition of methanol as chain terminator and incorporation of methoxy group during chain termination. In the absence of methanol, chain termination happened through β-hydride transfer to produce the thermodynamic product, a highly substituted olefin with 125.4 ppm and 129.1 ppm. High catalyst productivity was highly dependent on the flexibility of the ligand (Scheme 49). Flexible ligands 173 and 174 allow β-pinene monomer ease access to the nickel catalytic center, thus increasing polymer chain elongation.

##### 5.3.1.1 Ring-opening metathesis in the polymerization of δ-pinene

**Fig. 15:**
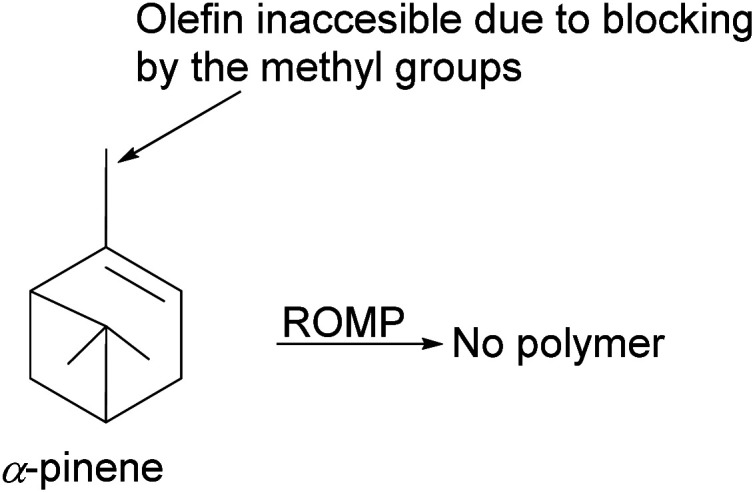
An illustration of the limitation of α-pinene in polymerization.

Despite the ubiquitousness of α-pinene in nature, because of its stability, there are significant challenges in its utility as a chemical feedstock. The olefin in α-pinene is trisubstituted, thus less accessible to steric-sensitive reagents, including metal complex catalysts (Fig. 15). In addition, the cyclobutyl ring's propensity to open to relieve angle strain further complicates its utility in reactions involving cationic or radical species. The use of α-pinene for cationic polymerization suffers from low yields of desired products due to the significant production of side polymer products from isomerization. Therefore, the transformation of its olefinic functionality is needed for its synthetic utility to be fully realized.

**Scheme 50:**
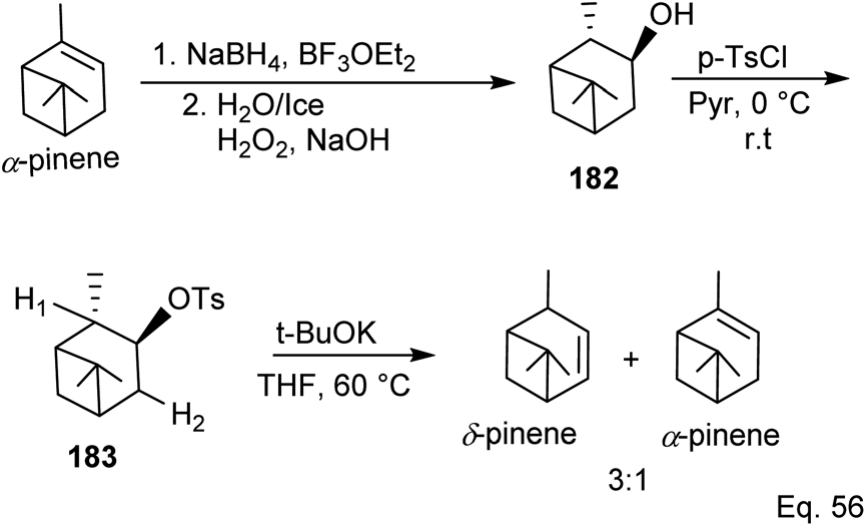
α-Pinene conversion to δ-pinene.

Kennemur and co-workers^86^ demonstrated that α-pinene to δ-pinene isomerization could be carried out in three steps beginning with stereoselective reduction of the olefin with organoboron NaBH_4_ and BF_3_OEt_2_ followed by oxidation with aqueous H_2_O_2_ to produce alcohol 182. Protection of the alcohol with tosylate allowed olefination to δ-pinene by *E*_2_ mechanism. Therefore, the dehydrotosylation can proceed by deprotonating the proton *anti-periplanar* to the tosylate (Scheme 50).

**Scheme 51:**
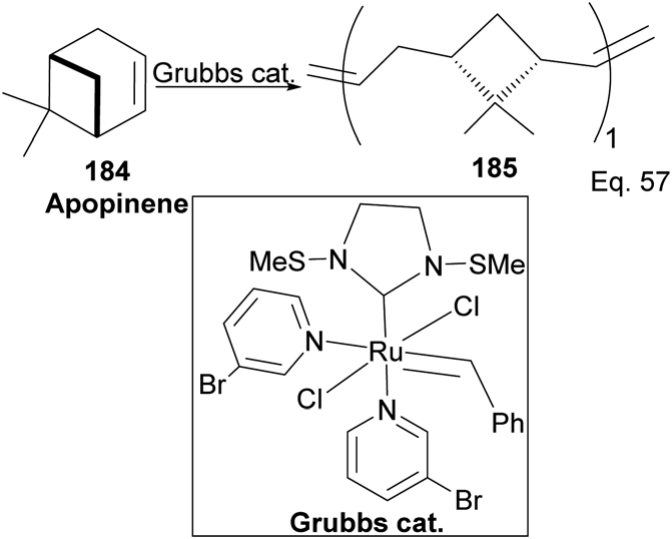
Grubb's-mediated ring opening of apopinene.

Grubb's third-generation catalyst has been studied in ring-opening polymerization of δ-pinene and apopinene 184 (Scheme 51).^87^

**Scheme 52:**
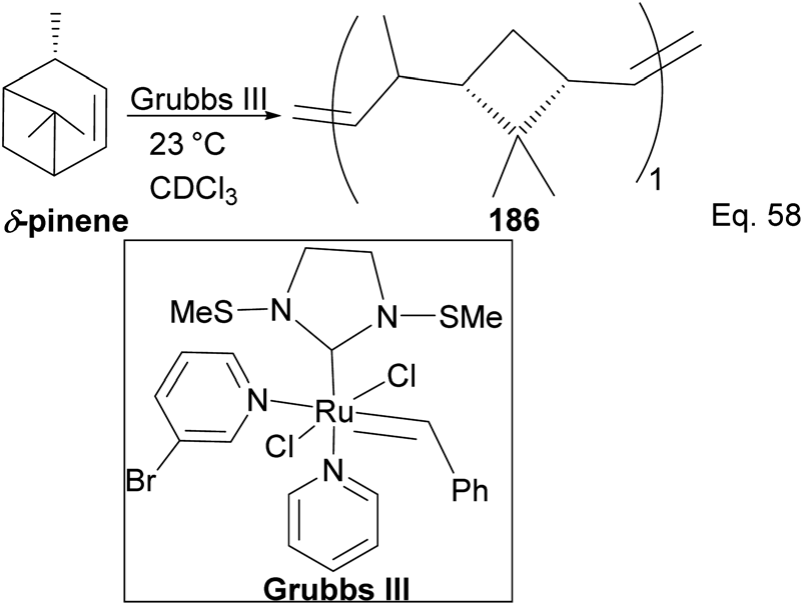
Grubb's mediated ring opening of δ-pinene.

**Scheme 53:**
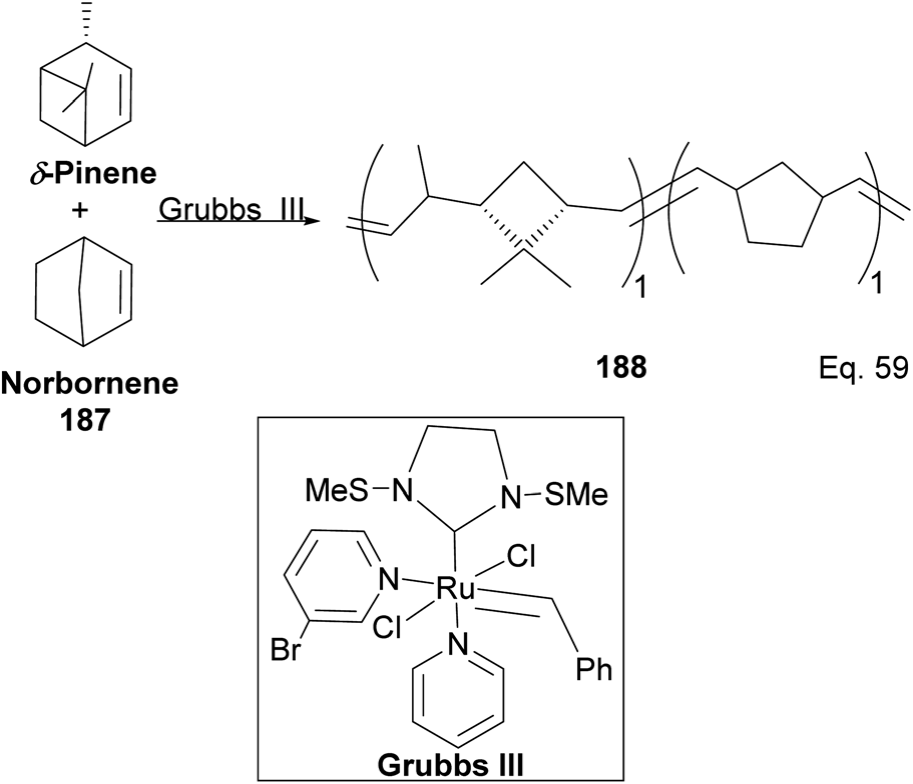
Grubbs III-catalyzed copolymerization of δ-pinene and norbornene.

Kennemur and co-workers reported > 95% yield in their synthesis of poly(δ-pinene) (PδP) 186 (Scheme 52), and the polymer showed a narrow dispersity (<1.2). In addition, the monomer sequences followed regioregularity head to tail arrangement (HT > 95%) micro-structure. The *trans*-olefin accounted for > 98%, and the polymer glass transition temperature *T*_g_ was approximately 104 °C. Specific optical rotation ([α]^24^_589_) in the polymer was +83° ± 1.1° found to be opposite and much higher in magnitude to that of the (−)-δ-pinene ([α]^24^_589_ = −35° (±1.2°)) starting material. PδP has high thermostability. Thermogravimetric studies at ∼337 °C showed only about 5% loss. Furthermore, norbornene and δ-pinene can be polymerized to form block copolymer 188 with a 1 : 1 monomer composition (Scheme 53).

In addition, Gutierrez and Tlenkopatchev solvent-free synthesis of terpene-terminated oligomers from β-pinene and natural rubber (NR) using Grubb's second-generation catalyst (Grubbs catalyst I^88^) showed that the molecular weight of oligomers could be manipulated and controlled through β-pinene NR ratio while maintaining excellent yields between 80–90%.

## 6 Pinene as intermediate for complex natural products

Like other terpenes, pinene is derived from isoprene units, and it possesses a critical number of carbon atoms in its rings, typical of many carbocyclic natural products. The geminal methyl in its bridgehead (signature evidence of isoprene origin) alongside the methyl in its cyclohexyl group provides a convenient scaffold for synthesizing challenging natural products such as Taxol® or longifolene. In addition, the stereogenic centers in pinene are easily transferable to natural products targeted for synthesis since they share the same isoprene origin during their synthesis. Furthermore, the ubiquitous nature of pinene, its availability, the relatively cheap cost of production, and its stability relative to other strained compounds make it an attractive starting material in synthesizing bioactive natural products or natural products-inspired molecules.

### 6.1 Pinene in taxane synthesis

**Fig. 16:**
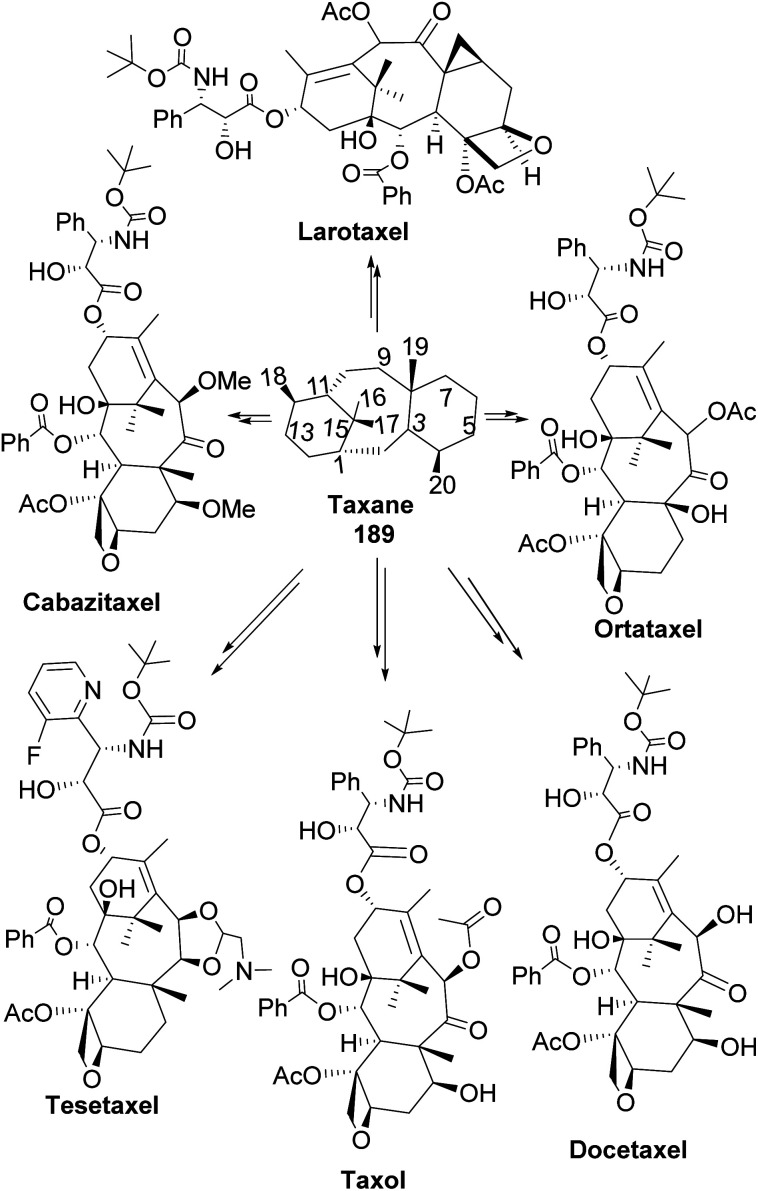
Taxane-based antiproliferative agents.

**Scheme 54:**
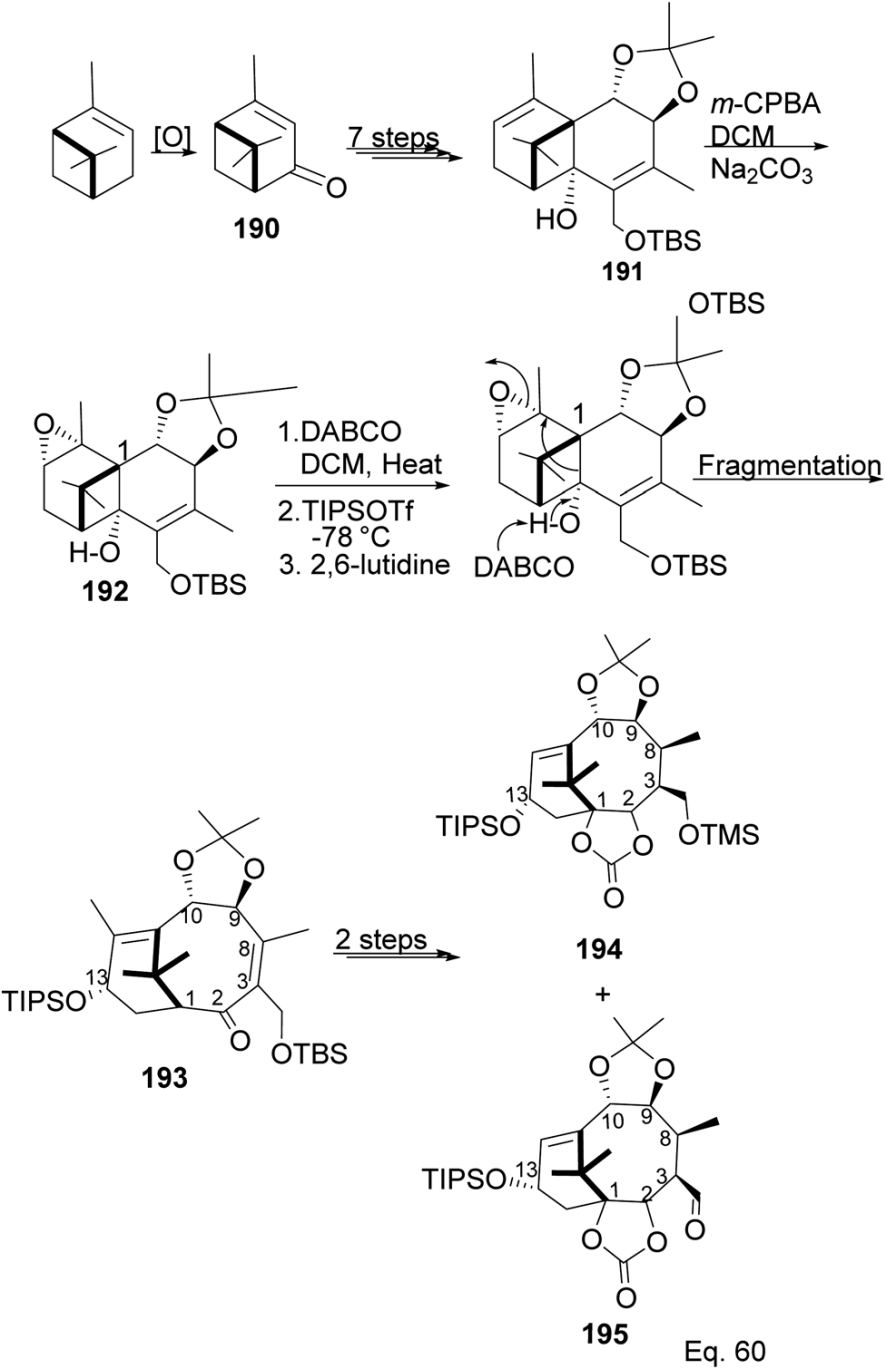
Verbenone made from pinene in the synthesis of taxane derivatives.

Taxane diterpenes, originally isolated from the yew plant (Genus *Taxus*), are essential molecules in medicine. The economic and pharmaceutical importance of the taxane diterpenes led to ongoing research to understand their biosynthesis and pharmacology and to develop efficient organic synthesis routes to produce the challenging scaffold. The anticancer agent Taxol®, the most iconic taxane compound isolated from pacific yew (*Taxus brevifolia*), prevents cell division by binding to tubulin and microtubule.^89,90^ Some of the approved taxane-based antineoplastic drugs (Fig. 16) include paclitaxel, docetaxel, Taxoprexil®, Opaxio®, milataxel, tasetaxel, larotaxel, and ortataxel.^91^ Despite their medicinal importance, the synthesis of taxane and derivatives is still challenging due to their structural complexity and multiple chiral centers. Efforts to prepare them efficiently and cheaply in the laboratory remain an active area of research. Pinene is an attractive starting material for taxane synthesis because it contains the scaffold (ring A) found in taxane. In the synthesis of Taxol® by Wender and co-workers,^92^ verbenone 190, derived from pinene oxidation, was used as starting material. Verbenone 190 was transformed through aldo-condensation and oxidation reactions to obtain a tricyclic intermediate 191. Chemoselective and stereospecific epoxidation of 191 with *m*-CPBA, followed by treatment with bulky base DABCO, to induce hydroxy fragmentation of epoxide 192, produced 8-membered ring intermediate 193 (Scheme 54). The alkoxy formed by the opening of the epoxide ring was protected by TIPS *in situ*. Compound 193 was then transformed to an aldehyde taxane precursor of Taxol®.

**Scheme 55:**
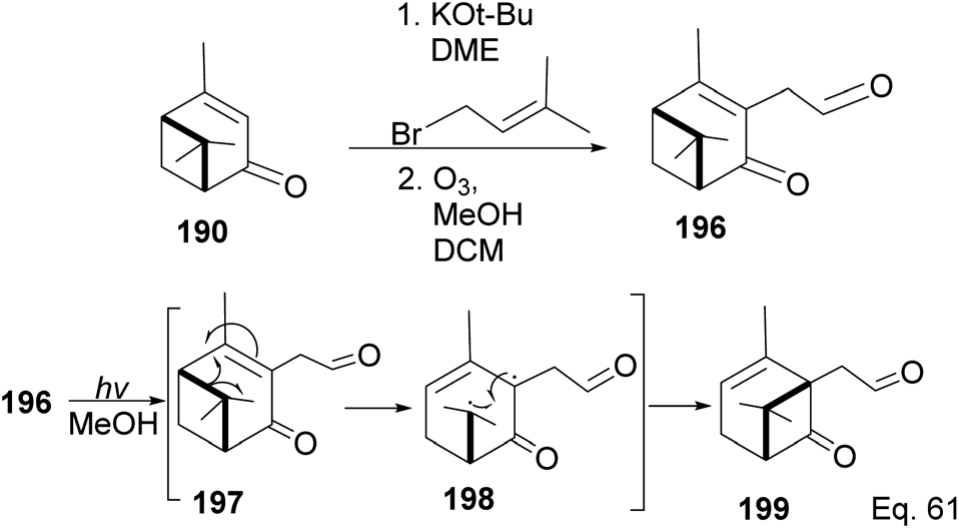
Verbenone (derived from α-pinene) in chrysanthenone synthesis.

In addition to the modification of taxane 194 and 195 in a bid to enhance its pharmacological activities, an advantage of Wender's synthesis of Taxol® from verbenone is that it makes it possible for scaffold precursors such as chrysanthenone 199 (ref. 93 and 94) to be produced through the irradiation of the aldehydic ketone 196 obtained from verbenone transformation (Scheme 55).

### 6.2 α-Pinene in methyl (+)-*trans*-chrysanthemate synthesis

(−)-Phaseic acid is a known metabolite of the plant hormone abscisic acid (ABA).^95^ It plays a key role in stomata opening and closing, thus allowing the plant to control the amount of water loss through transpiration. (−)-Macmillan and co-workers first isolated phaseic acid^96^ from the immature seeds of *Phaseolus multiflorous*, and Milborrow and Sakan established its relative stereochemistry.^97^

**Scheme 56:**
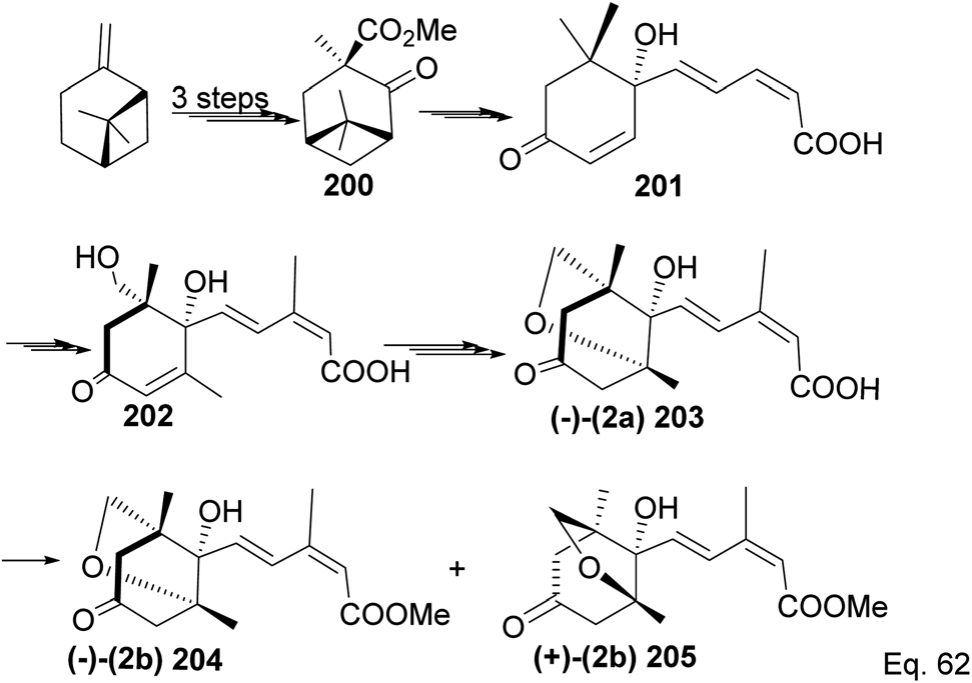
β-Pinene in the synthesis of methyl (+)-*trans*-chrysanthemate.

**Scheme 57:**
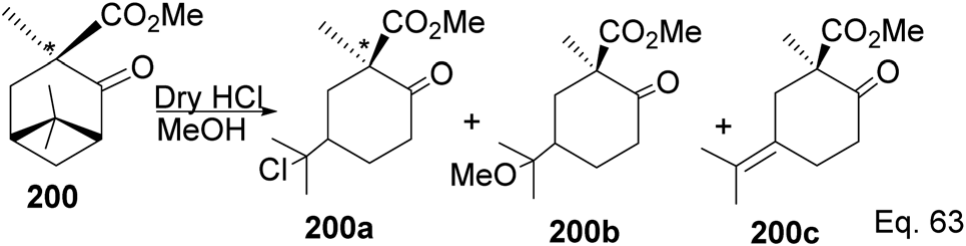
Cationic cyclobutyl ring opening of keto-ester to methyl(*R*)-1-methyl-2-oxo-5-(propan-2-ylidene)cyclohexane-1-carboxylate precursor.

Due to its biological significance and potential use in accessing natural products that bear similar scaffolds, Yamashita and Takahashi^98^ carried out a total synthesis to establish its configuration. Furthermore, their *de novo* synthetic approach provided insight into how to construct chiral bicyclic compounds containing similar skeletons. Because of structural similarities, the key strategic compound for accessing the final molecule is keto-ester 200 (Scheme 56). Compound 200 was prepared in 3 steps from β-pinene. Its treatment with dry methanolic hydrochloric acid (Scheme 57) led to cationic rearrangement to olefinic keto ester 200c along with chlorinated and methoxylated congeners 200a and 200b. Keto esters 200a–c were formed in a 2 : 2 : 1 ratio. The intermolecular addition of the chloro and methoxy groups to the terpenyl 3° carbocation occurred faster than intramolecular olefination to 200c. It is important to note the transfer of the 3*R* chiral center to 6*S* in the quaternary carbon in compound 200 upon the addition of acid.

**Scheme 58:**
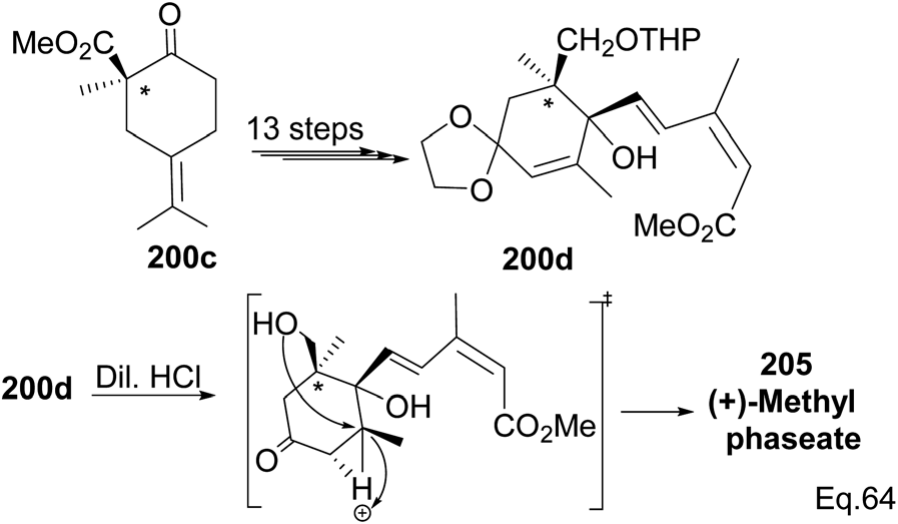
Synthesis of (+)methylphaseate from (*R*)-1-methyl-2-oxo-5-(propan-2-ylidene) cyclohexane-1-carboxylate.

Compound 200c was functionalized *via* linear synthesis to produce conjugated keto ester 200d, and treatment of 200d with dilute HCl led to deprotection of ketone and alcohol from ethan-1,2-diol and tetrahydropyran (THP). At the same time, the hydrogen cation formed through an electrophilic attack on the trisubstituted *endo* olefin triggered a nucleophilic attack from the 1° alcohol to form (+)-methylphaseate (205, Scheme 58).

### 6.3 α-Pinene in the synthesis of garsubellin A

**Fig. 17:**
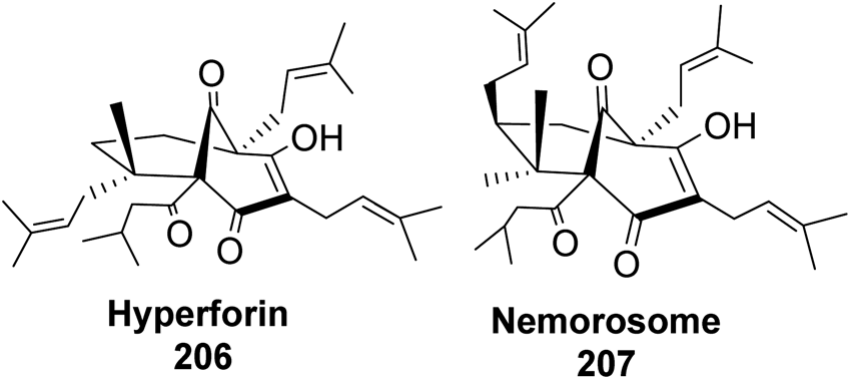
Phloroglucin-like natural products (hyperforin and nemorosome).

Phloroglucin-like natural products such as hyperforin (Fig. 17), berkeleyone A, berkeleydione, huperzine, upia, mexicanolide, rugulosone, nemorosome, and garsubellin A, C, and D^99^ have potential medicinal application in treating inflammation, depression, as well as neurologic disorders such as Alzheimer's disease.

**Scheme 59:**
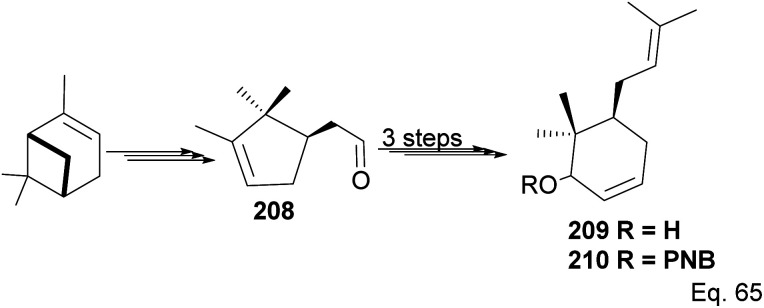
Synthesis of prenylated 6,6-dimethylcyclohex-2-en-1-ol from (+)-campholenic aldehyde derived from (−)-α-pinene.

The core phloroglucin scaffold is a heavily indented bicyclic ketone containing bicyclo [3.3.1] nonane skeleton. Several approaches in its synthesis have been reported. One synthetic approach was carried out by Mehta and Bera using (−)-α-pinene as starting material and the source of (+)-campholenic aldehyde 208, crucial for further stereospecific transformation and enantioselective functionalization to produce key intermediate 214. (+)-Campholenic aldehyde 208 was prepared from the epoxidation of pinene and a Lewis acid-mediated isomerization. Aldehyde 208 was transformed to allylic alcohol 209 in 3 steps synthesis (Scheme 59).

**Scheme 60:**
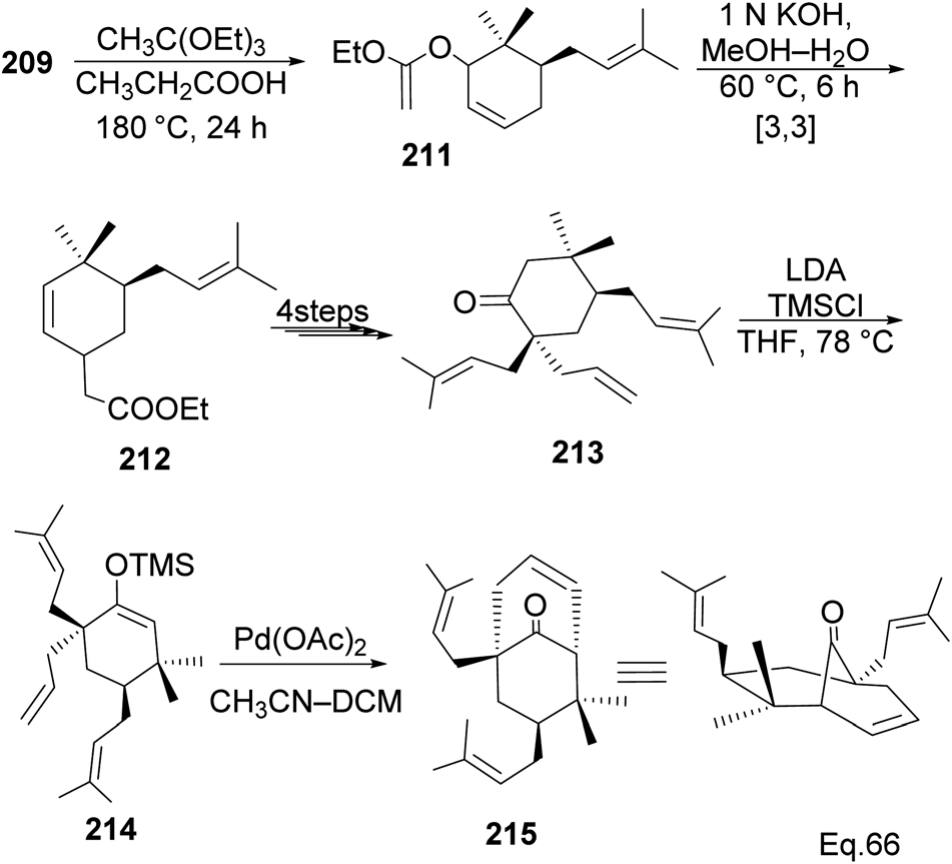
Transformation of 6,6-dimethylcyclohex-2-en-1-ol to phloroglucin skeleton.

**Fig. 18:**
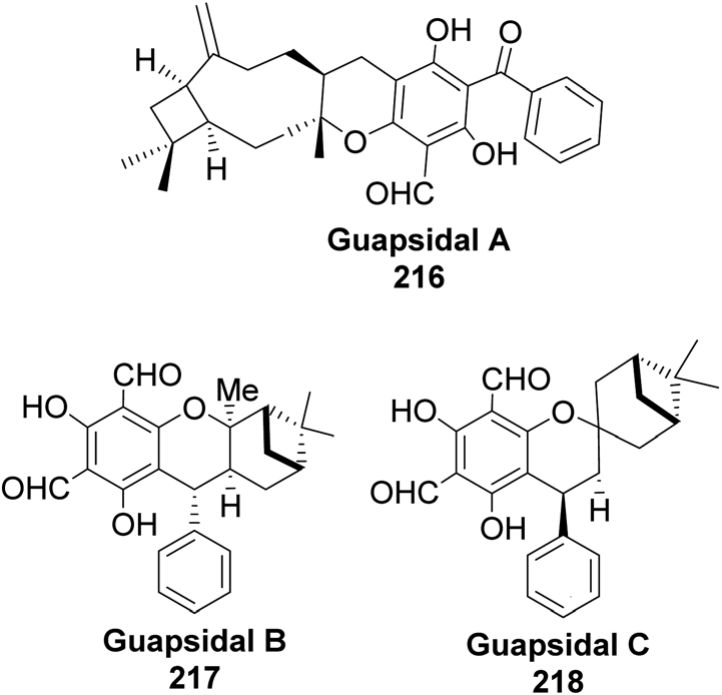
Structures of guapsidal A–C.

**Scheme 61:**
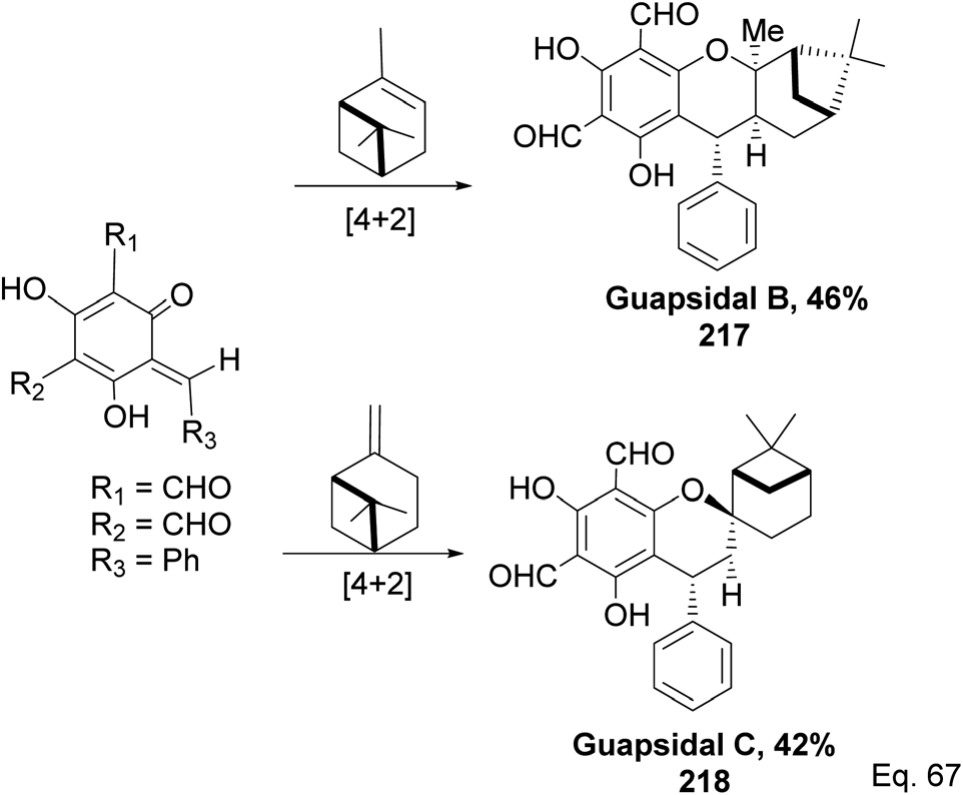
Biomimetic synthesis of guapsidals B and C from pinene and *o*-quinone methide.

**Scheme 62:**
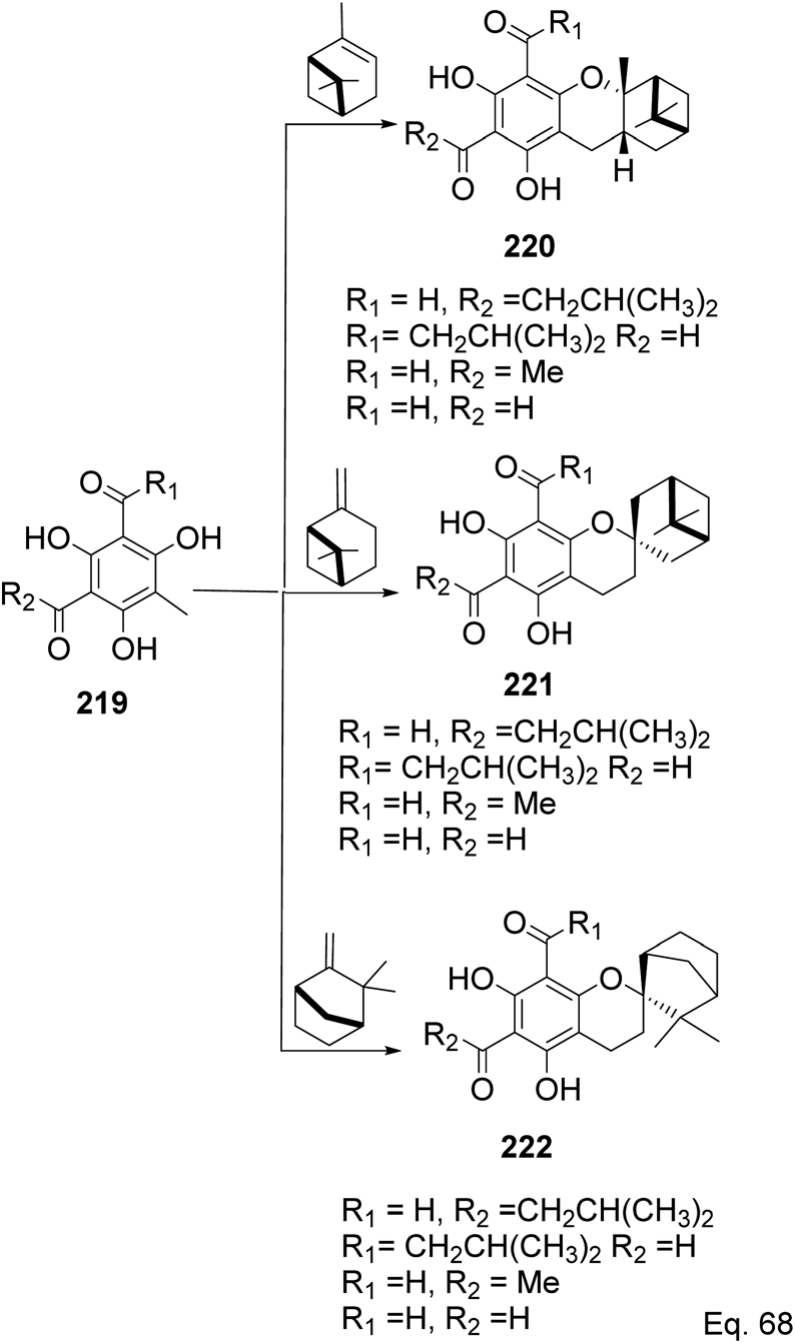
Biomimetic synthesis of *S*-euglobals from pinene and *o*-quinone methide.

**Scheme 63:**
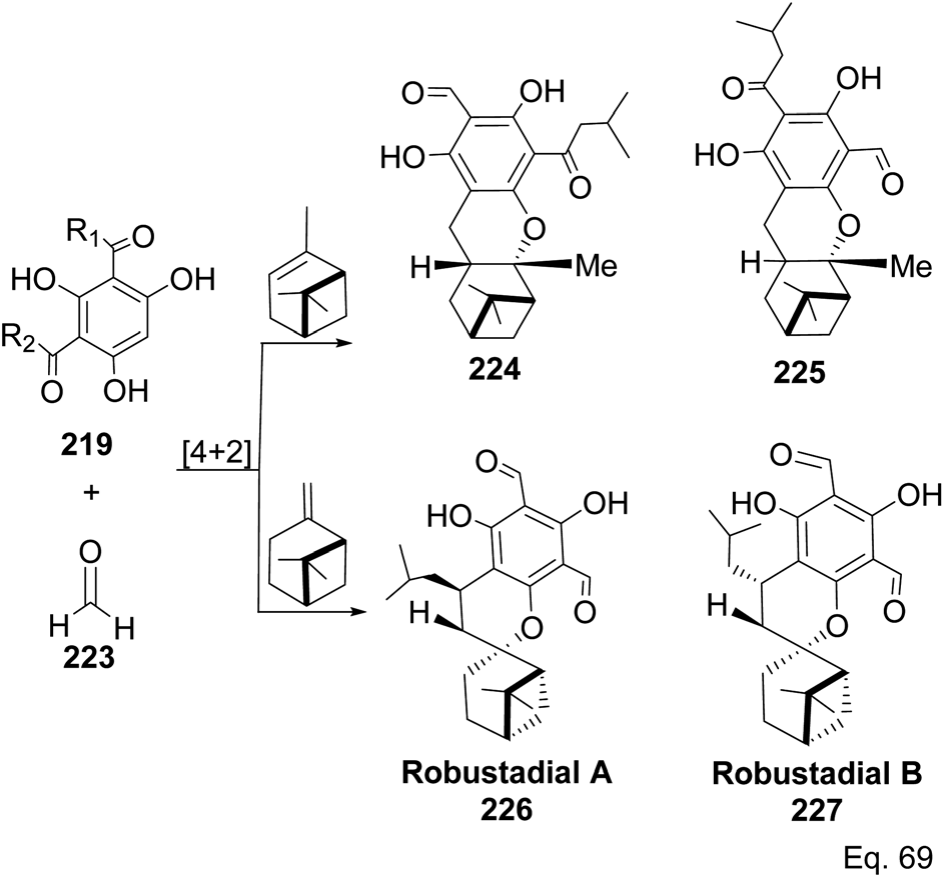
Biomimetic synthesis of *S*-euglobals derivatives from pinene isomers and *o*-quinone methide formed *in situ* from formaldehyde and trihydroxylated benzaldehyde isomers 219.

**Scheme 64:**
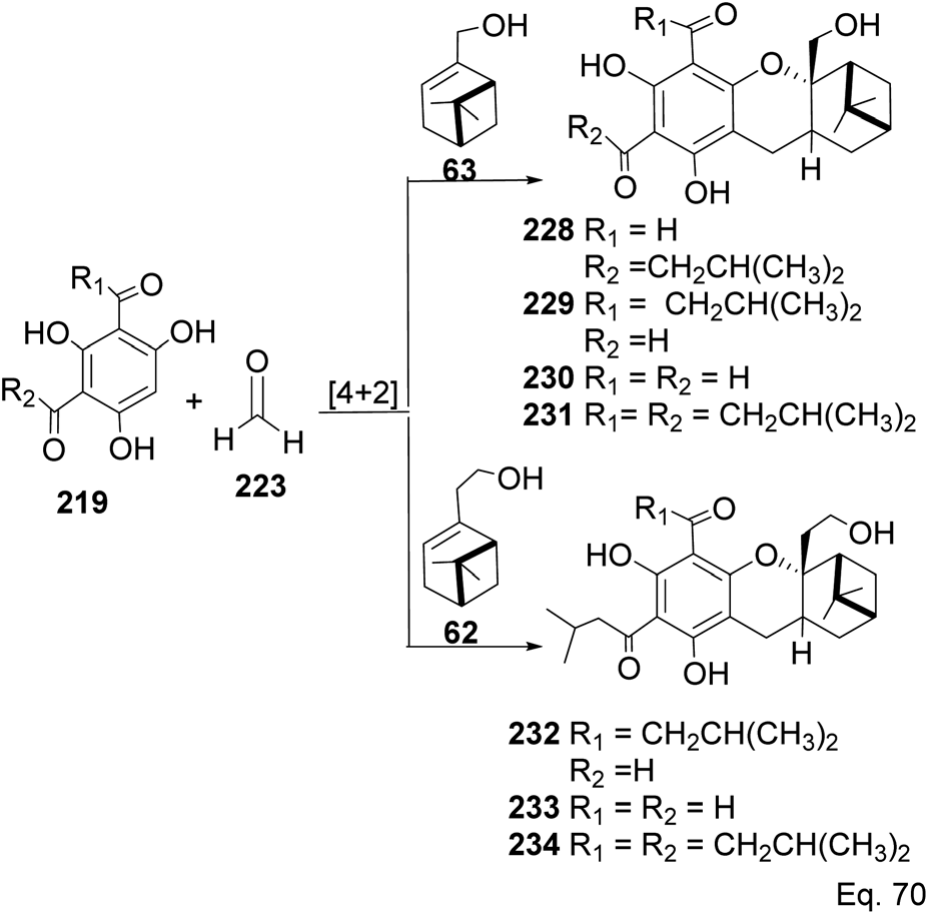
Biomimetic synthesis of *S*-euglobals derivatives from nopol/myrtenol and *o*-quinone methide.

Allylic alcohol 209 was converted to *ortho* ester 211 and used for base-induced Claisen rearrangement to introduce prenyl unit in ester 212. Several transformations of 212 (Scheme 60) led to a highly prenylated silyl enol ether (key intermediate) 214. Treatment of 214 with Pd(OAc)_2_-induced intramolecular cyclization through (less sterically hindered terminal) allylic attack on silyl enol ether produced phloroglucin skeleton 215 in 30% yield from 213.^100^ Sesquiterpenes guapsidal A-C (216–218, Fig. 18) of the meroterpenoid class are commonly found in plants belonging to the genus *Psidium* (Myrtaceae), including *Psidium gujava* L. The guapsidals have been investigated for the treatment of diarrhea and diabetes.^101^ The core guapsidal structure contains a chromane skeleton fused with terpenes such as pinene or methylenecyclononane. The fusion with terpenes happens through a tetrahydro-2*H*-pyran ring resulting in a spirocyclic or decalin [4*a*,8*a*] motif with many chiral centers. Therefore, synthesizing the guapsidals can be rather challenging Since Guapsidal B and C have an inbuilt pinene moiety, its synthesis has been shortened and made inexpensive by making it from cheaply available pinene isomers and *o*-quinone methide. Guapsidal B in 46% yield and C in 42% have been biomimetically prepared by Maiti and co-workers using (+)-α-pinene and (−)-β-pinene, respectively with *o*-quinone methide in hetero-Diels–Alder reaction (Scheme 61).^102^ The biomimetic synthesis of antiparasitic *S*-euglobals like 220 (ref. 103) started from α-pinene using [4 + 2] hetero-Diels–Alder reaction (Scheme 62), while the spirocyclic analogs of euglobals G (221 and 222) were made from β-pinene and camphene scaffolds. Singh and co-workers also prepared a variety of euglobals analogs 217, 224–230, 234 from phloroglucinol using Knoevenagel condensation of polyphenol 219 and formaldehyde 223 followed by a [4 + 2]-Diels–Alder cycloaddition reaction, using *o*-quinone methide, with α-, and β-pinene (Scheme 63), nopol 62 and myrtenol 63 (Scheme 64). The *S*-Euglobals were evaluated for antimicrobial activities against methicillin-resistant *Staphylococcus aureus* (MRSA), *Leishmania donovani*, and fungal species *Candida glabrata* and *C. krusei*. The *Leishmania* assay showed that 224, 225, and 232 have moderate antileishmanial activities (IC_50_ = 7.1, 3.6, and 9.5 μg mL^−1^, respectively).

### 6.4 (−)-β-Pinene-derived N-heterocyclic carbenes

**Fig. 19:**
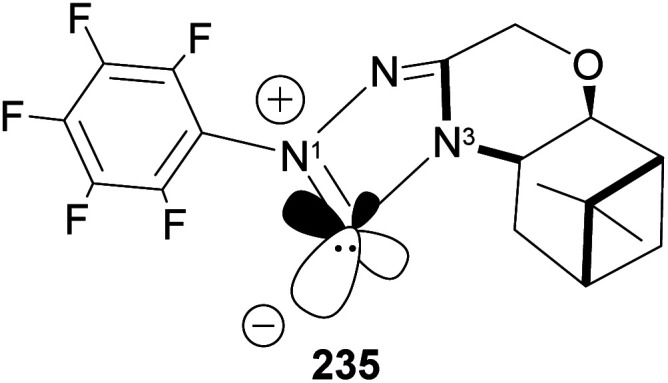
β-Pinene-based triazolium carbene catalyst.

*N*-Heterocyclic carbenes (NHCs) are important ancillary ligand and catalysts in asymmetric synthesis. Like phosphine ligands, NHCs are good σ-donor and are electronically and sterically tunable. Unlike phosphine ligands, NHCs are relatively inert and bind strongly (through sp^2^ carbon) to the metal center, hence reaction can occur with low ligand concentration. NHCs ligands are stable because the lone pairs on the imidazole nitrogen atoms (N1 and N3) are delocalized to the empty p-orbital of the carbene carbon. Lone pair delocalization in triplet state is more stable than singlet state by >80 kcal mol^−1^. The filled sp-orbital donates electron pair to the metal center, as shown in 235 (Fig. 19), and the empty p-orbital is available for π-back donation from the metal center. Substituents at nitrogen can significantly influence the *stereo*-electronic behaviors of NHC ligands. A significant drawback of NHCs is that reductive elimination can happen *via* methylation of the imidazole to form an imidazolium ion.

**Scheme 65:**
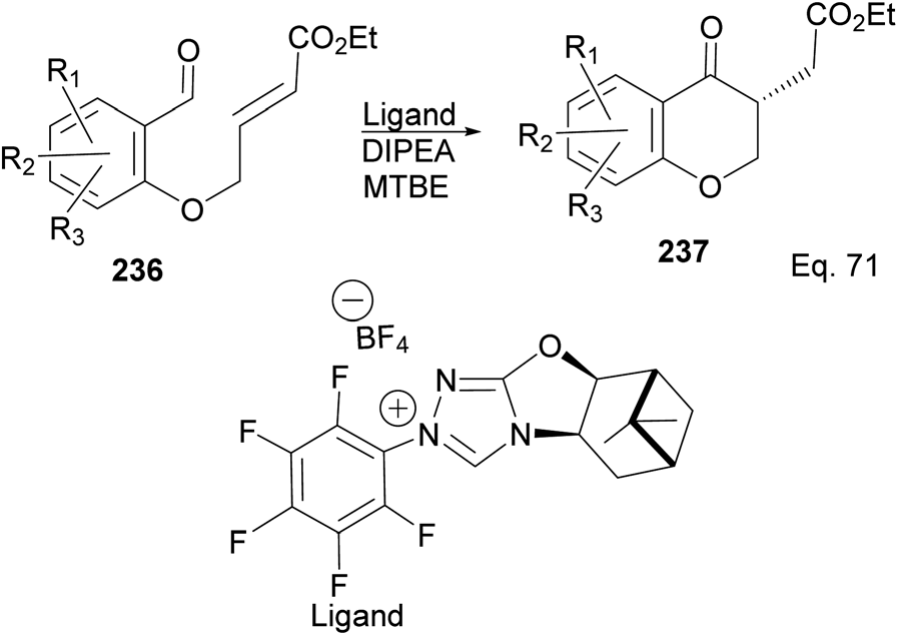
NHC 235 in the synthesis of the derivatives of 4-chromanone esters *via* Stetter reaction.

The incorporation of β-pinene in triazolium carbene catalyst has been shown to add chirality to the catalytic complex. An inbuilt chirality within the complex is important for asymmetric induction to form the desired product. Rafinski and co-workers^104^ have reported the successful enantioselective synthesis of 4-chromanone derivatives using β-pinene-based triazolium through intramolecular Stetter reaction (Scheme 65).

**Scheme 66:**
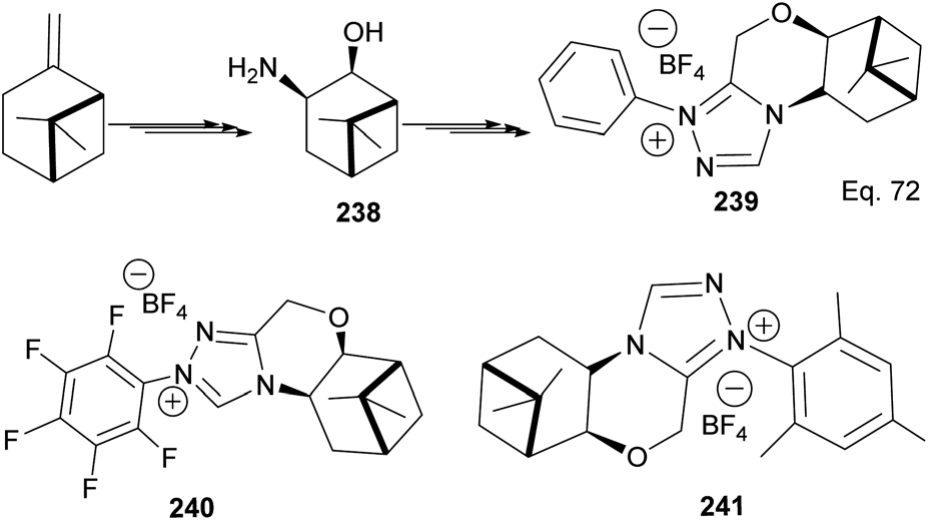
Synthesis of β-pinene-based triazolium carbene derivatives.

**Scheme 67:**
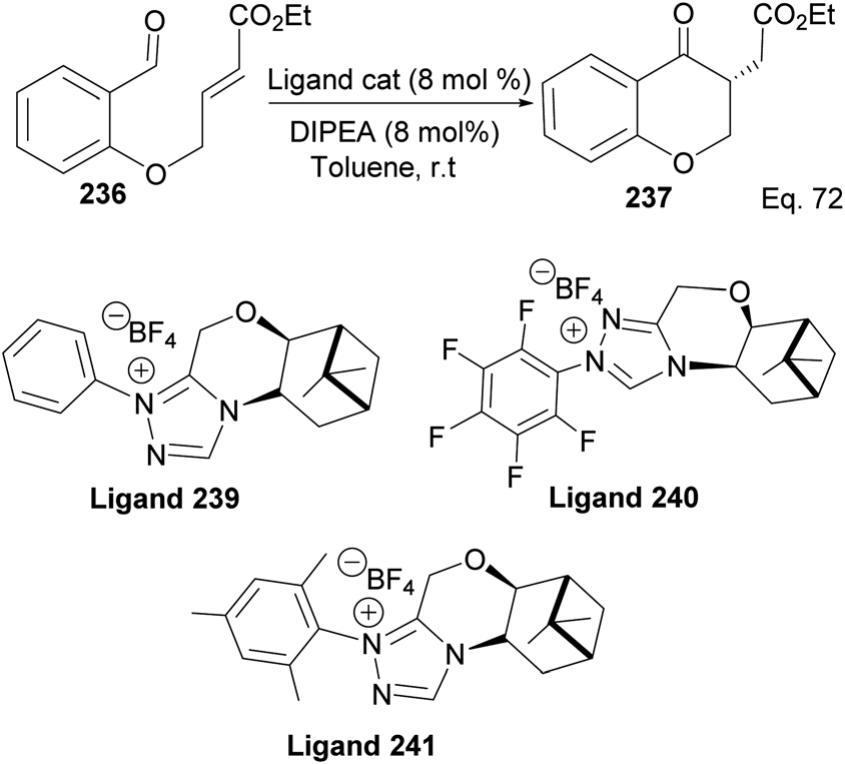
Pinene-based NHC ligands in the synthesis of 4-chromanone ester.

The 3 triazolium species 239–241 derived from β-pinene moiety shown in Scheme 66 were prepared from amino alcohol 238 in 3 steps. The intramolecular cyclization to 4-chromanone proceeded with low (10%) to high (99%) yield but with excellent enantioselectivity > 95 : 5%. For example, the intramolecular cyclization of 236 using ligand 241 produced 237 in 31% yield and 99 : 1 ee*.* In the presence of ligand 240, the yield produced was 99% with 97 : 3 ee, but ligand 239 was inactive (Scheme 67).

**Scheme 68:**
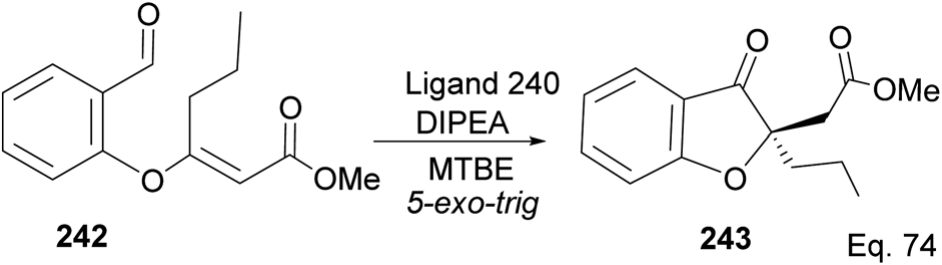
NHC-pinene ligand promoted intramolecular cyclization of methyl-(*E*)-3-(2-formylphenoxy) hex-2-enoate.

Cyclization requires activation of the olefin acceptor group by electron-withdrawing group (EWG). For instance, cyclization of compound 242 to 243 proceeded with 92% yield and 95% ee, in the presence of ligand 240, because of the presence of methyl acrylate moiety, which acts as Michael acceptor. The nucleophilic attack from aldehyde (Scheme 68) followed a similar path seen in the 1,4-addition reaction.

**Scheme 69:**
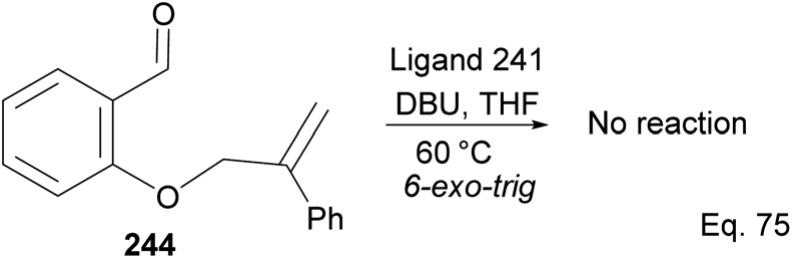
Attempted cyclization of 2-((2-phenylallyl)oxy)benzaldehyde using NHC-pinene ligand as catalyst.

Attempt to cyclize 244 in the presence of ligand 241 (Scheme 69) failed because its inactive double bond lacks an olefinic activator that can enable 1,4-cyclization. In this case, 1,2-addition is the only path for cyclization.

**Scheme 70:**
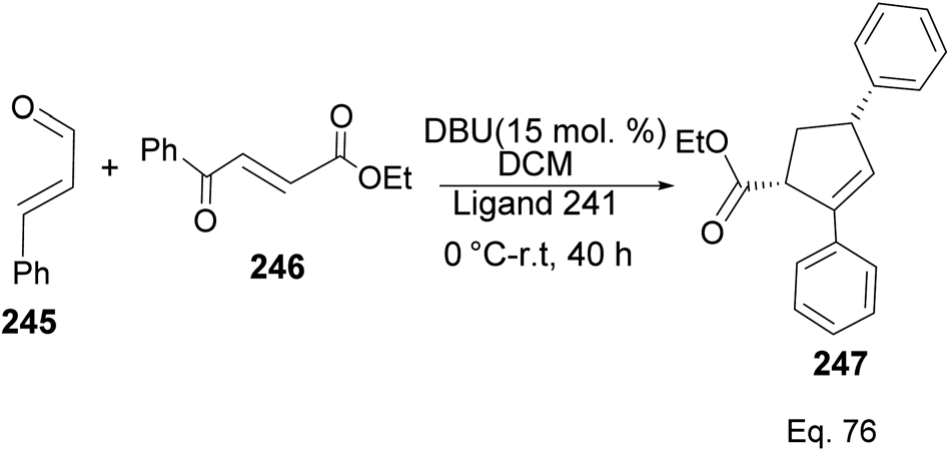
NHC-pinene assisted intermolecular synthesis of ethyl-(4*S*)-2,4-diphenylcyclopent-2-ene-1-carboxylate.

Ligand 241 was also used to synthesize cyclopentene product 247 (Scheme 70) obtained in 71% yield with 96% ee, and 8 : 2 dr, from 1,4-α,β-aldehyde and 1,4-α,β-ketonic ester through a benzoin-oxy-Cope rearrangement reaction.

**Scheme 71:**
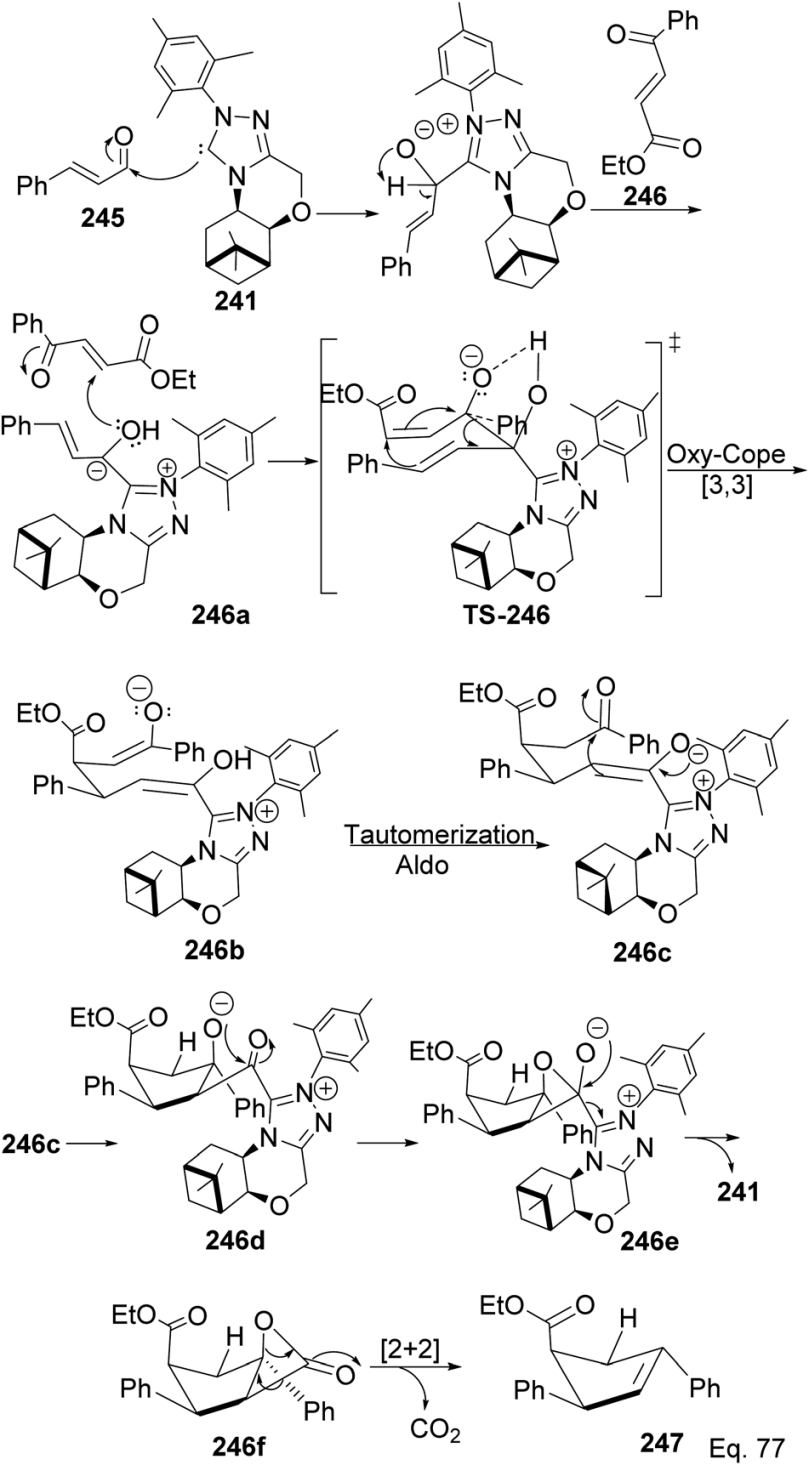
Mechanistic steps in the synthesis of ethyl-(4*S*)-2,4-diphenylcyclopent-2-ene-1-carboxylate.

The formation of 247 (Scheme 71) is complex and intriguing. It starts with the activation of aldehyde 245 with NHC 241 to generate carbanion 246a through the loss of proton sharing the same carbon with the alkoxide. Carbanion 246a attacks ketone 246 to afford an alkoxide adduct which undergoes stereospecific [3,3]-oxy-Cope rearrangement to form 246b. The subsequent intramolecular tautomerization–aldo reaction of 246b led to ring closure in 246c to produce cyclopentane alkoxide 246d, which cyclizes to β-lactone 246f and regenerate NHC-catalyst 241. Highly strained β-lactone^105^246f undergoes rapidly [2 + 2] cycloreversion to afford the target product 247.

### 6.5 (−)-β-Pinene in the synthesis of (−)-β-pinene-based amides and acylthioureas

**Scheme 72:**
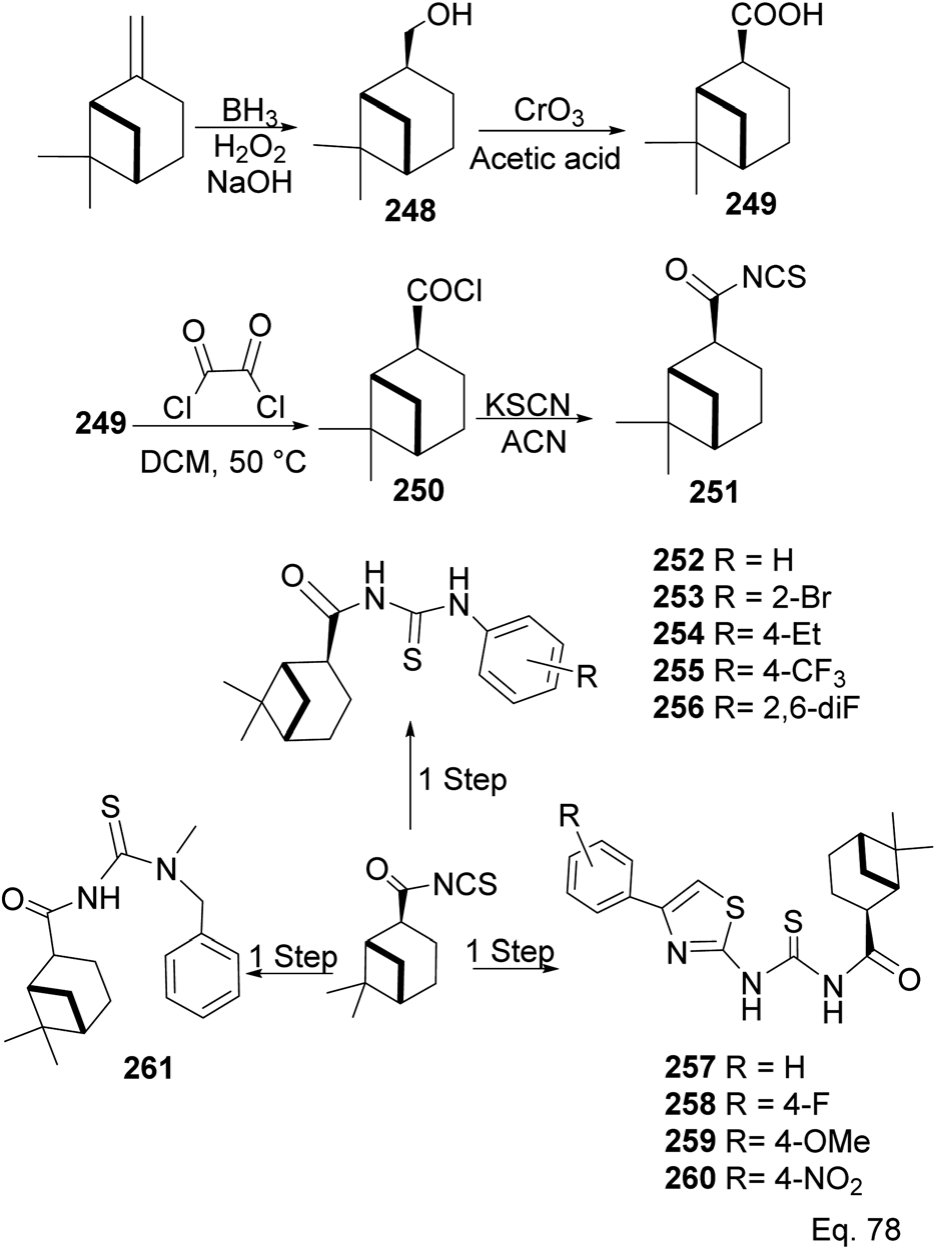
Synthesis of β-pinene-based acylthiourea.

**Scheme 73:**
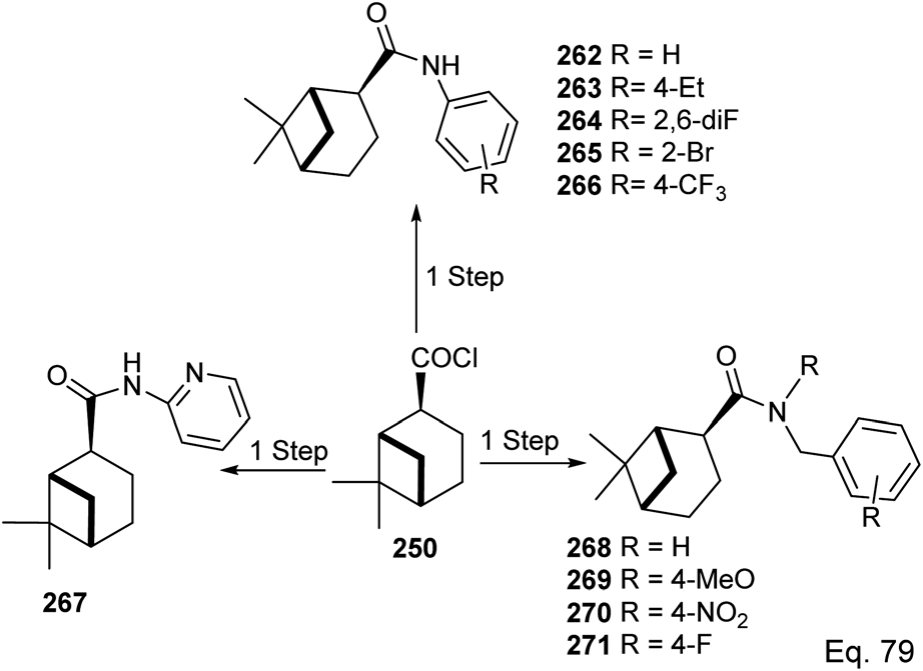
Synthesis of β-pinene-based amides.

In the investigation of potential anticancer (breast and colon cancer) compounds with (−)-β-pinene moiety, Song and his co-workers^106^ used myrtanoic 249 from two-step oxidation of (−)-β-pinene. Myrtanoic acid was reduced to its corresponding thiocyanate 251 with acylhalide 250. Amination of thiocyanate 251 produced 252–261 (Scheme 72). The Amides 262–271 (Scheme 73) were produced from the amination of acylhalide 250.

**Scheme 74:**
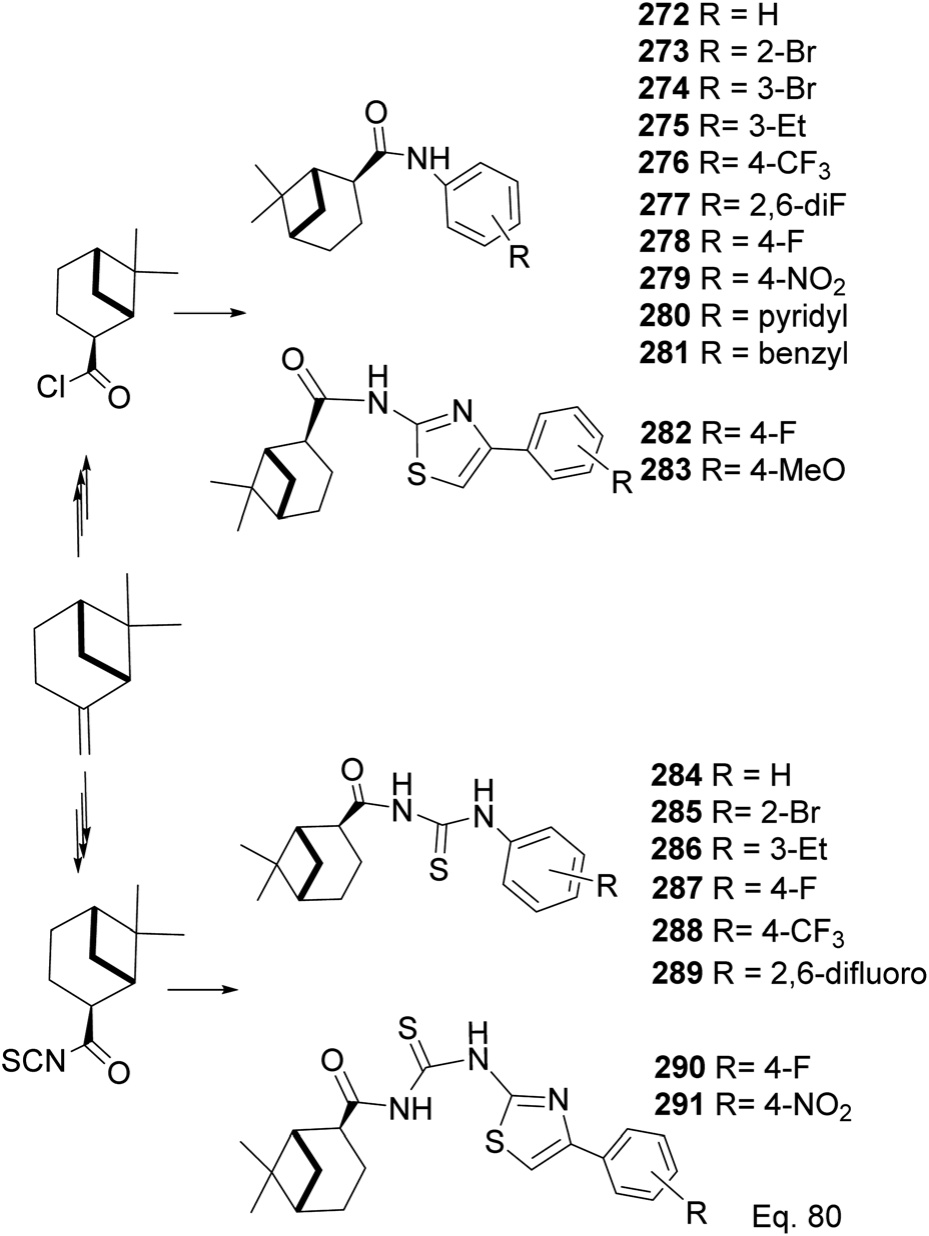
Synthesis of β-pinene-based acylthiourea 272–291.

In a similar study on pinene-derived amides (272–283) and thioureas (284–291, Scheme 74), Song^107^ and co-workers prepared a series of thiocyanates and amides like those described in Schemes 72 and 73. They tested the molecules against plant fungi species (*Colletotrichum gloeosporioides*, *Fusarium proliferatum*, *Alternaria kikucshiana*, *Phomopsis* sp., and *Phytophthora capsica*) as potential crop protection agents.

### 6.6 (−)-β-Pinene in the synthesis of (+)-nootkatone

**Scheme 75:**
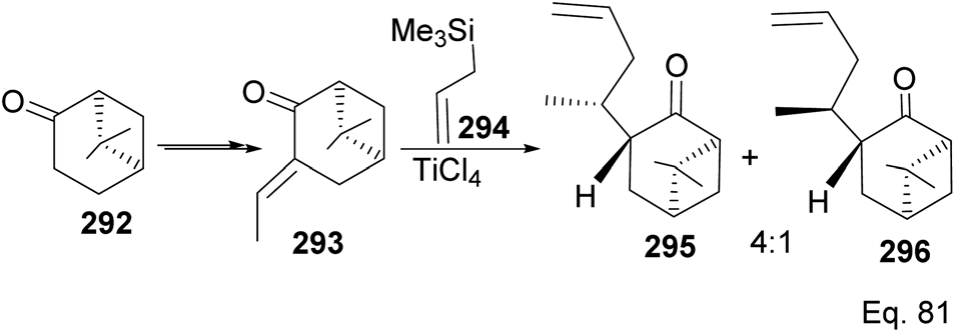
TiCl_4_-mediated synthesis of allylic nopinone enantiomer 295 and 296 (precursors in the preparation of (+)-nootkatone and its derivatives).

The grapefruit sesquiterpenoid Nootkatone inhibits acetylcholinesterase in insects and is a potent insecticidal.^108^ Due to its potent insecticidal activity, it has been studied for mosquitoes and agricultural pest control.^109^ Short synthetic routes and high yield are important for the commercial viability of most natural products. There have been several approaches to make nootkatone skeleton and various derivatives. For example, Revial and Pfau^110^ used an enantioselective Michael addition reaction to prepare (+)-valencenol from protected 2-methylcyclohexane-1,4-dione and phenyl crotonate. (−)-β-Pinene can also be used as starting material in synthesizing molecules with decalin skeletons such as nootkatone and valencene. Oxidation of (−)-β-pinene to nopinone 292 provides a good starting material in nootkatone synthesis, as demonstrated by Torri and co-workers.^111^ Moreover, Yoshikoshi and colleagues prepared nootkatone from nopinone. The key dione intermediate for the nootkatone core structure was obtained from the transformation of 292 in Yoshikoshi's synthesis (Scheme 75).^112^

**Scheme 76:**
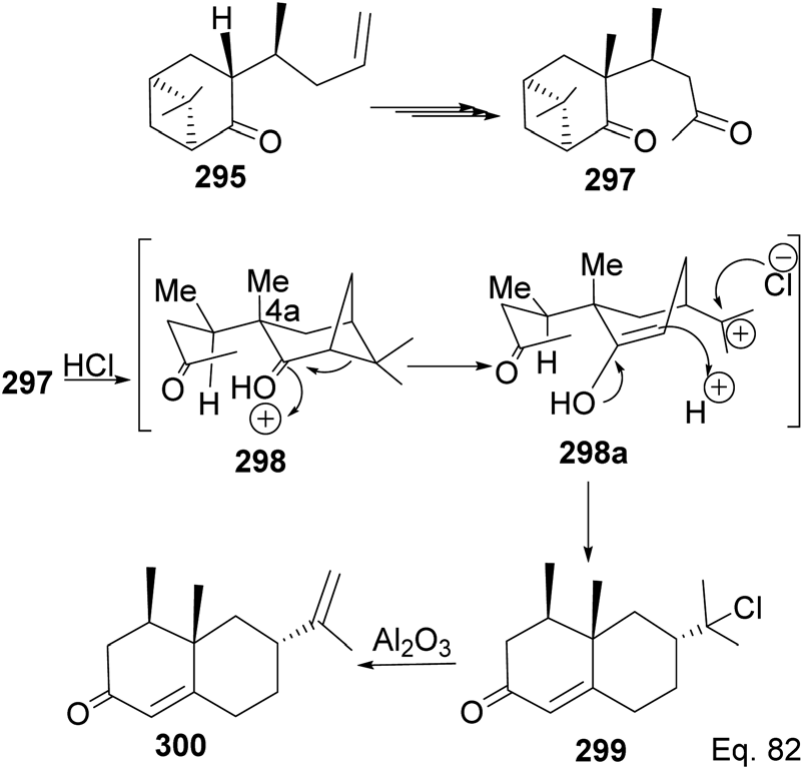
Stereoselective transformation of 295 to (+)-nootkatone.

**Scheme 77:**
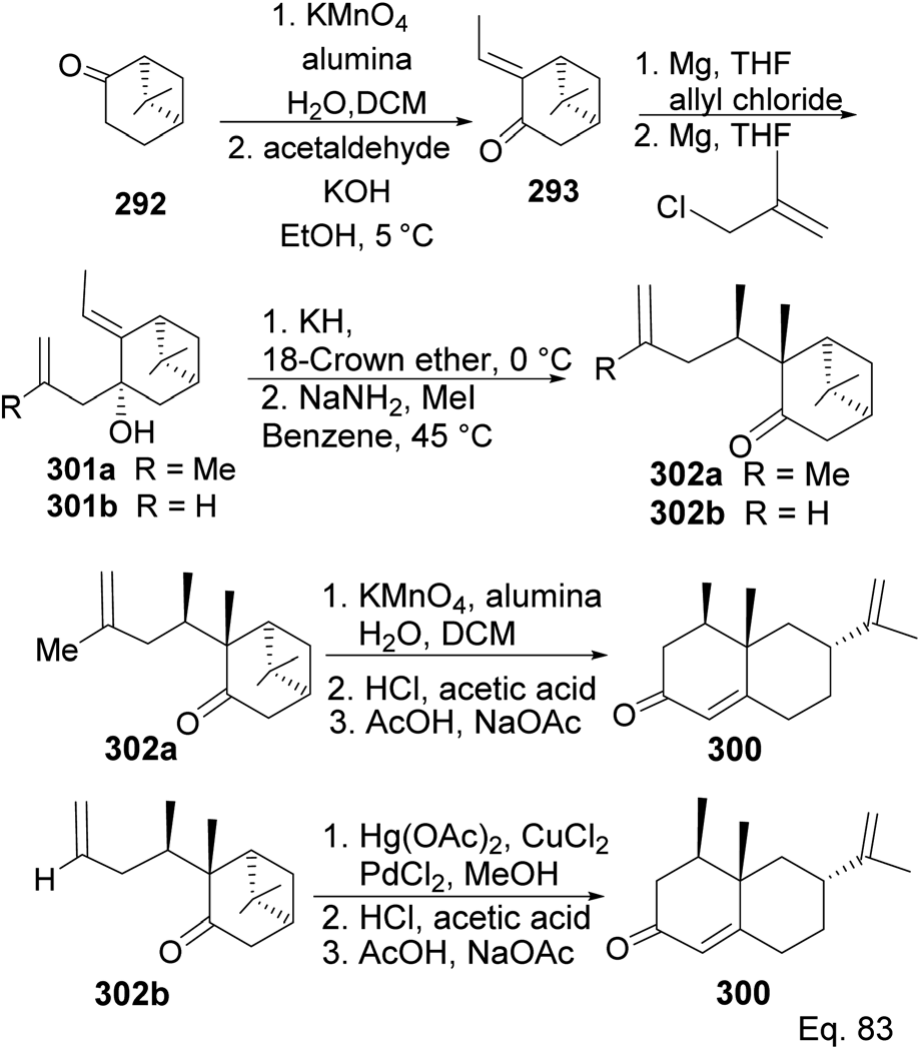
Synthesis of (+)-nootkatone from nopinone *via* stereospecific oxy-Cope rearrangement of (*E*)-3-allyl-2-ethylidene-6,6-dimethylbicyclo[3.1.1]heptan-3-ol (301).

**Scheme 78:**
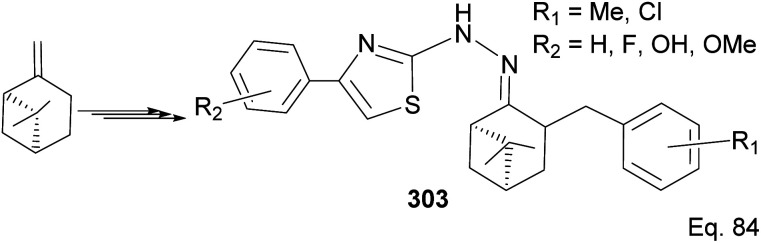
Synthesis of (−)-β-pinene-based thiazole.

Allylation of 292 in TiCl_4_ catalyzed reaction led to inseparable *exo*-olefinic stereoisomers 295 and 296 in a 4 : 1 ratio. Methylation of tertiary C-4a and functionalization of the olefin to ketone afforded dione 297. Cyclization of 297*via* HCl acid-catalyzed cyclobutyl ring open in 298 followed by aldol condensation of 291a produced chloro-nootkatone 299, and the dehydrochlorination of 299 led to nootkatone 300 (Scheme 76). The lack of sufficient stereochemical control is the major drawback of Yoshikoshi's synthesis of nootkatone. Introducing a methyl group at the C-4a quaternary center poses a serious challenge because the vicinal methyl impedes the top face methylation. Also, the dimethyl in the bridgehead on the opposite side of the ring blocks bottom face methylation. Furthermore, the chiral center at C-4a poses a steric challenge (*syn*-pentane) during butane cleavage of the aldol condensation. To overcome the stereochemical challenges at C-4a while employing (−)-β-pinene and acid-mediated aldol cyclization to access nootkatone, Crowe and Sauer (Scheme 78) allylated the ketone functional group (1,2-addition) instead of the olefinic functional group (1,4-addition) in α,β-unsaturated ketone 293 (Scheme 77). The alcohol 301a–b obtained was subjected to base-catalyzed stereospecific oxy-Cope rearrangement^113^ to produce ketone 302a–b. Oxidation of olefinic site to ketone and the addition of acid led to cyclized nootkatone 300.^114,115^

### 6.7 (−)-β-Pinene in the synthesis of (−)-β-pinene-based thiazole

Wang and co-workers have reported the synthesis of thiazole derivatives 303 from (−)-β-pinene^116^ as potential antineoplastic agents (Scheme 78). The active molecules increased reactive oxygen activities (ROS) in the mitochondrial membrane and caused apoptosis in HeLa cells.

### 6.8 (−)-β-Pinene in the synthesis of 2-amino-3-cyanopyridines

**Scheme 79:**
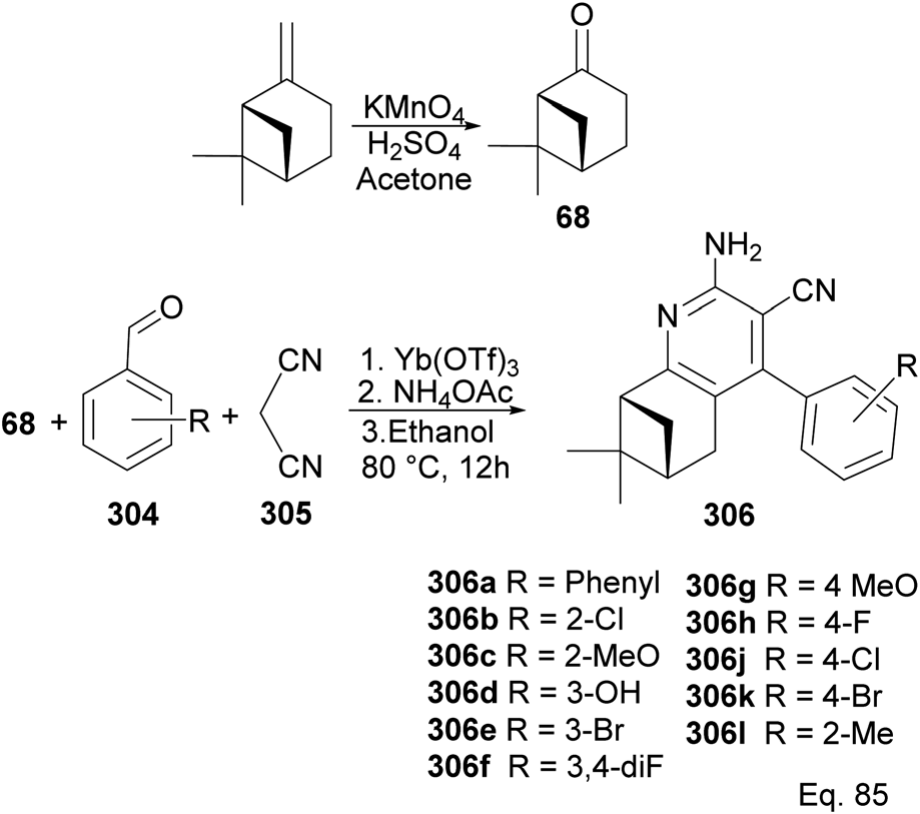
Synthesis of 2-amino-3-cyanopyridines from (+)-nopinone.

In Song's synthesis of 2-amino-3-cyanopyridines 306a–l as potential antineoplastic agents *via* a (+)-nopinone moiety, a single pot aldol-condensation (Scheme 79) reaction involving Yb(OTf)_3_, NH_4_OAc, nopinone, aldehyde 304, and 1,1-dicyanomethelene 305 was used.^117^ The yield from reactions varied depending on substituents on the aldehyde substrate.

## 7 α-Pinene in atmospheric chemistry

Monoterpenes like α/β-pinene constitute a significant class of volatile organic compounds (VOCs) emitted to the atmosphere by plants. Went and Rasmussen produced seminal reports to link the haze observed over vegetated landmasses with the release of volatile organic materials from plants and speculated about the role of photochemical transformation of the organic materials to particulates in the blue aerosol (haze).^118–120^ α-Pinene is now known as the most abundant monoterpene in the troposphere. The monoterpene's major contributor to the atmosphere is the coniferous forest ecosystem of the northern hemisphere.^121,122^ Atmospheric photooxidation reactions by terpenes like α/β-pinene with ozone, hydroxide radical, and nitrogen oxides (NO and NO_2_) are a significant part of the secondary organic aerosols.^123^

### 7.1 Ozonolysis of α-pinene

**Scheme 80:**
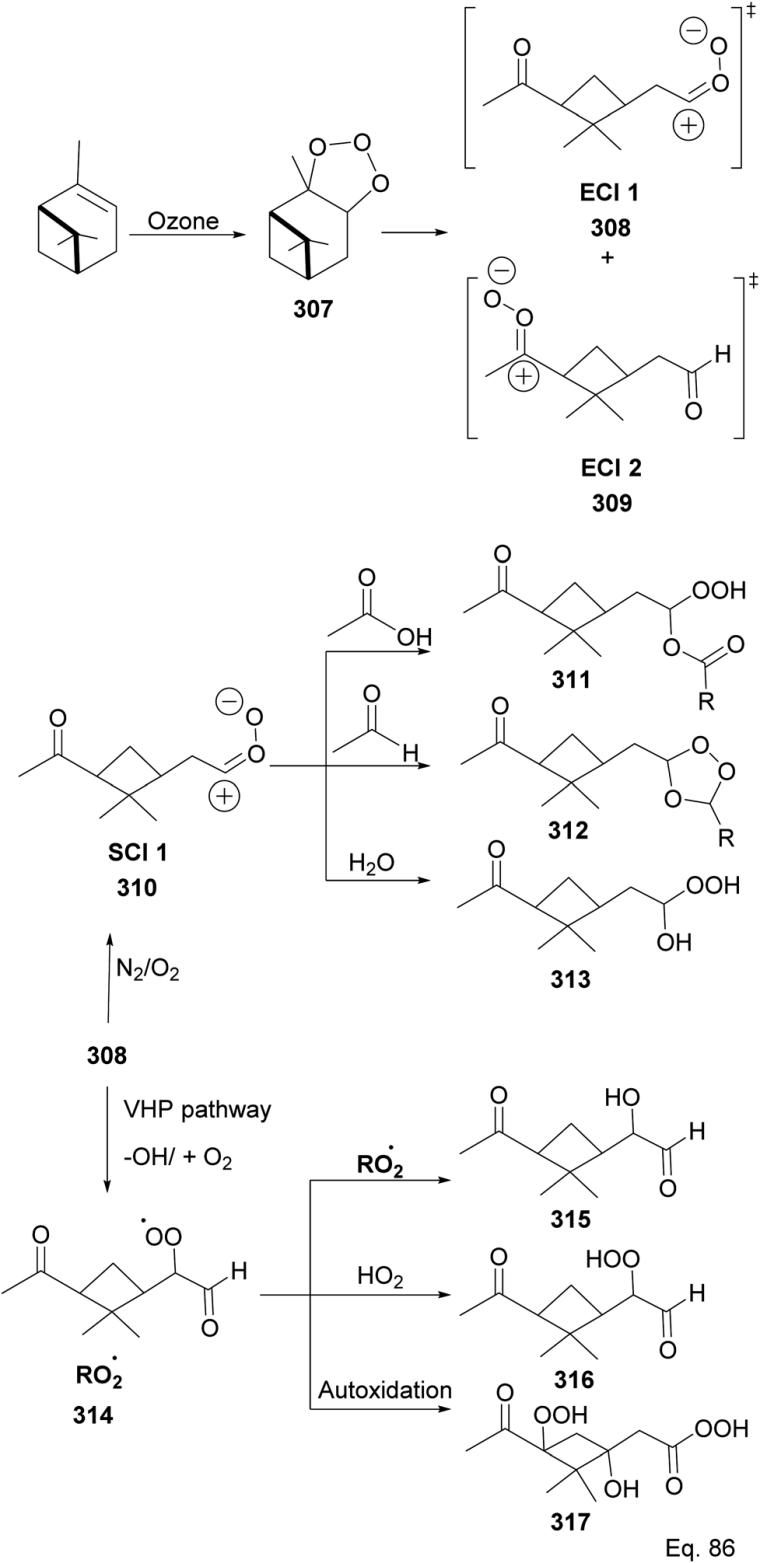
Photooxidation products from ozonolysis of α-pinene.

Because α-pinene constitutes close to 50% of global monoterpene emissions, numerous investigators have studied the ozonolysis of α-pinene to identify and characterize gaseous products and the components of the particle phase secondary organic aerosol (SOA), including highly-oxygenated multifunctional compounds (HOMS), formed through the reaction.^124–135^ Several components of the SOA have been suggested and are shown in Scheme 80 as summarized by Claflin and co-workers.^136^ Ozonolysis of α-pinene is known to proceed through the addition of O_3_ to the C=C bond, resulting in a primary ozonide 307 that decomposes into excited intermediates (excited Criegee intermediates (ECI 1 and 2) 308 and 309) in the gas phase reactions. In addition, ECI 1 and ECI 2 can be stabilized by N_2_/O_2_ to form stabilized Criegee intermediates like 310, which can react with acid and aldehyde to form hydroperoxyl ester 311 and secondary ozonide 312, respectively. Under humid conditions, the SCI reacts with H_2_O to form hydroxy hydroperoxide 313. In addition, ECI 1 can undergo an isomerization reaction into pinonic acid.^137,138^ ECI 1 and ECI 2 can also undergo decomposition in parallel to produce organoperoxy radicals (314) through vinyl hydroperoxide (VHP) pathways that lead to the formation of putative compounds containing functional groups such as alcohols (315), hydroperoxide (316), peroxycarboxylic acid (317), *etc.*

In the particle phase, the hydroperoxides and peroxycarboxylic acids react with ketones and aldehydes to form peroxy and acylperoxy hemiacetals, leading to carboxylic acids and esters (*via* Baeyer–Villager reactions), alcohols, and aldehydes. In addition, the hydroperoxy esters and hydroxy hydroperoxides from the SCIs are converted to gem-diols and hydroxy esters. Although, as expected, the SOA composition depends on the concentration of oxidizing agents, humidity, and temperature.^139–141^ Ehn and co-workers had shown that HOM formation decreased about 50 percent when experiments were performed at 0 °C compared to 20 °C.^142^ While the molecular formulae and structures of some of the primary products of the photooxidation reactions have been identified, many mechanistic details, reaction intermediates, and reaction end products have not been definitively characterized, but there has been considerable interest in understanding the SOA in recent years.^143–145^ The current state of the art and challenges in molecular characterization of SOA components are reviewed by Nozierre,^146^ and Mahilang,^147^ and their co-workers.

## 8 Conclusion and outlook

Pinene isomers are versatile, cheap, and abundant monoterpene with endless scientific applications, especially in organic synthesis. Pinenes, like many terpenes, are very useful starting materials in constructing complex and straightforward bioactive natural products such as nootkatone and Taxol® in a relatively economical approach. Furthermore, pinene rigid and dimethylated chiral centers at bicyclic bridgehead make it useful for designing chiral ligands and catalysts for asymmetric synthesis. In addition, the presence of di or trisubstituted olefin allows the transformation of pinene through different pathways. This allows the introduction of desirable chirality at methylated carbon or functionalization of methyl at prochiral double bond with boundless opportunity in diversity-oriented synthesis, new method development, and the discovery and development of new natural products-inspired bioactive molecules. Pinenes are renewable, biodegradable, environmentally friendly, and readily available in high abundance from plants. We hope that pinene and other monoterpenes such as camphor, thujane, and menthol will continue to serve as feedstock for developing new synthetic methods and producing cosmetics, polymeric materials, and pharmaceuticals.

## Conflicts of interest

There are no conflicts to declare.

## Supplementary Material
